# Supplementary material for: Programmable site-selective labeling of oligonucleotides based on carbene catalysis
Source: Nat Commun. 2021 Mar 16;12:1681. doi: 10.1038/s41467-021-21839-4 (PMC7966772; doi:10.1038/s41467-021-21839-4)
Supplement: Supplementary file 1 — Supplementary Information [file 41467_2021_21839_MOESM1_ESM.docx]

**Supplementary Information**

**Programmable Site-selective Labeling of Oligonucleotides Based on Carbene Catalysis**

Yang-Ha Lee^1^, Eunsoo Yu^1^, and Cheol-Min Park^1^*

^1^Department of Chemistry, UNIST (Ulsan National Institute of Science & Technology), Ulsan 44919, Korea

*E-mail: cmpark@unist.ac.kr

Table of Contents

1. Comparison of Cu(I) and Rh(I)-based Catalysts 3

2. Supplementary Table 2 – 4 and Figure 2 – 10 12

Supplementary Table 2. Optimization of reaction conditions 12

Supplementary Table 3. Scope for linear ssODNs and ssORNs 13

Supplementary Table 4. Scope for bulge-G duplexes and hairpins 14

Supplementary Figure 2. Comparison of the reactivity between deoxyguanosine and deoxyinosine. 15

Supplementary Figure 3. Modification of 7-deaza-2’-deoxyguanosine. 15

Supplementary Figure 4. Modification of c-di-GMP 16

Supplementary Figure 5. Comparison of G-acetonylation in the absence and presence of 10% THF 16

Supplementary Figure 6. Primer extension assay with acetonylated duplex **8h** 17

Supplementary Figure 7. Modifications under high dilution conditions 18

Supplementary Figure 8. Reaction in the presence of protein 18

Supplementary Figure 9. Single-step synthesis of p-hydroxyphenacyl photocaged ONs **7u** and **7v** 19

Supplementary Figure 10. DNA-protein cross-linking (DPC) by reductive amination with acetonylated dsDNA without binding sequence (T7 promoter) 20

3. General Methods and Procedures 21

4. O^6^-G Acetonylation via Rh(I)-carbenes 26

5. Analysis of Modification Site of Acetonylated Oligonucleotides 31

6. Primer extension assay opposite to O^6^-acetonyl G 32

7. Chemical Ligation of ODNs 33

8. Single-step Synthesis of Photocaged ONs 34

9. DNA-protein Cross-linking 34

10. HPLC-MS Spectra 38

Supplementary Figure 11 – 15. Selected reactions in Table 1 38

Supplementary Figure 16 – 19. Selected reactions in Supplementary Table 2 41

Supplementary Figure 20. Comparison of the reactivity between deoxyguanosine and deoxyinosine 43

Supplementary Figure 21. Modification of 7-deaza-dG with Rh(I)-catalysis 44

Supplementary Figure 22. Modification of N^7^-methyl-2′-deoxyguanosine with Rh(I)-catalysis 45

Supplementary Figure 23. Modification of c-di-GMP with Rh(I)-catalysis 46

Supplementary Figure 24 – 27. Selected reactions in Supplementary Table 3 47

Supplementary Figure 28 – 43. Selected reactions in Table 2 51

Supplementary Figure 51 and 52. Modifications under high dilution conditions 87

Supplementary Figure 53 and 54. Chemical ligation of ODNs 90

Supplementary Figure 55 – 59. Synthesis of photocaged ONs and decaging 94

Supplementary Figure 60. Preparation of Rhodamine B and ketone functionalized oligonucleotides 100

11. NMR Spectra 102

12. DFT Calculations 115

13. Abbreviations 120

14. Supplementary References 121

1. Comparison of Cu(I) and Rh(I)-based Catalysts

The chemoselectivity of Cu(I)-based carbene complexes toward guanosines was reported to arise from the chelation of the catalysts to N^7^ of guanosine^1^. Although chelation-directed selectivity works in favor for simple substrates, it works *against* the desired reaction leading to catalyst deactivation owing to the extensive chelation by substrates if complex oligos are employed. Indeed, the substrate scope in the previous report was limited to relatively simple single stranded oligos.

In contrast, the coordinatively saturated Rh(I)-based catalyst is unable to make chelation, and the catalyst directly react with O^6^ carbonyl group of guanosine by approaching from the other side of N^7^ as supported by the transition state geometry obtained by DFT calculation (Supplementary Fig. 52). Indeed, this theoretical rationale is manifested in the experimental results, where the three systems were compared side-by-side as shown below.


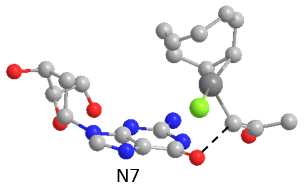


N7

|    |
| --- |
| **Supplementary Figure 1**. Comparison of the Rh(I)-carbene with the reported Cu(I)-carbene conditions |

**[Rh(COD)Cl]_2_ conditions**

**vs. ligandless CuSO_4_ conditions & NHC-Cu(I) conditions** (both from *Chem. Sci.* **8**, 499 (2017))

The results are summarized below (HPLC at 12 h).

- Single stranded oligo: all three conditions show conversion (#1 – 3).
- G-bulge duplex: only Rh(I)-catalyst shows conversion (#4 – 7).
- #7 shows that the superior reactivity of Rh(I)-catalyst is owing to the intrinsic property of the catalyst, not the difference of diazo compounds

| **Supplementary Table 1.** Summary of comparison of Rh(I) and Cu(I)-based catalysis | | | | | |
| --- | --- | --- | --- | --- | --- |
| Entry |  | Substrate | Catalyst | Diazo compound | Yield |
| 1 | Positive control | ss-oligo (**7u**) | [Rh(COD)Cl]_2_ | diazoacetone | 89% |
| 2 |  |  | CuSO_4_ | ethyl diazoacetate | 60% |
| 3 |  |  | NHC-Cu(I) | ethyl diazoacetate | 70% |
| 4 | Test | G-bulge duplex (**18d**) | **[Rh(COD)Cl]_2_** | **diazoacetone** | **90%** |
| 5 |  |  | CuSO_4_ | ethyl diazoacetate | N/R |
| 6 |  |  | NHC-Cu(I) | ethyl diazoacetate | N/R |
| 7 |  |  | NHC-Cu(I) | diazoacetone | N/R |

1. **Positive control, ss-oligo, Rh(I), diazoacetone**

**before reaction**  **7u**

**[M-3H]^3-^**

**1000.2**


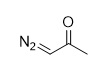

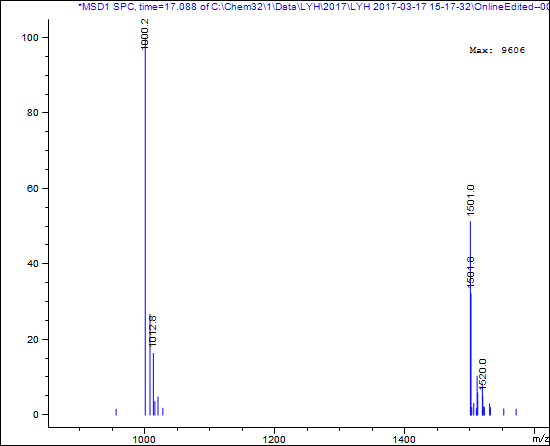


**7u**

**[M-2H]^2-^**

**1501.0**

**at 30 min 8u**

**[M-3H]^3-^**

**1018.8**


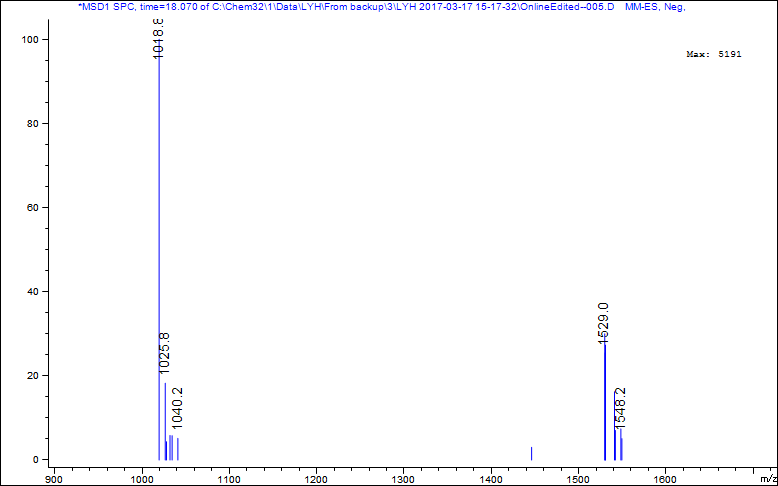


**7u**

**8u**

**[M-2H]^2-^**

**1529.0**

1. **Positive control, ss-oligo, CuSO_4_, ethyl diazoacetate (EDA)**

**before reaction 7u**

**[M-3H]^3-^**

**1000.4**


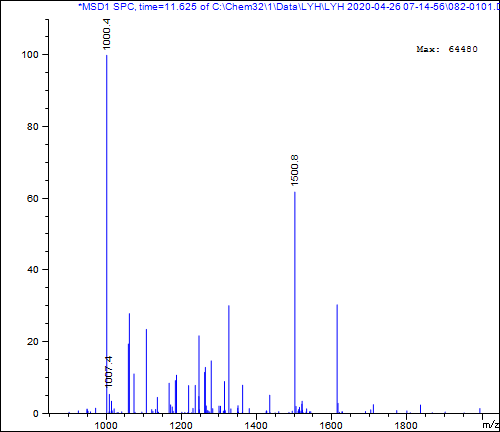


**EDA**

**7u**

**[M-2H]^2-^**

**1500.8**

**at 1 h 8u'** ****
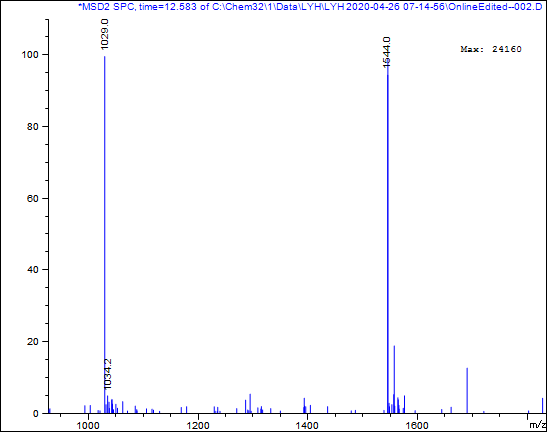


**7u**

**8u'**

**[M-2H]^2-^**

**1544.0**

**[M-3H]^3-^**

**1029.0**

1. **Positive control, ss-oligo, NHC-Cu(I), ethyl diazoacetate (EDA)**

**before reaction 7u**


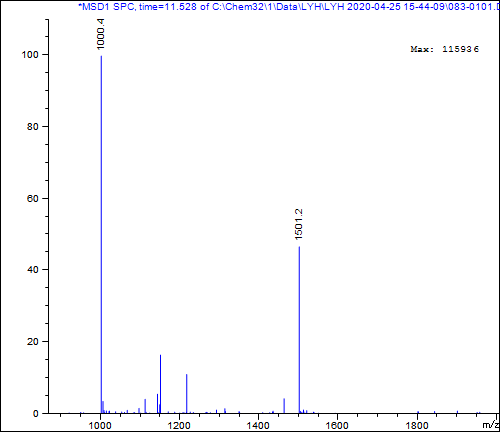


**[M-3H]^3-^**

**1000.4**

**Ascorbate**

**7u**

**8u'**

**7u**

**EDA**

**[M-2H]^2-^**

**1501.2**

**at 1 h 8u**

**[M-3H]^3-^**

**1029.0**

**[M-2H]^2-^**

**1544.4**


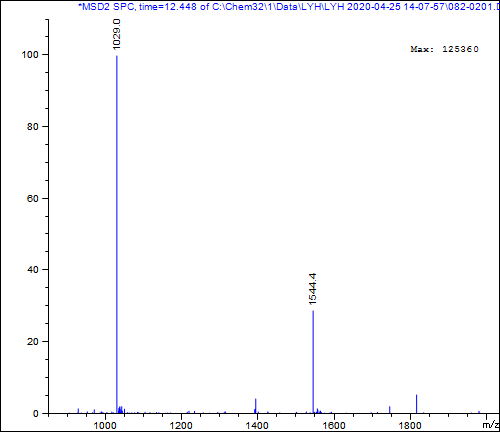


1. **Test, G-bulge duplex, Rh(I), diazoacetone**

**before reaction**


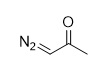


**S1**

**S2**

**oxime ether at 1 h**

**acetonylation at 30 min**

**S1-act, S2**

**S1-OX**

**S2**

*Derivatized to the oxime ether for the separation of overlapping peaks

**oxime ether at 1 h**

**acetonylation at 30 min**

**before reaction**

**S1**  **S1-act**   **S1-OX**

**[M-6H]^6-^**

**1110.2**

**[M-5H]^5-^**

**1311.6**

**[M-5H]^5-^**

**1300.4**


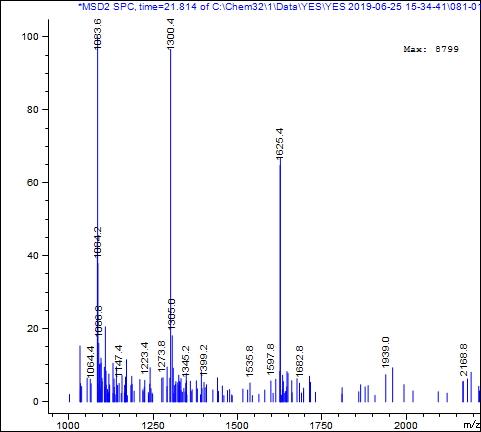

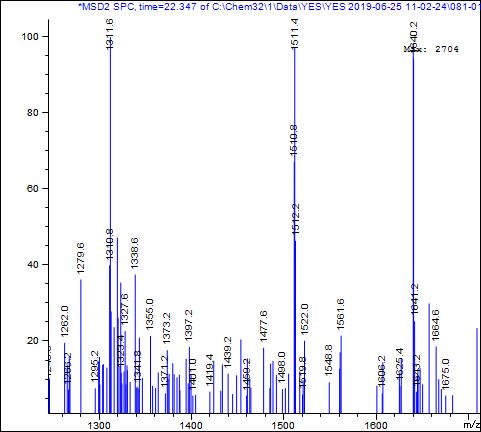

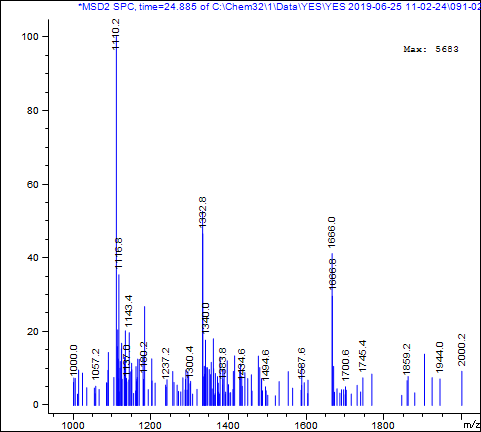


**[M-4H]^4-^**

**1640.2**

**[M-5H]^5-^**

**1332.8**

**[M-4H]^4-^**

**1666.0**

**[M-4H]^4-^**

**1625.4**

**[M-6H]^6-^**

**1083.6**

**S2 S2** **S2**

**[M-4H]^4-^**

**1511.4**


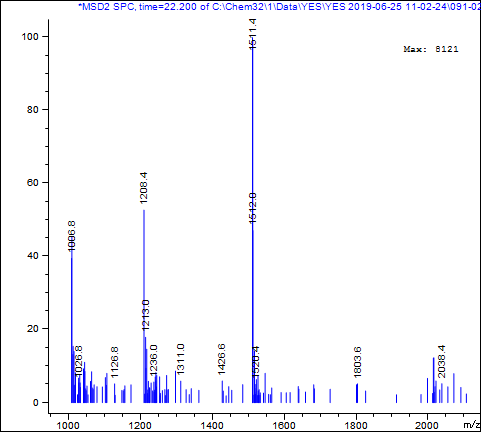

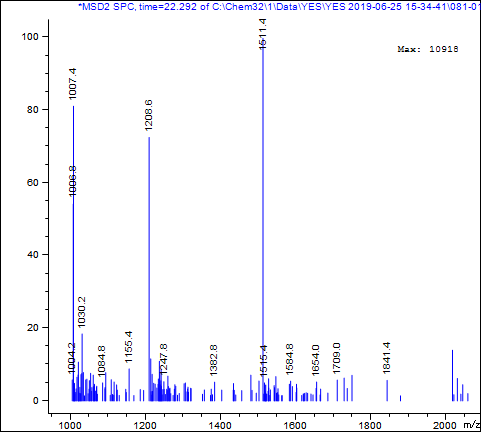

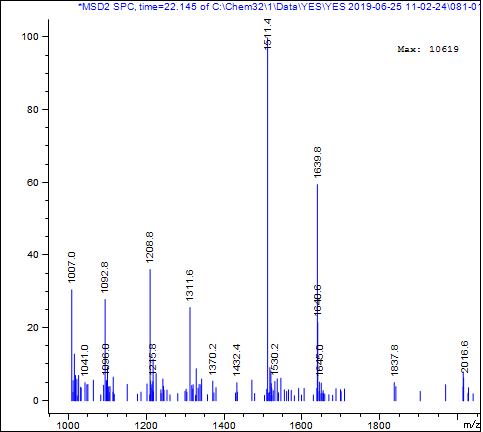


**[M-6H]^6-^**

**1007.4**

**[M-4H]^4-^**

**1511.4**

**[M-5H]^5-^**

**1208.6**

**[M-4H]^4-^**

**1511.4**

**[M-6H]^6-^**

**1006.8**

**[M-5H]^5-^**

**1208.4**

**[M-6H]^6-^**

**1007.0**

**[M-5H]^5-^**

**1208.4**

1. **Test, G-bulge duplex, CuSO4, ethyl diazoacetate (EDA)**

**before reaction**

**S1**

**alkylation at 24 h**

**S1**

**S2**

**EDA**

**EDA**

**S2**

**alkylation at 24 h**

**before reaction**

**S1** **S1**

**[M-4H]^4-^**

**1626.2**


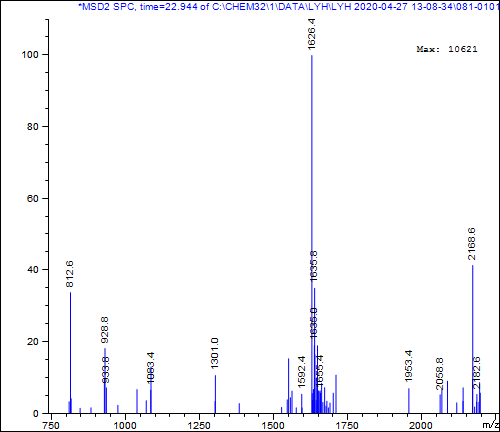

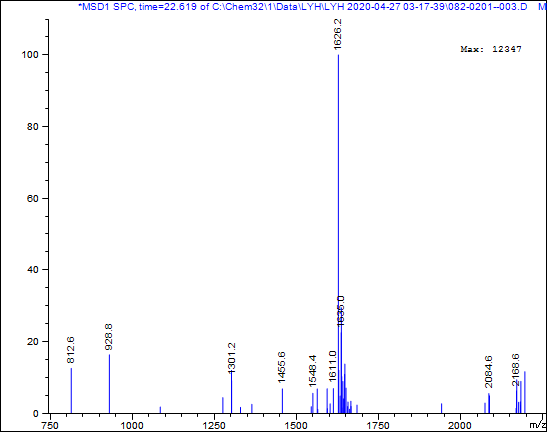


**[M-4H]^4-^**

**1626.4**

**[M-3H]^3-^**

**2168.6**

**[M-3H]^3-^**

**2168.6**


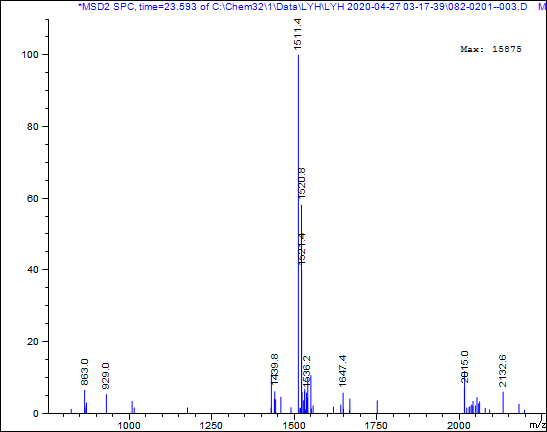

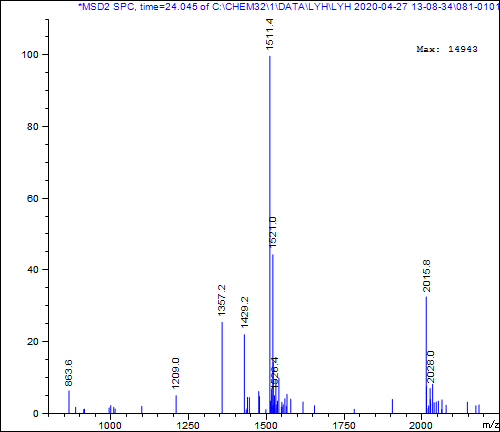
 **S2**  **S2**

**[M-4H]^4-^**

**1511.4**

**[M-4H]^4-^**

**1511.4**

**[M-3H]^3-^**

**2015.8**

**[M-3H]^3-^**

**2015.0**

1. **Test, G-bulge duplex, NHC-Cu(I), ethyl diazoacetate (EDA)**

**before reaction**

**EDA**

**S1**

**S2**

**ascorbate**

**S1**

**alkylation at 24 h**

**S2**

**EDA**

**ascorbate**

**before reaction**

**alkylation at 24 h**

**S1** **S1**


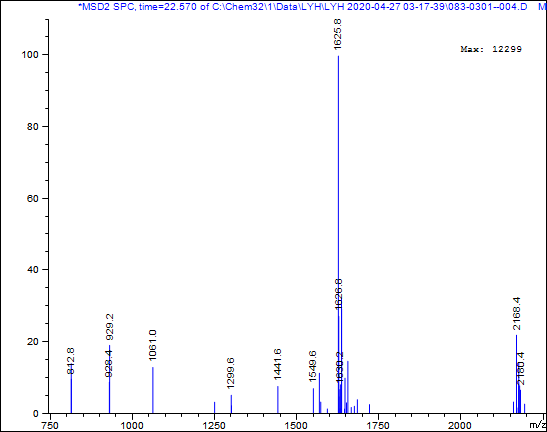

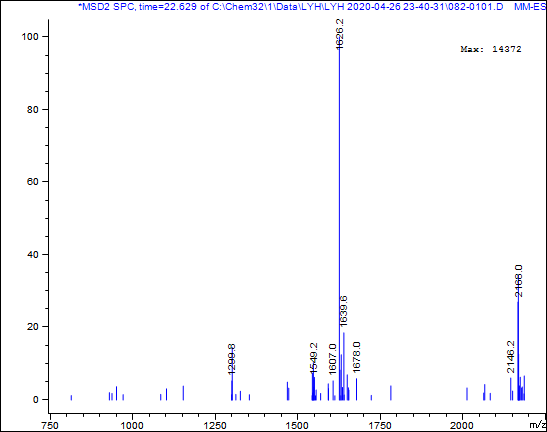


**[M-4H]^4-^**

**1625.8**

**[M-4H]^4-^**

**1626.2**

**[M-3H]^3-^**

**2168.0**

**[M-3H]^3-^**

**2168.4**


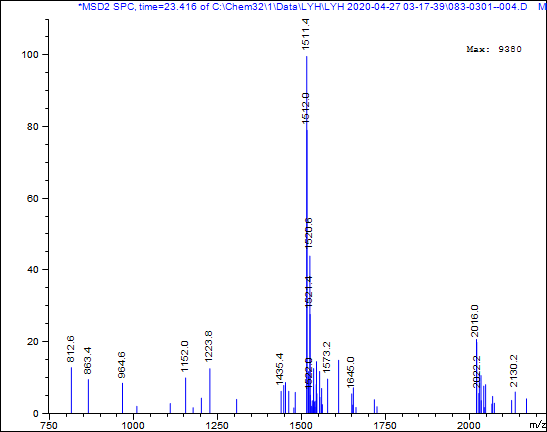

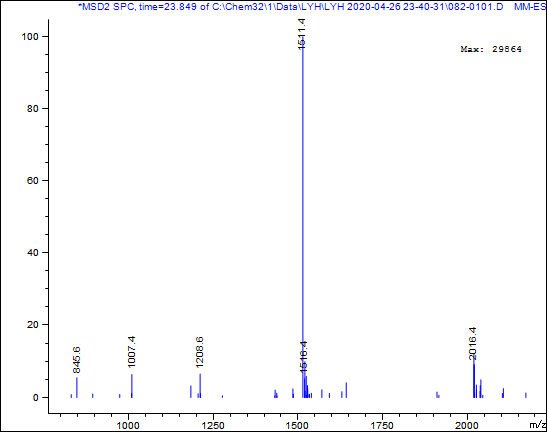
**S2**  **S2**

**[M-4H]^4-^**

**1511.4**

**[M-4H]^4-^**

**1511.4**

**[M-3H]^3-^**

**2016.0**

**[M-4H]^4-^**

**2016.4**

1. **Test, G-bulge duplex, NHC-Cu(I), diazoacetone**

**before reaction**

**ascorbate**


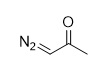


**acetonylation at 24 h**

**ascorbate**

**S1**

**S2**


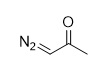


**S1**

**S2**

**acetonylation at 24 h**

**before reaction**


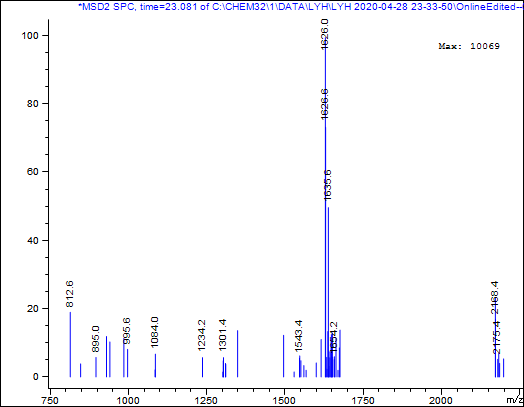
 **S1**   **S1**


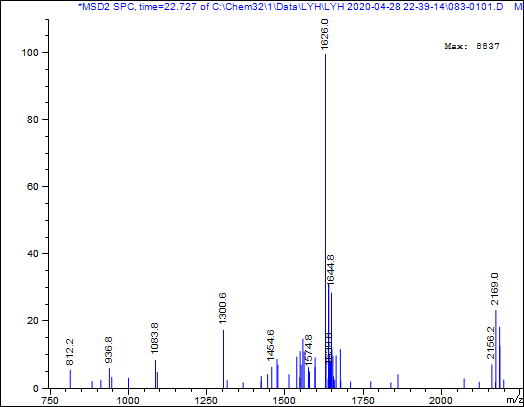


**[M-3H]^3-^**

**2169.0**

**[M-4H]^4-^**

**1626.0**

**[M-4H]^4-^**

**1626.0**

**[M-3H]^3-^**

**2168.4**


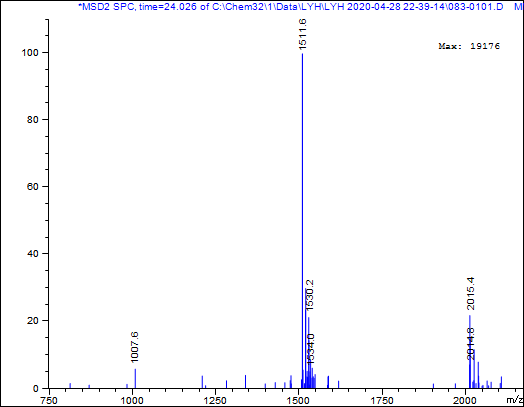

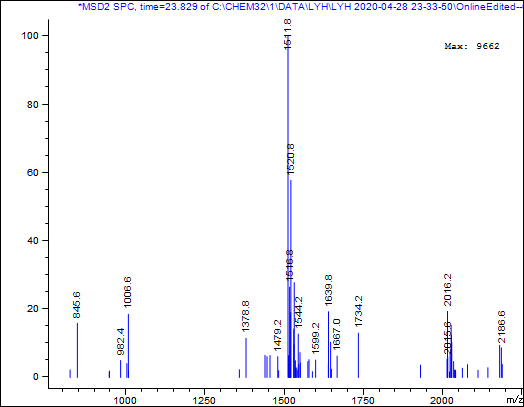
 **S2 S2**

**[M-4H]^4-^**

**1511.8**

**[M-3H]^3-^**

**2016.2**

**[M-4H]^4-^**

**1511.6**

**[M-3H]^3-^**

**2015.4**

2. Supplementary Table 2 – 4 and Figure 2 – 10

Supplementary Table 2. Optimization of reaction conditions

|  | | | | | | |
| --- | --- | --- | --- | --- | --- | --- |
| **diazo compounds**   | | | | | | |
| **Entry**ᵃ | **Substrate** | **Catalyst** | **Diazo compound** | **Buffer** | **Time** | **Yield**^b^ |
| 1^c^ | **1** | [Rh(COD)Cl]_2_ | **5** | - | 90 min | 96% |
| 2 | **1** | [Rh(COD)Cl]_2_ | **5** | - | 90 min | 93% |
| 3 | **1** | [Rh(COD)Cl]_2_ | **5** | MES (20 mM), pH 6.0 | 10 min | 98% |
| 4 | **1** | [Rh(COD)Cl]_2_ | **5** | MOPS (20 mM), pH 7.4 | 10 min | 82% |
| 5^c^ | **1 + 2 + 3 + 4** (1 : 1 : 1 : 1) | Cu(OTf) | **5** | - | 24 h | N/R |
| 6^c^ | **1 + 2 + 3 + 4** (1 : 1 : 1 : 1) | Cu(OTf)_2_ | **5** | - | 24 h | N/R |
| 7^c^ | **1 + 2 + 3 + 4** (1 : 1 : 1 : 1) | Fe(TSPP)Cl | **5** | - | 24 h | N/R |
| 8^c^ | **1 + 2 + 3 + 4** (1 : 1 : 1 : 1) | [Ir(COD)Cl]_2_ | **5** | - | 24 h | N/R |
| 9^c^ | **1 + 2 + 3 + 4** (1 : 1 : 1 : 1) | Ir(COD)(MeCN)_2_BF_4_ | **5** | - | 24 h | N/R |
| 10 | **1 + 2 + 3 + 4** (1 : 1 : 1 : 1) | [Rh(COD)Cl]_2_ | **25a** | MES (20 mM), pH 6.0 | 3 h | N/R |
| 11 | **1 + 2 + 3 + 4** (1 : 1 : 1 : 1) | [Rh(COD)Cl]_2_ | **26a** | MES (20 mM), pH 6.0 | 3 h | N/R |
| 12 | **1 + 2 + 3 + 4** (1 : 1 : 1 : 1) | [Rh(COD)Cl]_2_ | **27a** | MES (20 mM), pH 6.0 | 3 h | N/R |

^a^Conditions a: nucleoside (5 mM), diazo compound (8 equiv.), catalyst (10 mol%), 10% THF-H_2_O, buffer, pH 6.0, r.t.. ^b^Based on HPLC analysis. ^c^Used 50% THF-H_2_O (deviation from Conditions a)

Supplementary Table 3. Scope for linear ssODNs and ssORNs^a^

|  | | | |
| --- | --- | --- | --- |
| **Oligo**^b^ | **Product** | **Time (min)** | **Yield**^c^ |
| **7a** |  | 30 | **8a** (80%) |
| **7r**^d^ |  | 140 | **8r** (57%) |
| **7s**^e^ |  | 300 | N/R |
| **7t** |  | 30 | **8t** (76%) |
| ^a^All ONs are ODNs unless noted as r(sequences) for ORN. ^b^Conditions a: ON (5 mM), diazoacetone (8 equiv.), [Rh(COD)Cl]_2_ (10 mol%), 10% THF-H₂O, MES (20 mM), pH 6.0, Mg(OAc)_2_ (10 mM), r.t. ^c^Based on HPLC analysis. ^d^16 equiv. of diazoacetone, 15 mol% of [Rh(COD)Cl]_2_ (deviation from Conditions a). ^e^16 equiv. of  diazoacetone (deviation from Conditions a). | | | |

| Supplementary Table 4. Scope for bulge-G duplexes and hairpins^a^ | | | |
| --- | --- | --- | --- |
|  | | | |
| **Oligo**^b^ | **Product** | **Time (min)** | **Yield**^c^ |
| **18b** |  | 30 | **19b** (85%) |
| **18c**^d^ |  | 30 | **19c** (71%) |
| **18d**^e^ |  | 30 | **19d** (90%) |
| **18e** |  | 30 | **19e** (88%) |
| **18f**^d,e^ |  | 30 | **19f** (90%) |
| **18g**^d,e^ |  | 30 | **19g** (85%) |
| **18h**^d,e^ |  | 30 | **19h** (90%) |
| ^a^All ONs are ODNs unless noted as r(sequences). ^b^Conditions a: ON (5 mM), diazoacetone (20 equiv.), [Rh(COD)Cl]_2_ (10 mol%), 10% THF-H_2_O, MES (50 mM), pH 6.0, Mg(OAc)_2_ (50 mM), r.t. ^c^Based on HPLC. ^d^40 equiv. of diazoacetone (deviation from Conditions a). ^e^Yield was calculated after oxime ether conjugation with excess amount of benzyloxyamine. ^f^12 equiv. of diazoacetone (deviation from Conditions a). | | | |

|  |
| --- |
| Supplementary Figure 2. Comparison of the reactivity between deoxyguanosine and deoxyinosine. See Supplementary Fig. 20 for HPLC-MS analysis. |

|  |
| --- |
| Supplementary Figure 3. Modification of 7-deaza-2’-deoxyguanosine. See Supplementary Fig. 21 for HPLC analysis. |

|  |
| --- |
| Supplementary Figure 4. Modification of c-di-GMP. See Supplementary Fig. 23 for HPLC-MS analysis. |

|   **H_2_O (without THF)**    **Similar reactivity for both reactions**   - The secondary structures of oligonucleotides appear to be well preserved in the presence of 10% THF.   **8b-OX**  **10% THF-H_2_O**  **7b**  **8b-OX**  **7b** |
| --- |
| Supplementary Figure 5. Comparison of G-acetonylation in the absence and presence of 10% THF. Oxime ether derivatization was performed for HPLC analysis. |

|   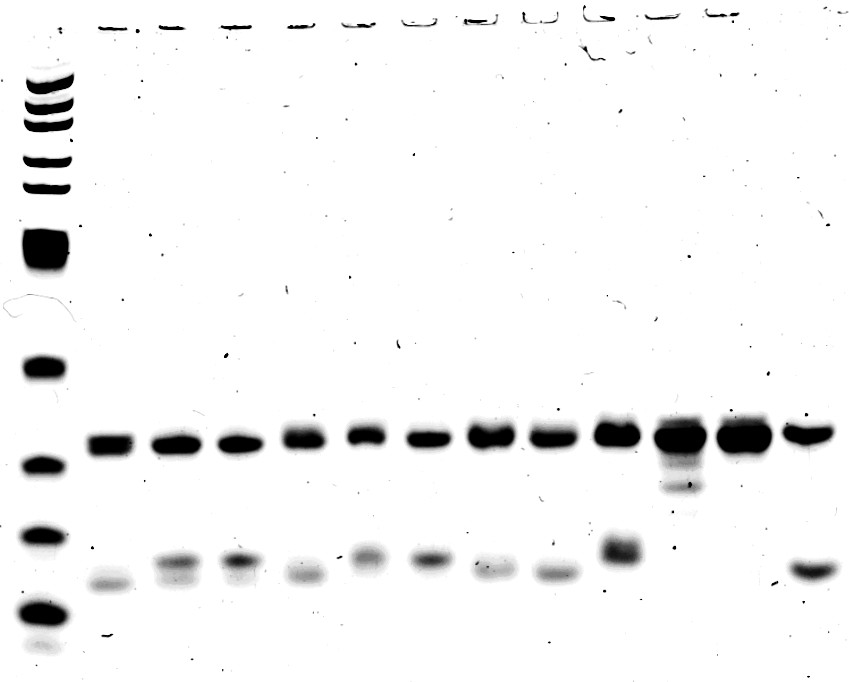  **BP**  **L**  **1 2 3 4 5 6**    **T**    **25**  **20**  **15**  **10**  **P′**  **P**  A T T G C C  **dXTP**  **Reaction time (min)**  60 40 60 60 40 60 |
| --- |
| Supplementary Figure 6. Primer extension assay with acetonylated duplex 8h. Lanes; L: DNA ladder, 1: with dATP at 60 min, 2: with dTTP at 40 min, 3: with dTTP at 60 min, 4: with dGTP at 60 min, 5: with dCTP at 40 min 6: with dCTP at 60 min. Source data are provided as a Source Data file. |

To further examine the site of modification, we performed a primer extension assay with each of the four nucleoside triphosphates (dATP, dTTP, dGTP, and dCTP) by using a template strand **8h** bearing an internal act-G paired with the corresponding primer (Supplementary Fig. 6). While primer extension was not observed with purine bases (A and G, lane 1 and 4), both pyrimidine bases (T and C, lane 2, 3 and 5, 6) were incorporated to give the extended products, indicating that the act-G is recognized as either A or G by the DNA polymerase. This is consistent with previous reports that O^6^-alkylated G elicits G to A transition^2,3^. Increased reaction time up to 1 h gave nearly full conversion using dTTP (lane 3) and dCTP (lane 6). On the other hand, no elongation was observed using dATP and dGTP (lane 1 and 4). These results confirm the site of modification at the unpaired overhang G.

|  |
| --- |
| Supplementary Figure 7. Modifications under high dilution conditions. Reactions at 10 µM concentration. See Supplementary Fig. 51 and 52 for HPLC-MS analysis. |

Bioconjugation reactions often require high dilution owing to the low abundance of target biomolecules, which would negatively affect reaction kinetics. As such, we examined the efficiency of the reaction with ssODN **7u** and bulge duplex **18c** at concentrations as low as 10 µM, and obtained good yields (60% and 59%, respectively, Supplementary Fig. 7).

| **a**    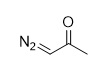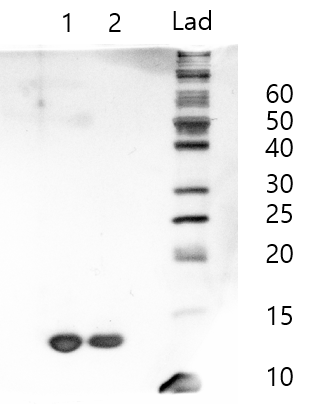**b c**  **1 2 L**  **12% SDS-PAGE**  **lysozyme**  **8a**  **7a**  **7a**  **kDa**  **at 6 h**  **before reaction** |
| --- |
| Supplementary Figure 8. Reaction in the presence of protein. **a**, Scheme of the reaction in the presence of protein. **b**, HPLC analysis of the reaction. **c**, SDS-PAGE analysis to show the presence of protein. Lanes; 1: before reaction, 2: reaction mixture. L: protein standard ladder. Source data are provided as a Source Data file. |

|  |
| --- |
| Supplementary Figure 9. Single-step synthesis of p-hydroxyphenacyl photocaged ONs 7u and 7v. See Supplementary Fig. 57 and 58 for HPLC-MS analysis. |

| **a b**       \| **Lane** \| **1** \| **2** \| **3** \| \| --- \| --- \| --- \| --- \| \| **T7 RNAP** \| + \| + \| + \| \| **Oligo** \| **19i** \| **18i** \| - \|   **c**  **SYBR gold**  **Silver**  T7 RNAP  **1 2 3**  **kDa**  **1 2 3**  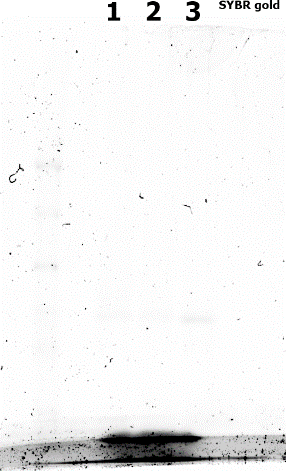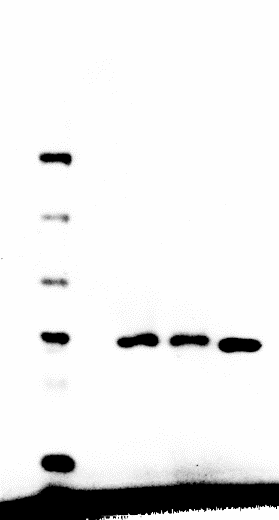  **150**  **200**    **No reaction without binding sequence**  **100**  **120**  **85**  dsDNA  **70** |
| --- | --- | --- | --- | --- | --- | --- | --- | --- | --- | --- | --- | --- |
| Supplementary Figure 10. DNA-protein cross-linking (DPC) by reductive amination with acetonylated dsDNA without binding sequence (T7 promoter). **a**, Schematic representation of the cross-linking. **b**, ON probes employed for **c**. **c**, SDS-PAGE analysis of the cross-linking reaction visualized by silver and SYBR gold stains. Conditions: dsDNA (13.5 µM), T7 RNAP (6.8 µM), HEPES (40 mM), pH 7.4, Mg(OAc)_2_ (5 mM), NaCNBH_3_ (100 mM), 37 ℃, 12 h. Source data are provided as a Source Data file. |

3. General Methods and Procedures

**3.1 General methods**

All the reactions were performed in oven dried glassware under nitrogen atmosphere with freshly distilled dry solvents under anhydrous conditions unless otherwise indicated. Flash column chromatography was performed with Silica Flash P60 silica gel (230 – 400 mesh). All reagents were obtained from commercial sources and were used without further purification. Proton (^1^H) and carbon (^13^C) NMR spectra were recorded on a 400/100 MHz Agilent 400M FT-NMR spectrometer or 400/100 MHz Bruker Advance III HD FT-NMR spectrometer. NMR solvents were obtained from Cambridge Isotope Laboratories and the residual solvent signals were taken as the reference (CDCl_3_, 7.26 ppm, DMSO-d_6_, 2.50 ppm, D_2_O, 4.79 ppm, methanol-d_4_, 3.31 ppm, or acetone-d_6_, 2.05 ppm for ^1^H NMR spectra and CDCl_3_, 77.0 ppm, methanol-d_4_, 49.00 ppm or DMSO-d_6_, 39.52ppm for ^13^C NMR spectra). High resolution mass analysis was performed with JOEL AccuTOF 4G+ DART-HRMS. HPLC-MS analysis was performed using Agilent 6130 quadrupole mass spectrometer coupled with Agilent 1260 Infinity HPLC. Fluorescence spectrum was obtained with Varian Cary Eclipse.

**3.2 Synthetic procedures**

**3.2.1 Synthetic procedures of diazo compounds**


**3-diazopentane-2,4-dione (25a)**

To a solution of acetylacetone (1 g, 10 mmol) of acetonitrile (40 mL) and K₂CO₃ (1.38 g, 11 mmol) was added 4-acetamidobenzenesulfonyl azide (2.4 g, 10 mmol). The mixture was stirred for 5 h at r.t. K₂CO₃ was filtered by celite, using ether (20 mL) as solvent. The filtercake was washed with ether. Sulfonamide was precipitated with hexane : ether = 2 : 1, and filtered once more. The filtrate was concentrated in vacuo and purified by flash column chromatography (20% ethyl acetate-hexane) to yield **25a** as a yellow oil (790 mg, 79% yield). ^1^H NMR (400 MHz, CDCl3): δ 2.44 (s, 6H). The structure of the compound was further confirmed by the comparison of the spectral data in the literature^4^.

**diazoacetone (5)**

To a solution of **25a** (504 mg, 4 mmol) in diethyl ether (20 mL), 1 N NaOH solution (20 mL) was added slowly and the mixture was stirred at r.t. for 1 h. H_2_O (10 mL) was added and the aqueous phase was extracted with DCM (60 x 3 mL). The combined organic layer was dried over Na_2_SO_4_, filtered, and the filtrate was concentrated under reduced pressure (8 ℃, > 165 torr). Solvent was dried further in vacuo to afford **5** as a volatile yellow oil (293 mg, 87% yield). ^1^H NMR (400 MHz, CDCl_3_): δ 5.26 (br s, 1H), 2.10 (s, 3H). The structure of the compound was further confirmed by the comparison of the spectral data in the literature^5^.

**methyl 2-diazo-3-oxobutanoate (26a)**

To a solution of methyl acetoacetate (581 mg, 5 mmol) in acetonitrile (25 mL), K₂CO₃ (690 mg, 5.5 mmol) was added slowly, followed by tosyl azide (986 mg, 5 mmol). The mixture was stirred for 16 h at r.t. K₂CO₃ was filtered by silica gel by using ethyl acetate and concentrated in vacuo. The crude material was purified by flash column chromatography (5 – 20% ethyl acetate-hexane) to yield **26a** (310 mg, 53% yield). ^1^H NMR (400 MHz, CDCl_3_): δ 3.85 (s, 3H), 2.49 (s, 3H). The structure of the compound was further confirmed by the comparison of the spectral data in the literature^6^.

**dimethyl 2-diazomalonate (27a)**

To a solution of dimethylmalonate (650 mg, 5 mmol) and tosyl azide (1.2 g, 5.1 mmol) in acetonitrile (25 mL) at 0 ℃, triethylamine (2.1 mL, 15 mmol) was added. The mixture was warmed to r.t. and stirred for 12 h. The mixture was concentrated in vacuo. The concentrated mixture was triturated with ethyl acetate-hexane, filtered, and concentrated in vacuo. The crude material was purified by flash column chromatography (10 – 30% ethyl acetate-hexane), to afford **27a** (647 mg, 81% yield) as yellow liquid. ^1^H NMR (400 MHz, CDCl_3_): 3.84 (s, 6H). The structure of the compound was further confirmed by the comparison of the spectral data in the literature^7^.

**4-(2-bromoacetyl)phenyl acetate (28a)**

To a solution of 2-bromo-1-(4-hydroxyphenyl)ethan-1-one (150 mg, 0.7 mmol) in dry THF (2.3 mL) at 0 ℃ was added acetic anhydride (86 μL, 0.91 mmol) and K_2_CO_3_ (194 mg, 1.4 mmol). The mixture was slowly warmed to r.t. and stirred for 2 h. To the mixture was added water (3 mL) and extracted with diethyl ether. The combined organic layers were dried over Na_2_SO_4_, filtered, and concentrated in vacuo. The crude material was purified by flash column chromatography (1 – 10% diethyl ether-toluene) to yield **28a** (131 mg, 73% yield) as a clear oil. ^1^H NMR (400 MHz, CDCl_3_): δ 8.06-8.00 (m, 2H), 7.26-7.22 (m, 2H), 4.43 (s, 2H), 2.34 (s, 3H). The structure of the compound was further confirmed by the comparison of the spectral data in the literature^8^.

**4-(2-diazoacetyl)phenyl acetate (28b)**

To a solution of **28a** (52 mg, 0.2 mmol), N,N’-ditosylhydrazine (89 mg, 0.26 mmol) in dry THF (1 mL) at 0 ℃ was added DBU (90 µL, 0.6 mmol). The mixture was stirred for 1 h and was added saturated NaHCO_3_ solution (1 mL), and water (1 mL). The aqueous layer was extracted with diethyl ether, and combined organic layers were dried over Na_2_SO_4_, and filtered, and concentrated in vacuo. The crude material was purified by flash chromatography (5 – 30% ethyl acetate-hexane) to yield **28b** (30 mg, 74% yield) as yellow solid. ^1^H NMR (400 MHz, CDCl_3_): δ 7.82-7.77 (m, 2H), 7.21-7.17 (m, 2H), 5.87 (s, 1H), 2.32 (s, 3H). The structure of the compound was further confirmed by the comparison of the spectral data in the literature^9^.

**2-diazo-1-(4-hydroxyphenyl)ethan-1-one (16)**

To a solution of **28b** (29 mg, 0.142 mmol) in methanol (4 mL) and water (1 mL), was added NH_4_OAc (88 mg, 1.14 mmol). The mixture was stirred for 10 h at 40 ℃ and concentrated in vacuo. To the mixture was added water (2 mL) and the aqueous layer was extracted with diethyl ether. Combined organic layers were dried over Na_2_SO_4_, and filtered, and concentrated in vacuo to yield **16** (19 mg, 82% yield) as yellow solid. ^1^H NMR (400 MHz, acetone-d_6_): δ 9.23 (br s, 1H), 7.78-7.73 (m, 2H), 6.93-6.89 (m, 2H), 6.48 (s, 1H). The structure of the compound was further confirmed by the comparison of the spectral data in the literature^9^.

**3.2.2 Synthetic procedures of a Rhodamine B–aminooxy derivative 29d**

**tert-butyl ((6-bromohexyl)oxy)carbamate (29a)**

****To a solution of BocNHOH (150 mg, 1.13 mmol) in DMF (2.26 mL), DBU (200 μL, 1.356 mmol) was added. Then, 1,6-dibromohexane (350 μL, 2.26 mmol) and tetrabutylammonium bromide (36 mg, 0.113 mmol) were added to the solution, and the solution was stirred for 12 h at r.t. The mixture was concentrated in vacuo and purified by flash column chromatography. (5~20% ethyl acetate - hexane) to yield **29a** as pale-yellow liquid (119 mg, 35% yield). ^1^H NMR (400 MHz, CDCl_3_): δ 7.09 (s, 1H), 3.85 (t, 2H), 3.41 (t, 2H), 1.86 (p, 2H), 1.64 (p, 2H), 1.52~1.37 (m, 13H). The structure of the compound was further confirmed by the comparison of the spectral data in the literature^10^.

**tert-butyl ((6-(piperazin-1-yl)hexyl)oxy)carbamate (29b)**

tert-butyl ((6-bromohexyl)oxy)carbamate (119 mg, 0.401 mmol) and piperazine (138 mg) were dissolved in acetonitrile (5.7 mL), and stirred at r.t. for 12 h. The reaction mixture was concentrated in vacuo and dissolved in diethyl ether (10 mL) and brine (10 mL). The aqueous solution was extracted with diethyl ether 3 times (3 x 20 mL). Combined organic layers were dried over MgSO_4_, filtered, and concentrated in vacuo to afford **29b** as yellow liquid (120 mg, 99% yield), which was used in the next step without further purification. ^1^H NMR (400 MHz, CDCl_3_): δ 7.24 (s, 1H), 3.84 (t, *J* = 6.6 Hz, 2H), 2.90 (t, *J* = 4.9 Hz, 4H), 2.40 (br s, 4H), 2.30 (m, 2H), 1.62 (m, 2H), 1.48 (m, 11H), 1.35 (m, 5H), 1.26 (s, 1H). The structure of the compound was further confirmed by the comparison of the spectral data in the literature^10^.

**N-(9-(2-(4-(6-(((tert-butoxycarbonyl)amino)oxy)hexyl)piperazine-1-carbonyl)phenyl)-6-(diethylamino)-3H-xanthen-3-ylidene)-N-ethylethanaminium (29c)**

To a solution of Rhodamine B (41 mg, 0.086 mmol) and triphenylphosphine (34 mg, 0.129 mmol) in dry DCM (0.6 mL) cooled to 0 ℃, NBS (27 mg, 0.151 mmol) was added to the solution and the mixture was stirred for 15 min. tert-butyl ((6-(piperazin-1-yl)hexyl)oxy)carbamate (13 mg, 0.043 mmol) and pyridine in DCM (0.8 mL) were added and the reaction was brought to r.t. and stirred overnight. The solvent was then removed under reduced pressure, and the residue partitioned between ethyl acetate (20 mL) and saturated aqueous NaHCO_3_ (10 mL). The aqueous layer was extracted with ethyl acetate (15 x 3 mL), and the combined organic layers were dried over MgSO_4_ and concentrated in vacuo. The crude material was purified by flash column chromatography (5-10% methanol - dichloromethane) to give **29c** (14 mg, 47% yield) as red solid. ^1^H NMR (400 MHz, MeOH-d_4_): δ 7.76 (m. 2H), 7.65 (m, 1H), 7.52 (m, 1H), 7.28 (d, *J* = 9.5 Hz, 1H), 7.07 (dd, *J* = 9.6, 2.5 Hz, 1H), 6.98 (d, *J* = 2.4 Hz, 1H), 3.80 – 3.64 (m, 10H), 3.59 (s, 2H), 3.39 (t, *J* = 5.1 Hz, 4H), 2.28 – 2.13 (m, 6H), 1.60 (m, 2H), 1.35 - 1.46 (m, 11H), 1.31 (t, *J* = 7.1 Hz, 12H). The structure of the compound was further confirmed by the comparison of the spectral data in the literature^10^.

**N-(9-(2-(4-(6-(aminooxy)hexyl)piperazine-1-carbonyl)phenyl)-6-(diethylamino)-3H-xanthen-3-ylidene)-N-ethylethanaminium trifluoroacetate (29d)**

N-(9-(2-(4-(6-(((tert-butoxycarbonyl)amino)oxy)hexyl)piperazine-1-carbonyl)phenyl)-6-(diethylamino)-3H-xanthen-3-ylidene)-N-ethylethanaminium (10 mg) was dissolved in 50% TFA-DCM (2 mL), and the solution was stirred for 2 h. The solution was concentrated and re-dissolved in DCM repeatedly 2~3 times to give **29d** (quantitative) as purple solid. The concentrated purple solid **29d** was used for fluorophore labeling without further purification. ^1^H NMR (400 MHz, MeOH-d_4_): δ 7.79 (m, 2H), 7.75 (m, 1H), 7.53 (m, 1H), 7.25 (d, *J* = 9.2 Hz, 2H), 7.07 (dd, *J* = 9.5, 2.4 Hz, 2H), 6.97 (m, 2H), 4.01 (m, 2H), 3.75 – 3.35 (m, 12H), 3.28 – 3.05 (m, 4H), 1.71 (m, 4H), 1.42 (m, 4H), 1.31 (t, *J* = 7.1 Hz, 12H). The structure of the compound was further confirmed by the comparison of the spectral data in the literature^10^.

**3.2.3 Synthetic procedures of dialkoxyamine linker 14**

**Oxybis(ethane-2,1-diyl) bis(4-methylbenzenesulfonate) (30a)**

To a solution of diethylene glycol (159 mg, 1.5 mmol) in pyridine (3 mL) at 0 ℃ was added p-toluenesulfonyl chloride (572 mg, 3 mmol). The mixture was warmed to r.t. and was stirred for 8 h. The mixture was concentrated in vacuo and was added brine (5 mL) and the aqueous layer was extracted with dichloromethane. Combined organic layers were dried over Na_2_SO_4_, and filtered, and concentrated in vacuo to yield **30a** (523 mg, 84% yield) as a white solid. ^1^H NMR (400 MHz, CDCl_3_): δ 7.78 (d, *J* = 8.3 Hz, 4H), 7.35 (d, *J* = 8.0 Hz, 4H), 4.09 (m, 4H), 3.61 (m, 4H), 2.45 (s, 6H). The structure of the compound was further confirmed by the comparison of the spectral data in the literature^11^.

**di-tert-butyl ((oxybis(ethane-2,1-diyl))bis(oxy))dicarbamate (30b)**

To a solution of **30a** (26 mg, 0.062 mmol) and tert-butyl hydroxycarbamate (23 mg, 0.17 mmol) in THF (0.6 mL) at 0 ℃ was added NaH (6.4 mg, 0.16 mmol, 60% dispersion in mineral oil). The mixture was warmed to r.t. and stirred for 12 h. To the mixture was added a saturated solution of NH_4_Cl (3 mL), and the aqueous layer was extracted with ethyl acetate. Combined organic layers were dried over Na_2_SO_4_, and filtered, and concentrated in vacuo. The crude material was purified by flash chromatography (5-40% ethyl acetate - hexane) to yield **30b** (14 mg, 68% yield) as a clear oil. ^1^H NMR (400 MHz, CDCl_3_): δ 7.61 (br s, 2H), 4.04 (m, 4H), 3.73 (m, 4H), 1.48 (s, 18H). ^13^C NMR (100 MHz, CDCl_3_): δ 156.85, 81.63, 75.27, 68.84, 28.20. MS (APCI, M+H^+^): m/z found 337.2

**O,O'-(oxybis(ethane-2,1-diyl))dihydroxylammonium chloride (14)**

A solution of **30b** (14 mg, 0.042 mmol) in 4 N HCl-dioxane (0.8 mL) at r.t. was stirred for 10 h. The mixture was concentrated in vacuo and washed several times with diethyl ether to yield **14** as a white solid (8 mg, 90% yield). ^1^H NMR (400 MHz, D_2_O): δ 4.26 (m, 4H), 3.86 (m, 4H). ^13^C NMR (100 MHz, D_2_O): δ 73.86, 68.35. HRMS (ESI, M+H^+^): m/z calcd. for C_4_H_13_N_2_O_3_^+^ 137.0921, found 137.0921.

4. O^6^-G Acetonylation via Rh(I)-carbenes

**4.1 Optimization of Rh(I)-catalyzed O^6^-G acetonylation**

**General Procedure A:** All reactions were performed in PCR tubes in a total volume of 20 μL. An aqueous solution of nucleosides (each 100 nmol) in a PCR tube was concentrated, and the nucleosides resuspended in water (12 μL) was treated with an aqueous solution of MES buffer (4 μL, 100 mM, pH 6.0), and an aqueous solution of diazoacetone (2 μL, 400 mM). A solution of Rh(I) catalyst in THF (2 μL, 5 mM) was added and mixed thoroughly. The reaction mixture was incubated at r.t. for 0.5 h unless otherwise noted and analyzed by HPLC-MS.

**4.1.1 Screening of various metal catalysts with diazoacetone using a mixture of 4 nucleoside monomers (dA, dT, dG, and dC)**

The reaction was performed with several modifications from **General Procedure A**; using nucleoside monomers (dA, dT, dG, dG, each 100 nmol), water (10 μL), additional THF (6 μL), diazoacetone in THF (2 μL, 400 mM), metal catalyst in THF (2 μL, 5 mM), incubation 24 h, without MES buffer.

**4.1.2 Rh(I)-catalyzed O^6^-acetonylation of deoxyguanosine in aqueous 50% THF**

The reaction was performed with several modifications from **General Procedure A**; using deoxyguanosine (100 nmol), water (10 μL), additional THF (6 μL), diazoacetone in THF (2 μL, 400 mM), without MES buffer.

**4.1.3 Rh(I)-catalyzed O^6^-acetonylation of deoxyguanosine in aqueous 10% THF**

The reaction was performed with several modifications from **General Procedure A**; using deoxyguanosine (100 nmol), water (16 μL), without MES buffer.

**4.1.4 Rh(I)-catalyzed O^6^-acetonylation of deoxyguanosine in buffer**

The reaction was performed with **General Procedure A** using deoxyguanosine (100 nmol).

**4.1.5 Rh(I)-catalyzed O^6^-acetonylation of deoxyguanosine characterized by NMR spectroscopy**

To a solution of deoxyguanosine (20 mg, 0.075 mmol) in MES (0.3 mL, 1 M) and water (13.2 mL), diazoacetone (50 mg, 0.60 mmol) was added to the solution, followed by [Rh(COD)Cl]_2_ (3.69 mg, 0.0075 mmol) in THF (1.5 mL). The reaction mixture was stirred for 30 min at r.t., after which the reaction mixture was concentrated under reduced pressure. The crude mixture was purified by flash column chromatography to give O^6^-acetonylated deoxyguanosine **15a** (18 mg, 66%). The isolated product was characterized with ^1^H, ^13^C NMR and HRMS.

^1^H NMR (400 MHz, DMSO-d_6_): δ 8.12 (s, 1H), 6.41 (s, 2H), 6.21 (dd, *J* = 7.7, 6.0 Hz, 1H), 5.29 (s, 1H), 5.06 (s, 2H), 5.00 (s, 1H), 4.36 (m, 1H), 3.83 (td, *J* = 4.6, 2.7 Hz, 1H), 3.54 (dtd, *J* = 16.3, 11.5, 4.5 Hz, 2H), 2.59 (ddd, *J* = 13.3, 7.8, 5.7 Hz, 1H), 2.22 (ddd, *J* = 13.1, 6.0, 3.1 Hz, 1H), 2.16 (s, 3H)
^13^C NMR (100 MHz, DMSO-d_6_): δ 202.89, 159.41, 159.32, 154.22, 138.13, 113.65, 87.64, 82.84, 70.77, 69.41, 61.73, 26.17, HRMS (ESI, M+Na^+^): m/z calcd. for C_13_H_17_N_5_NaO_5_^+^, 346.1122, found, 346.1122.

**4.1.6 Screening of Rh(I) catalysts for O^6^-acetonylation of deoxyguanosine using a mixture of 4 nucleoside monomers (dA, dT, dG, and dC)**

The reaction was performed with **General Procedure A** using nucleoside monomers (dA, dT, dG, dC, each 100 nmol).

**4.1.7 Reactivity comparison between deoxyguanosine and deoxyinosine (dG and dI) by Rh(I)-catalysis**

The reaction was performed with **General Procedure A** using deoxyguanosine and deoxyinosine (each 100 nmol).

**4.1.8 Rh(I)-catalysis to 7-deaza-2’-deoxyguanosine in buffer**

The reaction was performed with 7-deaza-2’-deoxyguanosine (100 nmol) following **General Procedure A**.

**4.1.9 Rh(I)-catalyzed O^6^-acetonylation of N^7^-methyl-2’-deoxyguanosine in buffer**

The reaction was performed following **General Procedure A** with modification; using N^7^-methyl-2’-deoxyguanosine (100 nmol), diazoacetone in water (4 μL, 400 mM), water (10 μL), incubation for 1.5 h. NMR analysis was performed after sugar was hydrolytically removed

**4.1.10 Rh(I)-catalyzed O^6^-acetonylation of c-di-GMP in buffer**

Reaction was performed in PCR tubes in a total volume of 20 μL. An aqueous solution of c-di-GMP (20 nmol) was treated with an aqueous solution of MES buffer (2 μL, 100 mM, pH 6.0), Mg(OAc)_2_ (2 μL, 50 mM), and diazoacetone (8 μL, 10 mM). A solution of Rh(I) catalyst in THF (2 μL, 1 mM) was added and mixed thoroughly. The reaction mixture was incubated at r.t. for 0.5 h unless otherwise noted and analyzed by HPLC-MS.

**4.2 Synthesis of oligonucleotides**

**4.2.1 DNA synthesis**

Solid-phase synthesis of oligodeoxyribonucleotides were performed with the Bioautomation Mermade 4 according to the manufacturer protocol using the standard phosphoramidite chemistry. Purification of oligonucleotides were performed using the Agilent 1260 Infinity preparative HPLC with the ZORBAX 300SB-C18 PrepHT column at a flow rate of 15 mL/min. A gradient of acetonitrile with 20 mM triethylammonium acetate (pH 7.0) was used as an eluent. The collected DMT-on fractions were concentrated with the Thermo Scientific SPD131DDA SpeedVac Concentrator. Deprotection of dimethoxytrityl (DMT) groups and further purification was performed using the Glen-Pak DNA purification cartridge. Purified oligonucleotides were characterized by the Agilent 6130 quadrupole mass spectrometer. Concentrations of purified oligonucleotides were analyzed with the Thermo Scientific Nanodrop-One at 260 nm.

**4.2.2 RNA synthesis**

Solid-phase synthesis of oligoribonucleotides were performed with the Bioautomation Mermade 4 according to the manufacturer protocol using the standard phosphoramidite chemistry. Deprotection of bases and phosphates was performed in AMA (concentrated NH_3_ : 40% aqueous MeNH_2_ = 1 : 1) solution at 65 ℃ for 1.5 h. 2’-TBDMS groups were removed by TEA·3HF at 65 ℃ for 2.5 h. Purification of oligonucleotides were performed using the Agilent 1260 Infinity preparative HPLC with the ZORBAX 300SB-C18 PrepHT column at a flow rate of 15 mL/min. A gradient of acetonitrile with 20 mM triethylammonium acetate (pH 7.0) was used as an eluent. The collected DMT-on fractions were concentrated with the Thermo Scientific SPD131DDA SpeedVac Concentrator. Deprotection of dimethoxytrityl (DMT) groups and further purification was performed using the Glen-Pak DNA purification cartridge. Purified oligonucleotides were characterized by the Agilent 6130 quadrupole mass spectrometer. Concentrations of purified oligonucleotides were analyzed with the Thermo Scientific Nanodrop-One at 260 nm.

**4.3 Rh(I)-catalyzed O^6^-G acetonylation of ODNs or ORNs**

**General Procedure B:** All reactions were performed in PCR tubes in a total volume of 10 μL. An aqueous solution of ODN or ORN (50 nmol) in a PCR tube was concentrated, and the ON resuspended in water (4 μL) was treated with an aqueous solution of Mg(OAc)_2_ (2 μL, 50 mM), MES buffer (2 μL, 100 mM, pH 6.0), and an aqueous solution of diazoacetone (1 μL, 400 mM). A solution of [Rh(COD)Cl]_2_ in THF (1 μL, 5 mM) was added and mixed thoroughly. The reaction mixture was incubated at r.t. for 0.5 h unless otherwise noted and analyzed by HPLC-MS.

Derivatization of the acetonylated products by oxime ether conjugation with benzyloxyamine was performed when partial separation of HPLC peaks was observed. The crude material from the reaction was diluted with water (20 μL), extracted with ethyl acetate (30 μL x 3), and the resulting aqueous layer was concentrated. The crude material was dissolved in water, and a small portion (5 nmol of dsDNA) was diluted with water to a final volume of 4 μL. The solution was treated with benzyloxyamine (1 μL, 50 mM in DMSO), kept at r.t. for 1 h (unless otherwise noted), and analyzed by HPLC-MS.

**4.3.1 Rh(I)-catalyzed O^6^-G acetonylation of linear ssODN, ssORN, hairpin and duplex**

The reaction was performed with **General Procedure B**.

**4.3.2 Rh(I)-catalyzed O^6^-G acetonylation of bulge dsODNs and ORN-ODN hybrid duplex**

The reaction was performed with several modifications from **General Procedure B**; water (2.5 μL), Mg(OAc)_2_ (2 μL, 250 mM), MES buffer (2 μL, 250 mM, pH 6.0), and diazoacetone in H_2_O (2.5 μL, 400 mM).

**4.3.3 Rh(I)-catalyzed O^6^-G acetonylation of ODNs in the absence of organic solvent**

All reactions were performed in PCR tubes in a total volume of 10 μL. An aqueous solution of ODN (50 nmol) in a PCR tube was concentrated, and the ON resuspended in water (5 μL) was treated with an aqueous solution of Mg(OAc)_2_ (2 μL, 50 mM), MES buffer (2 μL, 100 mM, pH 6.0), and an aqueous solution of diazoacetone (1 μL, 400 mM). The mixture was added to another PCR tube containing [Rh(COD)Cl]_2_ (5 nmol, dried from 5 mM THF solution 1 μL) and mixed thoroughly. The reaction mixture was incubated at r.t. for 0.5 h unless otherwise noted and analyzed by HPLC-MS.

**4.3.4 Rh(I)-catalyzed O^6^-G acetonylation of ODNs under dilute oligonucleotide concentration**

All reactions were performed in PCR tubes in a total volume of 50 μL. An aqueous solution of ODN or ORN (1 nmol) in a PCR tube was concentrated, and the ON resuspended in water (48.5 μL) was treated with an aqueous solution of Mg(OAc)_2_ (20 μL, 50 mM), MES buffer (20 μL, 100 mM, pH 6.0), and an aqueous solution of diazoacetone (1.5 μL, 400 mM). Next, a half amount (45 μL) of the mixture was taken from the batch to be used as HPLC-MS sample of starting mixture. To the remaining mixture, a solution of [Rh(COD)Cl]_2_ in THF (1 μL, 5 mM) was added and mixed thoroughly. The reaction mixture was incubated at r.t. for 3 h unless otherwise noted and analyzed by HPLC-MS.

**4.3.5 Rh(I)-catalyzed O^6^-G acetonylation of ODN in the presence of lysozyme**

All reactions were performed in PCR tubes in a total volume of 30 μL. An aqueous solution of ODN (12.5 nmol) in a PCR tube was concentrated, and the ON resuspended in water (25 μL) was treated with an aqueous solution of Mg(OAc)_2_ (5 μL, 50 mM), MES buffer (5 μL, 100 mM, pH 6.0), and an aqueous solution of diazoacetone (2.5 μL, 100 mM). Next, a half amount (20 μL) of the mixture was taken from the batch to be used as HPLC-MS sample of starting mixture. To the remaining mixture, a solution of [Rh(COD)Cl]_2_ in THF (3 μL, 0.25 mM) was added and mixed thoroughly. The reaction mixture was incubated at r.t. for 1 h unless otherwise noted and analyzed by HPLC-MS and 12% SDS-PAGE (30 : 1 = acrylamide : bisacrylamide, 1x SDS Tris-Cl buffer) with 180 V.

**4.4 Cu(I)-catalyzed alkylation of ODNs**

**General Procedure C**: The reaction was performed in PCR tubes in a total volume of 10 μL. An aqueous solution of ODN (50 nmol) in a PCR tube was concentrated, and the ODN resuspended in water (3.5 μL) was treated with an aqueous solution of MES buffer (2 μL, 500 mM, pH 6.0), CuSO_4_ (2 μL, 5 mM), and an DMSO solution of ethyl diazoacetate (2 μL, 250 mM). A solution of sodium ascorbate in H_2_O (0.5 μL, 100 mM) was added and mixed thoroughly. The reaction mixture was incubated at r.t. for 0.5 h and analyzed by HPLC-MS.

**4.4.1** **Cu(I)-catalyzed alkylation of ODN by using EDA**

The reaction was performed with **General Procedure C**.

**4.4.2 NHC-Cu(I)-catalyzed alkylation of ODN by using EDA**

The reaction was performed with several modifications from **General Procedure C**; water (4 μL), DME (1 μL), sodium ascorbate in H_2_O (1 μL, 100 mM), ethyl diazoacetate in DME (1 μL, 500 mM), and IMesCuCl in DME (1 μL, 10 mM).

**4.4.3 NHC-Cu(I)-catalyzed alkylation of ODN by using diazoacetone**

The reaction was performed with several modifications from **General Procedure C**; water (4 μL), DME (1 μL), sodium ascorbate in H_2_O (1 μL, 100 mM), diazoacetone in DME (1 μL, 500 mM), and IMesCuCl in DME(1 μL, 10 mM).

**4.5 Purification of O^6^-G acetonylated ODNs**

After the acetonylation was completed, the reaction mixture was purified by the following protocol for next experiments, unless otherwise noted. To the reaction mixture was added water (20 μL), and the mixture was vigorously washed with ethyl acetate (30 μL, 3 times). Combined organic layers were discarded, and the aqueous layer was concentrated. The dried crude material was further purified with the Glen-Park DNA purification cartridge. Concentrations of purified alkylated oligonucleotides were analyzed with the Thermo Scientific Nanodrop-One at 260 nm.

HPLC purification method: The mixture was diluted to a final concentration of 100 mM with pH 7.0 TEAA and purified by preparative HPLC. The collected fractions were concentrated, resuspended in H_2_O, and quantified by A_260_.

** Nanodrop - As oligonucleotide length is long enough, the change of extinction coefficient by O^6^-G acetonylation was confirmed to be negligible.

5. Analysis of Modification Site of Acetonylated Oligonucleotides

**5.1. Tandem mass spectrometry on ssODN 8a**

The reaction mixture containing **8a** was desalted by Illustra MicroSpin G-25 Columns (GE healthcare) according to the manufacturer’s instructions. The desalted oligonucleotide was mixed with 3-hydroxypicolinic acid (3-HPA) and ammonium citrate in 50% MeCN-H_2_O and loaded on MTP 384 ground steel target plate (Bruker). MALDI-TOF/TOF spectra were acquired by Ultraflex III MALDI-TOF/TOF (Bruker) mass spectrometer using the positive ion Reflectron TOF mode. Tandem mass spectrometry (MS/MS) analysis was performed using LIFT-TOF/TOF mode.

**5.2 Endonuclease digestion of acetonylated hairpin ssODN 8b**

To a aqueous solution of **8b** in EcoRI buffer was added EcoRI (Thermo Scientific^TM^, Cat.# ER0271) (final concentration: oligo (25 μM), EcoRI (1.5 U/μL), 1x EcoRI buffer; final volume (10 μL)), and incubated for 12 h at 37 ℃. The crude mixture was analyzed by HPLC-MS.

6. Primer extension assay opposite to O^6^-acetonyl G

**6.1 Introduction of single nucleotide opposite to O^6^-acetonylated G or natural G** **by DNA polymerase**

A solution of nTaq (Enzynomics, South Korea), dsODN **8h**, dNTP, and nTaq buffer (Enzynomics, Cat.# P025A, Mg^2+^ plus buffer) was incubated at 37 ℃ by using SimpliAmp thermocycler (Thermo Fisher Scientific, USA) (Final concentration: nTaq DNA polymerase (1.25 U), dsDNA (2.5 μM), dNTP (1 mM), 1x nTaq buffer; final volume: 20 μL). The mixture was quenched with 2x formamide loading buffer and analyzed by 20% denaturing PAGE (7 M urea, 20:1 = acrylamide: bisacrylamide, 1x TBE buffer) at 50 ℃ with 180 V. The gels were stained with SYBR Gold and visualized by Chemidoc (Bio-Rad).

7. Chemical Ligation of ODNs

**7.1. Double acetonylation**

The reaction was performed with several modifications from **General Procedure B**; using **12a** (50 nmol), **12b** (50 nmol), **12c** (50 nmol), water (3 μL), Mg(OAc)_2_ (2 μL, 250 mM), MES buffer (2 μL, 250 mM, pH 6.0), diazoacetone (2 μL, 400 mM), for 1 h.

After 1 h, the mixture was vigorously washed with ethyl acetate (30 μL, 3 times). Combined organic layers were discarded, and the aqueous layer was concentrated. The dried mixture from aqueous layer was used for ligation without further purification.

Derivatization of the acetonylated products by oxime ether conjugation with benzyloxyamine was performed as partial separation of HPLC peaks was observed. The crude material from the reaction was diluted with water (20 μL), extracted with ethyl acetate (30 μL x 3), and the resulting aqueous layer was concentrated. The crude material was dissolved in water, and a small portion (5 nmol of dsDNA) was diluted with water to a final volume of 4 μL. The solution was treated with benzyloxyamine (1 μL, 50 mM in DMSO), kept at r.t. for 1 h, and analyzed by HPLC-MS.

**7.2 Chemical ligation of two adjacent ODN fragments on a template**

To an aqueous solution containing **12a**, **13b** and **13c** (0.5 μL, 2 mM) suspended in water (78 μL), MES (10 μL, 50 mM, pH 6.0), and Mg(OAc)_2_ (10 μL, 50 mM) was added an aqueous solution of **5** (1.5 μL, 10 mM in H_2_O) and mixed thoroughly. The reaction mixture was kept at r.t. for 1 h. The reaction mixture was analyzed HPLC-MS and 15% denaturing PAGE (7 M urea, 20 : 1 = acrylamide : bisacrylamide) for 1 h at 180 V.

8. Single-step Synthesis of Photocaged ONs

The reaction was performed with several modifications from **General Procedure B**; **16** in DMSO (1.5 μL, 400 mM), [Rh(COD)Cl]_2_ in DMSO (0.5 μL, 10 mM), for 1 h.

For ORN-ODN hybrid duplex **7o**; water (3.75 μL), Mg(OAc)_2_ (2 μL, 250 mM), MES buffer (2 μL, 250 mM, pH 6.0), **16** in DMSO (1.25 μL, 800 mM), and [Rh(COD)Cl]_2_ in DMSO (0.5 μL, 10 mM)

**Deprotection of photocaged hairpin ssODN** **17b**: The mixture of **7b** and **17b** from the alkylation reaction (1 μL) was diluted with water (49 μL), and the solution was irradiated for 10 min at 302 nm. The reaction mixture was analyzed by HPLC-MS.

9. DNA-protein Cross-linking

**9.1 DNA-protein conjugation with T7 RNA polymerase**

To a solution of dsDNA containing O^6^-acetonyl G in HEPES buffer was added T7 RNA polymerase (Enzynomics) and incubated for 30 min at 4 ℃. The solution was treated with a stock solution of NaCNBH_3_ (Final concentration: T7 RNA polymerase (6.8 μM), dsDNA (13.5 μM), NaCNBH_3_ (0.1 M), HEPES (40 mM), Mg(OAc)_2_ (5 mM), pH 7.4; Final volume: 15 μL) and incubated at 37 ℃ for 12 h by using SimpliAmp thermocycler (Thermo Fisher Scientific). The mixture was quenched with 4x SDS loading buffer and analyzed by 5% SDS-PAGE (30 : 1 = acrylamide : bisacrylamide, 1x SDS Tris-Cl buffer) with 180 V. The gels were visualized by SYBR Gold staining (Thermo Fisher Scientific) and silver staining by using ChemiDoc (Bio-Rad).

**9.2 Preparation of *E. coli* lysate**

DH5α (Intron Biotechnology, Cat. #15063) was plated on LB-agar plates and incubated overnight. A single colony was incubated in LB medium (20 mL) at 37 ℃ until the culture reached to OD_600_ of 0.6. The cell pellet was harvested by centrifugation. The pellet was washed with 1x PBS 4 times and lysed by sonication on ice (20 x 15 s) with 15 s intervals. The resultant suspension was centrifuged (17,000 x g, 4 ℃, 20 min), pelleted, and the supernatant was used for the DNA-protein crosslinking in the lysate.

**9.3 DNA-protein conjugation with T7 RNA polymerase in *E. coli* lysate**

To a solution of dsDNA containing O^6^-acetonyl G in HEPES buffer and *E. coli* lysate was added T7 RNA polymerase (Enzynomics) and incubated for 30 min at 4 ℃. The solution was treated with a stock solution of NaCNBH_3_ (Final concentration: T7 RNA polymerase (3.4, 6.8, 13.5 μM), dsDNA (13.5 μM), NaCNBH_3_ (0.1 M), HEPES (40 mM, pH 7.4), Mg(OAc) (5 mM), 30% *E. coli* lysate, pH 7.4; Final volume: 15 μL) and incubated at 37 ℃ for 12 h by using SimpliAmp thermocycler (Thermo Fisher Scientific). The mixture was quenched with 4x SDS loading buffer and analyzed by 6% SDS-PAGE (30 : 1 = acrylamide : bisacrylamide, 1x SDS Tris-Cl buffer) with 180 V. The gels were visualized by UV (302 nm) for fluorescence (Rhodamine B), SYBR Gold staining (Thermo Fisher Scientific), and silver staining by using ChemiDoc (Bio-Rad).

**9.4. Preparation of functionalized oligonucleotides for DNA-protein cross-linking**

**9.4.1. Preparation of ssDNA functionalized with Rhodamine B**

The reaction was performed with several modifications from **General Procedure B**; using **20**, water (2.5 μL), Mg(OAc)_2_ (2 μL, 250 mM), MES buffer (2 μL, 250 mM, pH 6.0), diazoacetone (2.5 μL, 400 mM).

After 45 min, diluted with water (20 μL), vigorously washed with ethyl acetate (30 μL, 3 times). Combined organic layers were discarded, and the aqueous layer was concentrated. The dried crude material was resuspended in water (36.8 μL) and an aqueous solution of **29d** (9.2 μL, 50 mM in H_2_O) was added and mixed thoroughly. The reaction mixture was kept at r.t. for 1 h and was purified by preparative HPLC using a gradient of acetonitrile with 5 mM triethylammonium acetate (pH 7.0) as an eluent. The collected fractions were concentrated with the Thermo Scientific SPD131DDA SpeedVac Concentrator to yield pure **21** (14 nmol, 28%), which was resuspended in water and quantified by using the Thermo Scientific Nanodrop-One at 260 nm.

**9.4.2. Preparation of Rhodamine B & ketone functionalized dsDNA**

The reaction was performed in PCR tubes in a total volume of 2.8 μL. An aqueous solution of **21 (S1-RB)** (14 nmol) and S2 (14 nmol) in a PCR tube was concentrated, and the ON resuspended in water (0.6 μL) was treated with an aqueous solution of Mg(OAc)_2_ (0.6 μL, 250 mM), MES buffer (0.6 μL, 250 mM, pH 6.0), and an aqueous solution of diazoacetone (0.7 μL, 400 mM). A solution of [Rh(COD)Cl]_2_ in THF (0.3 μL, 5 mM) was added and mixed thoroughly. The reaction mixture was incubated at r.t. for 45 min and analyzed by HPLC-MS. The mixture was diluted with water (30 μL) and vigorously washed with ethyl acetate (30 μL x 3). Combined organic layers were discarded, and the aqueous layer was concentrated.

Derivatization of the acetonylated products by oxime ether conjugation with benzyloxyamine was performed as partial separation of HPLC peaks was observed. The crude material from the reaction was diluted with water (20 μL), extracted with ethyl acetate (30 μL x 3), and the resulting aqueous layer was concentrated. The crude material from aqueous layer was dissolved in water, and a small portion (5 nmol of dsDNA) was diluted with water to a final volume of 4 μL. The solution was treated with benzyloxyamine (1 μL, 50 mM in DMSO), kept at r.t. for 1 h (unless otherwise noted), and analyzed by HPLC-MS.

The rest of the crude material from aqueous layer was further purified with the Glen-Park DNA purification cartridge. Concentrations of purified alkylated oligonucleotides were analyzed with the Thermo Scientific Nanodrop-One at 260 nm to give **22a** (70% acetonylated, calculated from oxime ether conjugation). Fluorescence emission was measured by excitation at 570 nm.

10. HPLC-MS Spectra

Supplementary Figure 11 – 15. Selected reactions in Table 1

**Table 1, Entry 3**

**before reaction**

**
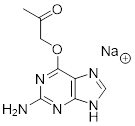
**
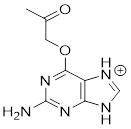

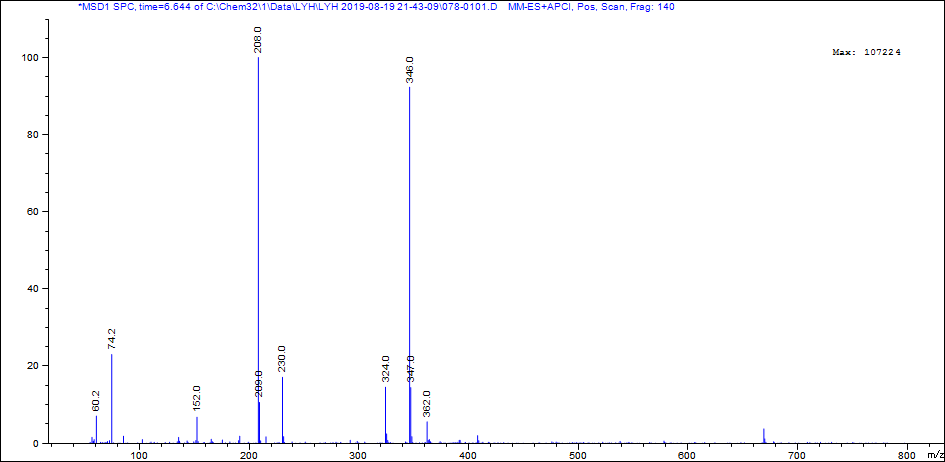

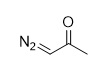


**208.0**

**346.0**

**230.0**

calcd. 230.1

found 230.0

calcd. 208.1

found 208.0

**324.0**

[M+H]^+^

calcd. 324.1

found 324.0

[M+Na]^+^

calcd. 346.1

found 346.0

**dA**

**dC**

**dC**

**dT**

**dG**

**at 30 min**

**dT**

**6**

**dG**

**dA**

**Supplementary Figure 11.** HPLC-MS analysis of Entry 3 of Table 1. Elution method (solvent A: 0.1% formic acid in H_2_O, solvent B: acetonitrile): 0/0-3/25-7/25-9/90-10/0 (time [min]/solvent B).

**Table 1, Entry 4**

**before reaction at 10 min**

**dA**


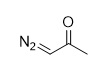


**6**

**dT**

**dC**

**dC**

**dG**

**dA**

**dT**

**Supplementary Figure 12.** HPLC-MS analysis of Entry 4 of Table 1. Elution method (solvent A: 0.1% formic acid in H_2_O, solvent B: acetonitrile): 0/0-3/25-7/25-9/90-10/0 (time [min]/solvent B).

**Table 1, Entry 5**

**before reaction o/n**

**dG**

**dA**


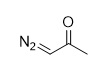

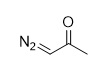


**dT**

**dG**

**dA**

**dC**

**dC**

**6**

**dT**

**Supplementary Figure 13.** HPLC-MS analysis of Entry 5 of Table 1. Elution method (solvent A: 0.1% formic acid in H_2_O, solvent B: acetonitrile): 0/0-3/25-7/25-9/90-10/0 (time [min]/solvent B).

**Table 1, Entry 6**

**before reaction o/n**


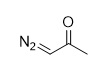

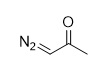


**dC**

**6**

**dG**

**dT**

**dA**

**dG**

**dC**

**dA**

**dT**

**Supplementary Figure 14.** HPLC-MS analysis of Entry 6 of Table 1. Elution method (solvent A: 0.1% formic acid in H_2_O, solvent B: acetonitrile): 0/0-3/25-7/25-9/90-10/0 (time [min]/solvent B).

**Table 1, Entry 7**

**before reaction o/n**

**dA**


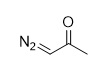

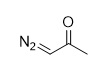


**6**

**dT**

**dG**

**dC**

**dT**

**dA**

**dG**

**dC**

**Supplementary Figure 15.** HPLC-MS analysis of Entry 7 of Table 1. Elution method (solvent A: 0.1% formic acid in H_2_O, solvent B: acetonitrile): 0/0-3/25-7/25-9/90-10/0 (time [min]/solvent B).

Supplementary Figure 16 – 19. Selected reactions in Supplementary Table 2

**Supplementary Table 2, Entry 1**

**before reaction at 1.5 h**


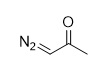


**1**

**6**

**1**

**Supplementary Figure 16.** HPLC-MS analysis of Entry 1 of Supplementary Table 2. Elution method (solvent A: 0.1% formic acid in H_2_O, solvent B: acetonitrile): 0/0-3/25-7/25-9/90-10/0 (time [min]/solvent B).

**Supplementary Table 2, Entry 2**

**before reaction at 1.5 h**


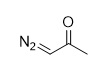


**1**

**6**

**Supplementary Figure 17.** HPLC-MS analysis of Entry 2 of Supplementary Table 2. Elution method (solvent A: 0.1% formic acid in H_2_O, solvent B: acetonitrile): 0/0-3/25-7/25-9/90-10/0 (time [min]/solvent B).

**Supplementary Table 2, Entry 3**

**before reaction at 10 min**


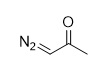


**6**

**1**

**1**

**Supplementary Figure 18.** HPLC-MS analysis of Entry 3 of Supplementary Table 2. Elution method (solvent A: 0.1% formic acid in H_2_O, solvent B: acetonitrile): 0/0-3/25-7/25-9/90-10/0 (time [min]/solvent B).

**Supplementary Table 2, Entry 4**

**before reaction at 10 min**


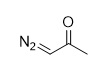


**1**

**1**

**6**

**Supplementary Figure 19.** HPLC-MS analysis of Entry 4 of Supplementary Table 2. Elution method (solvent A: 0.1% formic acid in H_2_O, solvent B: acetonitrile): 0/0-3/25-7/25-9/90-10/0 (time [min]/solvent B).

Supplementary Figure 20. Comparison of the reactivity between deoxyguanosine and deoxyinosine


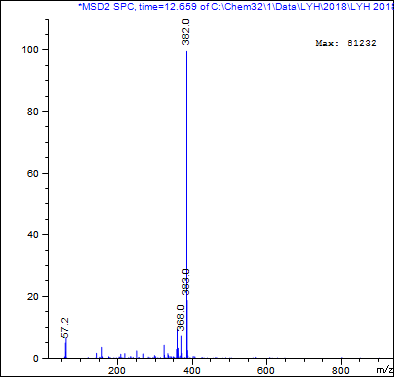
**before reaction**

**382.0**

**6**

[M+CH_3_CO_2_]^-^

calcd. 382.1

found 382.0


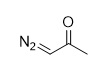


**23**

**1**


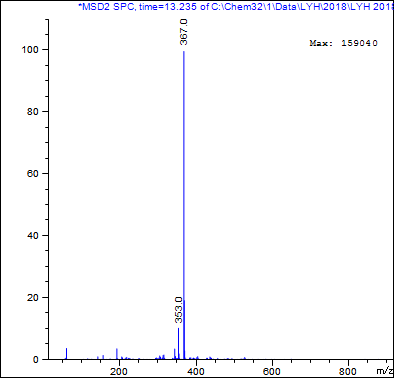
**at 30 min**

**367.0**

**24**

[M+CH_3_CO_2_]^-^

calcd. 367.1

found 367.0

**6**

**24**

**23**

**1**

calcd. 230.1

found 230.0

**Supplementary Figure 20.** HPLC-MS analysis of comparison of the reactivity between deoxyguanosine and deoxyinosine. Elution method (solvent A: 5 mM TEAA, solvent B: acetonitrile): 0/0-9/10-10/90-14.5/90-15.5/0 (time [min]/solvent B).

Supplementary Figure 21. Modification of 7-deaza-dG with Rh(I)-catalysis

**before reaction at 10 min**


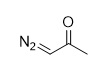


**31**

**31**

**Supplementary Figure 21.** HPLC-MS analysis of the reaction of 7-deaza-dG with Rh(I)-catalysis. Elution method (solvent A: 0.1% formic acid in H_2_O, solvent B: acetonitrile): 0/0-4/20-5/90-9/90-10/0 (time [min]/solvent B).

Supplementary Figure 22. Modification of N^7^-methyl-2′-deoxyguanosine with Rh(I)-catalysis

**
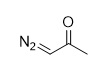
 before reaction at 1.5 h**

**1a**

**
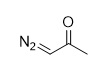
**

**1a**

**6a**

**1a**


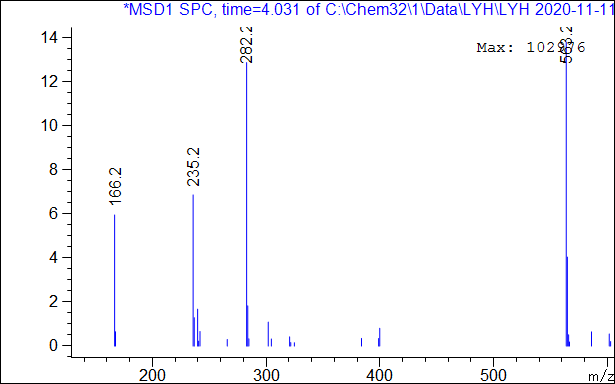


*****

**M^+^**

Calcd: 282.1

Found: 282.2

**6a**


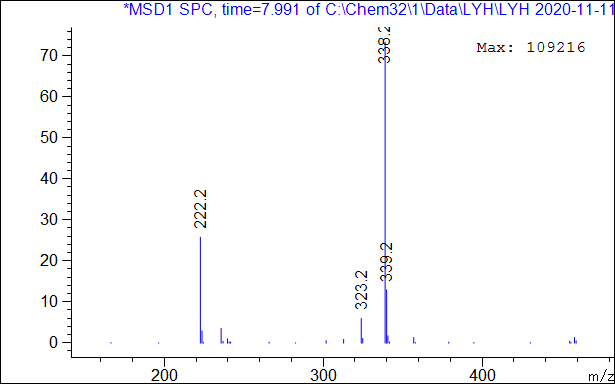


*****

**M^+^**

Calcd: 338.2

Found: 338.2

**Supplementary Figure 22.** HPLC-MS analysis of the reaction of N^7^-methyl-2′-deoxyguanosine with Rh(I)-catalysis. Asterisk(*) for found nucleobase fragment. Elution method (solvent A: 5 mM TEAA, solvent B: acetonitrile): 0/0-7/10-8/90-11/90-12/0 (time [min]/solvent B).

Supplementary Figure 23. Modification of c-di-GMP with Rh(I)-catalysis

**before reaction at 1 h**

**32**


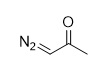


**34**

**33**

**32**

**33**


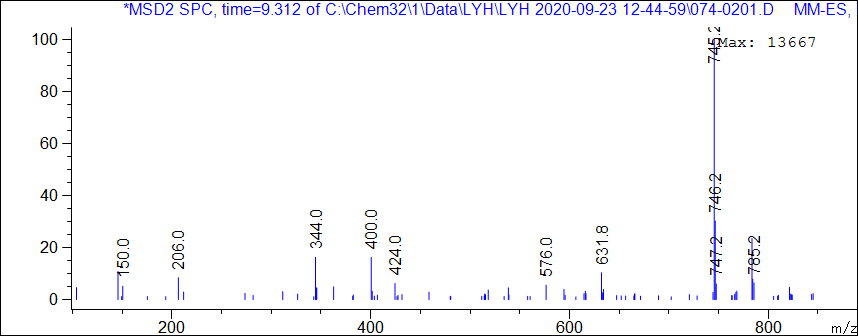


*****

*****

*****

*****

[M-H]^-^

calcd. 745.1

found 745.2

**34**


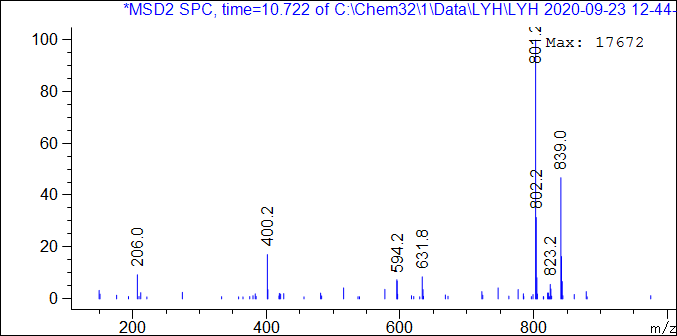


*****

*****

[M-H]^-^

calcd. 801.1

found 801.2

[M+K-2H]^-^

calcd. 839.1

found 839.0

**Supplementary Figure 23.** HPLC-MS analysis of the reaction of c-di-GMP with Rh(I)-catalysis. Asterisk(*) for found fragments. Elution method (solvent A: 5 mM TEAA, solvent B: acetonitrile): 0/0-14/20-15/90-18/90-20/0 (time [min]/solvent B).

Supplementary Figure 24 – 27. Selected reactions in Supplementary Table 3

**before reaction 7a**

**[M-2H]^2-^**

**585.6**


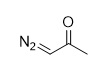

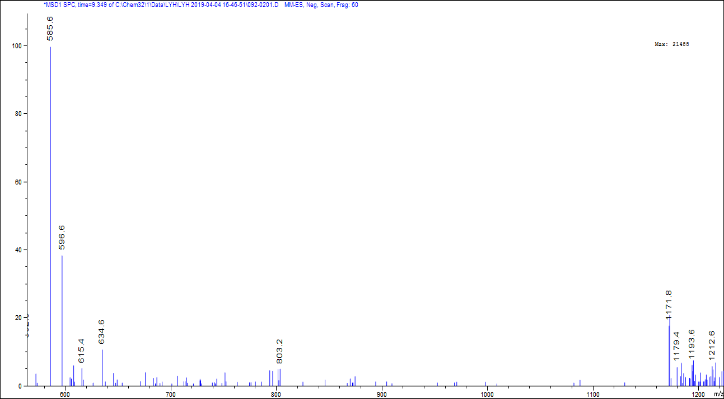


**7a**

**[M-H]^-^**

**1171.8**

**at 30 min 8a**


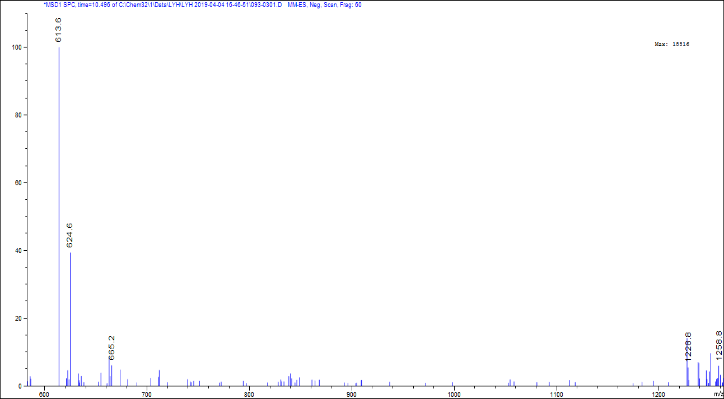


**[M-2H]^2-^**

**613.6**

**[M-H]^-^**

**1228.8**

**8a**

**7a**

**Calculated and found MS data**

| calculated mass | | calculated m/z | |
| --- | --- | --- | --- |
| sequence | exact mass | [M-H]^-^ | [M-2H]^2-^ |
| **7a** | 1173.2 | 1172.2 | 585.6 |
| **8a** | 1229.3 | 1228.2 | 613.6 |
| **before reaction** | | found m/z | |
| sequence | LC RT^a^ | [M-H]^-^ | [M-2H]^2-^ |
| **7a** | 9.268 | 1171.8 | 585.6 |
| **at 30 min** | | found m/z | |
| sequence | LC RT | [M-H]^-^ | [M-2H]^2-^ |
| **8a** | 10.437 | 1228.8 | 613.6 |

**Supplementary Figure 24.** HPLC-MS analysis of acetonylation of **7a.** Elution method (solvent A: 5 mM TEAA, solvent B: acetonitrile): 0/0-14/20-15/90-19/90-20/0 (time [min]/Solvent B%) with flowrate 0.3 mL/min. ^a^RT = retention time.

**before reaction 7r**

**[M-3H]^3-^**

**1002.8**


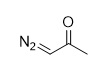

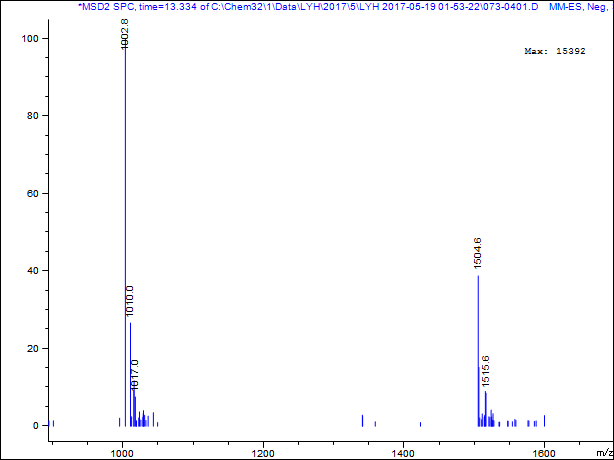


**[M-2H]^2-^**

**1504.8**

**7r**


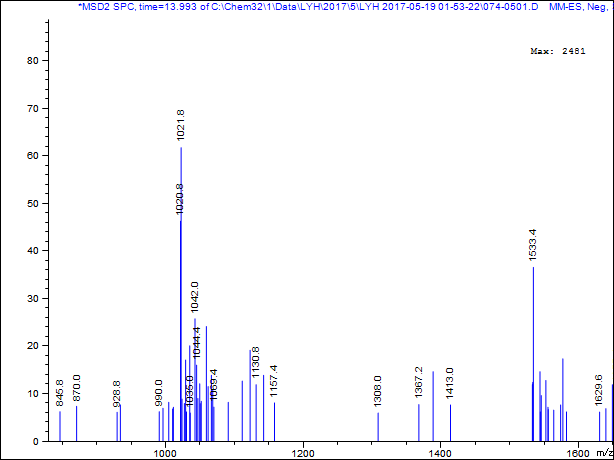

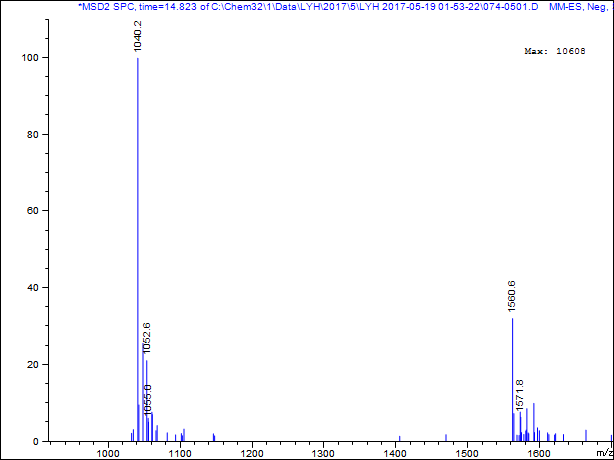

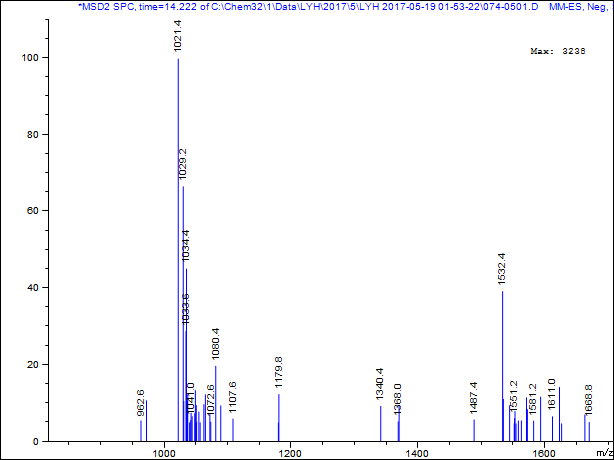
**at 1 h**  **S1-act** or **S1-act‘** (13.993 & 14.222 min) **8r**

**[M-3H]^3-^**

**1040.2**

**[M-3H]^3-^**

**1021.4**

**[M-3H]^3-^**

**1021.8**

**[M-2H]^2-^**

**1560.6**

**[M-2H]^2-^**

**1533.4**

**[M-2H]^2-^**

**1532.4**

**8r**

**S1-act or S1-act’**

**Calculated and found MS data**

| calculated mass | | calculated m/z | |
| --- | --- | --- | --- |
| sequence | exact mass | [M-2H]^2-^ | [M-3H]^3-^ |
| **7r** | 3010.6 | 1504.8 | 1002.8 |
| S1-act/ S1-act’ | 3066.6 | 1532.8 | 1021.5 |
| **8r** | 3122.6 | 1560.8 | 1040.2 |
| **before reaction** | | found m/z | |
| sequence | LC RT^a^ | [M-2H]^2-^ | [M-3H]^3-^ |
| **7r** | 13.286 | 1504.6 | 1002.8 |
| **at 1 h** | | found m/z | |
| sequence | LC RT | [M-2H]^2-^ | [M-3H]^3-^ |
| S1-act/ S1-act’ | 13.893, 14.146 | 1533.4, 1532.4 | 1021.8, 1021.4 |
| **8r** | 14.766 | 1560.6 | 1040.2 |

**Supplementary Figure 25.** HPLC-MS analysis of acetonylation of **7r.** Elution method (solvent A: 5 mM TEAA, solvent B: acetonitrile): 0/0-18/15-19/90-25/90-26/0 (time [min]/Solvent B%) with flowrate 0.3 mL/min. ^a^RT = retention time.

**before reaction 7s**

**[M-3H]^3-^**

**1685.8**

**[M-4H]^4-^**

**1264.0**

**7s**


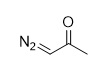

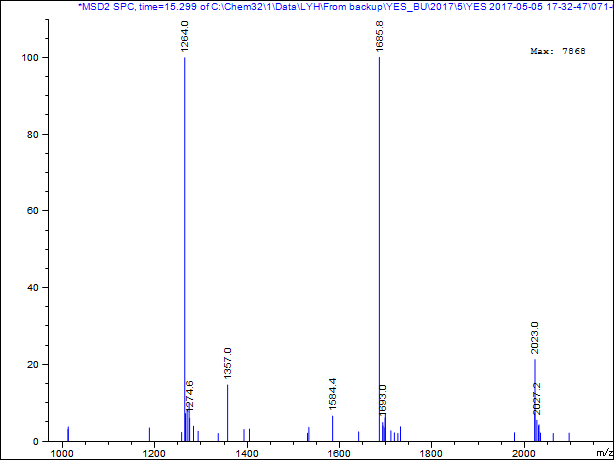


**[2M-5H]^5-^**

**2023.0**

**at 30 min 7s**

**[M-4H]^4-^**

**1264.2**


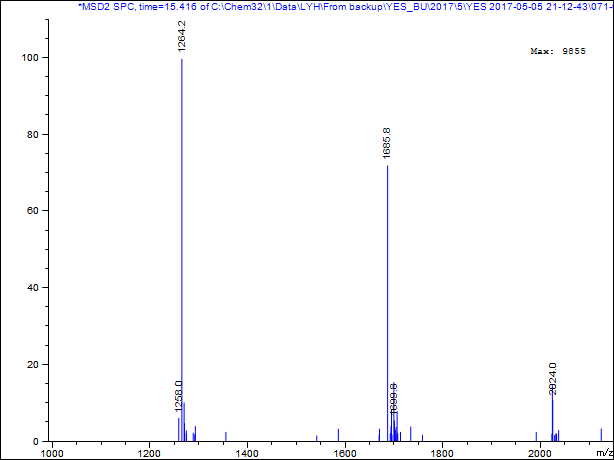


**[M-3H]^3-^**

**1685.8**

**[2M-5H]^5-^**

**2024.0**

**7s**

**Calculated and found MS data**

| calculated mass | | calculated m/z | | |
| --- | --- | --- | --- | --- |
| sequence | MW/EM | [M-3H]^3-^ | [M-4H]^4-^ | [2M-5H]^5-^ |
| **7s** | 5058.9 | 1685.6 | 1264.0 | 2023.1 |
| **before reaction** | | found m/z | |  |
| sequence | LC RT^a^ | [M-3H]^3-^ | [M-4H]^4-^ | [2M-5H]^5-^ |
| **7s** | 15.233 | 1685.8 | 1264.0 | 2023.0 |
| **at 30 min** | | found m/z | |  |
| sequence | LC RT | [M-3H]^3-^ | [M-4H]^4-^ | [2M-5H]^5-^ |
| **7s** | 15.346 | 1685.8 | 1264.2 | 2024.0 |

**Supplementary Figure 26.** HPLC-MS analysis of acetonylation of **7s.** Elution method (solvent A: 5 mM TEAA, solvent B: acetonitrile): 0/0-18/15-19/90-25/90-26/0 (time [min]/Solvent B%) with flowrate 0.3 mL/min. ^a^RT = retention time.

**before reaction 7t**

**[M-4H+Na]^3-^**

**1033.6**


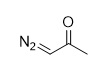

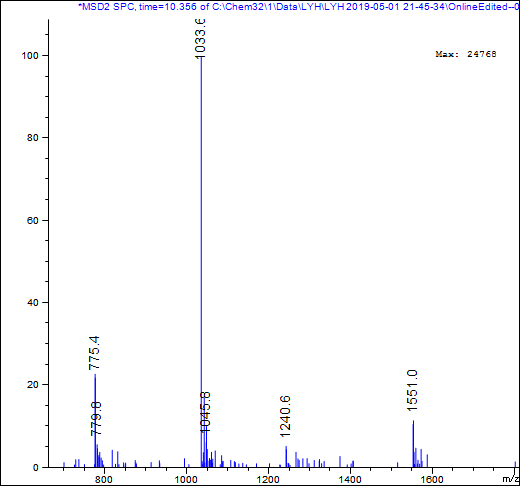


**[M-3H+Na]^2-^**

**1551.0**

**[M-5H+Na]^4-^**

**775.4**

**7t**

**at 30 min**  **8t**

**[M-4H+Na]^3-^**

**1052.2**


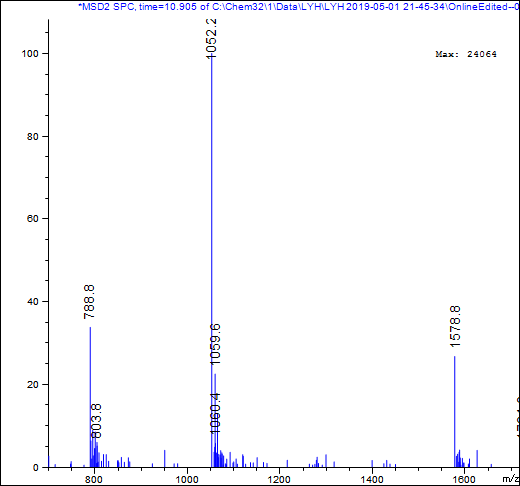


**[M-5H+Na]^4-^**

**788.8**

**[M-3H+Na]^2-^**

**1578.8**

**8t**

**7t**

**Calculated and found MS data**

| calculated mass | | calculated m/z | | |
| --- | --- | --- | --- | --- |
| sequence | exact mass | [M-3H+Na^+^]^2-^ | [M-4H+Na^+^]^3-^ | [M-5H+Na^+^]^4-^ |
| **7t** | 3080.4 | 1550.7 | 1033.5 | 774.8 |
| **8t** | 3136.4 | 1578.7 | 1052.1 | 788.9 |
| **before reaction** | | found m/z | | |
| sequence | LC RT^a^ | [M-3H+Na^+^]^2-^ | [M-4H+Na^+^]^3-^ | [M-5H+Na^+^]^4-^ |
| **7t** | 10.313 | 1551.0 | 1033.6 | 775.4 |
| **at 30 min** | | found m/z | | |
| sequence | LC RT | [M-3H+Na^+^]^2-^ | [M-4H+Na^+^]^3-^ | [M-5H+Na^+^]^4-^ |
| **8t** | 10.831 | 1578.8 | 1052.2 | 788.8 |

**Supplementary Figure 27.** HPLC-MS analysis of acetonylation of **7t**. Elution method (solvent A: 5 mM TEAA, solvent B: acetonitrile): 0/0-20/20-21/90-25/90-26/0 (time [min]/Solvent B%) with flowrate 0.3 mL/min. ^a^RT = retention time.

Supplementary Figure 28 – 43. Selected reactions in Table 2

**before reaction 7b**

**[M-8H]^8-^**

**879.2**


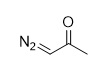

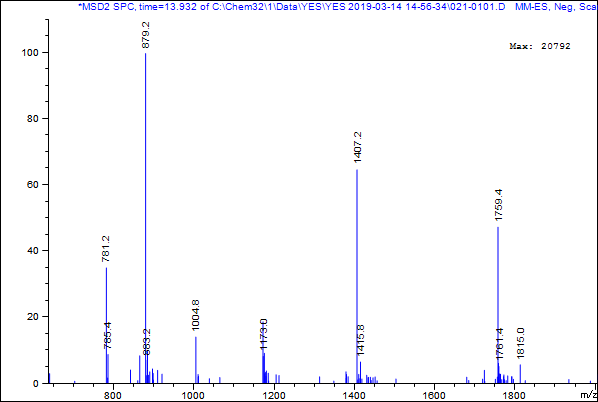


**7b**

**[M-7H]^7-^**

**1004.8**

**[M-9H]^9-^**

**781.2**

**[M-4H]^4-^**

**1759.4**

**[M-5H]^5-^**

**1407.2**

**[M-6H]^6-^**

**1173.0**

**at 30 min 8b**

**[M-7H]^7-^**

**1012.6**

**[M-8H]^8-^**

**886.0**

**[M-5H]^5-^**

**1418.4**

**[M-9H]^9-^**

**787.6**

**[M-4H]^4-^**

**1773.2**

**[M-6H]^6-^**

**1182.6**


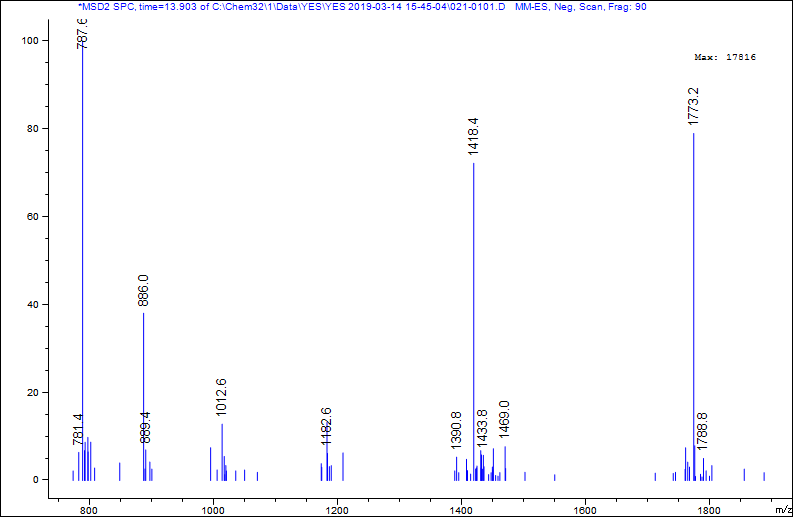


**8b**

**Calculated and found MS data**

| calculated mass | | calculated m/z | | | | | |
| --- | --- | --- | --- | --- | --- | --- | --- |
| sequence | exact mass | [M-4H]^4-^ | [M-5H]^5-^ | [M-6H]^6-^ | [M-7H]^7-^ | [M-8H]^8-^ | [M-9H]^9-^ |
| **7b** | 7120.2 | 1759.5 | 1407.4 | 1172.7 | 1005.0 | 879.3 | 781.5 |
| **8b** | 7176.2 | 1773.6 | 1418.6 | 1182.0 | 1013.0 | 886.2 | 787.7 |
| **before reaction** | | found m/z | | | | | |
| sequence | LC RT^a^ | [M-4H]^4-^ | [M-5H]^5-^ | [M-6H]^6-^ | [M-7H]^7-^ | [M-8H]^8-^ | [M-9H]^9-^ |
| **7b** | 18.635 | 1759.4 | 1407.2 | 1173.0 | 1004.8 | 879.2 | 781.2 |
| **at 30 min** | | found m/z | | | | | |
| sequence | LC RT | [M-4H]^4-^ | [M-5H]^5-^ | [M-6H]^6-^ | [M-7H]^7-^ | [M-8H]^8-^ | [M-9H]^9-^ |
| **8b** | 18.975 | 1773.2 | 1418.4 | 1182.6 | 1012.6 | 886.0 | 787.6 |

**Supplementary Figure 28.** HPLC-MS analysis of acetonylation of **7b.** Elution method (solvent A: 20 mM TEAA, solvent B: acetonitrile): 0/0-33/15-35/90-38/90-40/0 (time [min]/Solvent B%) with flowrate 0.3 mL/min. ^a^RT = retention time.

**before reaction**  **7c**

**7c**

**[M-4H]^4-^**

**1372.6**


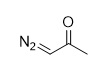

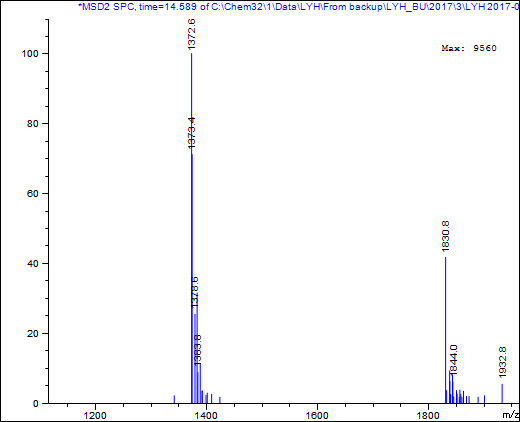


**[M-3H]^3-^**

**1830.8**

**at 30 min 8c**

**[M-4H]^4-^**

**1387.0**


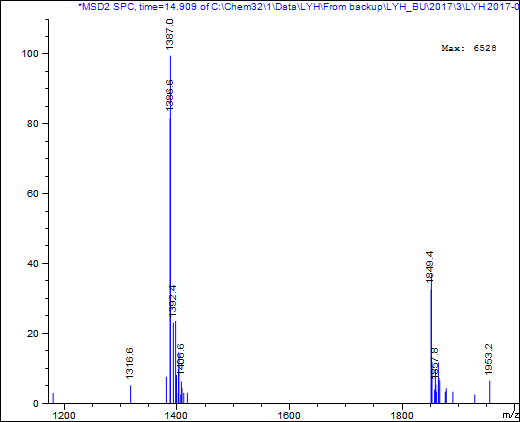


**7c**

**8c**

**[M-3H]^3-^**

**1849.4**

**Calculated and found MS data**

| calculated mass | | calculated m/z | |
| --- | --- | --- | --- |
| sequence | exact mass | [M-3H]^3-^ | [M-4H]^4-^ |
| **7c** | 5493.9 | 1830.6 | 1372.7 |
| **8c** | 5550.0 | 1849.3 | 1386.7 |
| **before reaction** | | found m/z | |
| sequence | LC RT^a^ | [M-3H]^3-^ | [M-4H]^4-^ |
| **7c** | 14.490 | 1830.8 | 1372.6 |
| **at 30 min** | | found m/z | |
| sequence | LC RT | [M-3H]^3-^ | [M-4H]^4-^ |
| **8c** | 14.790 | 1849.4 | 1387.0 |

**Supplementary Figure 29.** HPLC-MS analysis of acetonylation of **7c.** Elution method (solvent A: 5 mM TEAA, solvent B: acetonitrile): 0/0-18/15-19/90-22/90-23/0 (time [min]/Solvent B%) with flowrate 0.3 mL/min. ^a^RT = retention time.

**before reaction 7d**

**[M-4H]^4-^**

**1605.0**


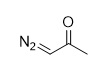

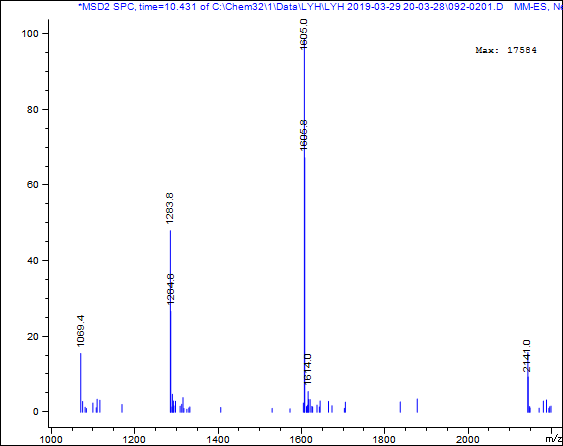


**7d**

**[M-6H]^6-^**

**1069.4**

**[M-3H]^3-^**

**2141.0**

**[M-5H]^5-^**

**1283.8**

**at 30 min 8d**

**[M-4H]^4-^**

**1619.2**


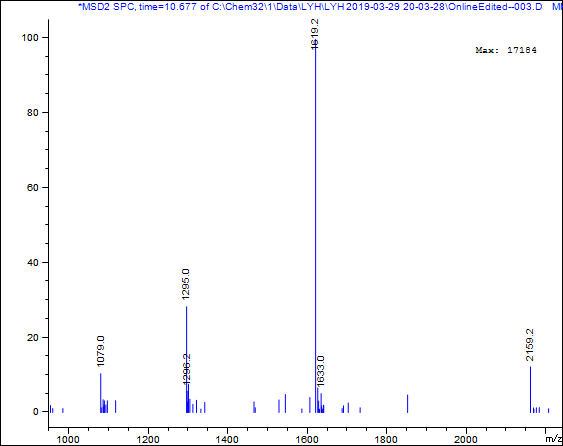


**8d**

**7d**

**[M-5H]^5-^**

**1295.0**

**[M-6H]^6-^**

**1079.0**

**[M-3H]^3-^**

**2159.2**

**Calculated and found MS data**

| calculated mass | | calculated m/z | | | |
| --- | --- | --- | --- | --- | --- |
| sequence | exact mass | [M-3H]^3-^ | [M-4H]^4-^ | [M-5H]^5-^ | [M-6H]^6-^ |
| **7d** | 6427.2/6424.1 | 2141.0 | 1605.5 | 1284.2 | 1070.0 |
| **8d** | 6483.3/6480.1 | 2159.7 | 1619.5 | 1295.4 | 1079.3 |
| **before reaction** | | found m/z | | | |
| sequence | LC RT^a^ | [M-3H]^3-^ | [M-4H]^4-^ | [M-5H]^5-^ | [M-6H]^6-^ |
| **7d** | 10.358 | 2141.0 | 1605.0 | 1283.8 | 1069.4 |
| **at 30 min** | | found m/z | | | |
| sequence | LC RT | [M-3H]^3-^ | [M-4H]^4-^ | [M-5H]^5-^ | [M-6H]^6-^ |
| **8d** | 10.579 | 2159.2 | 1619.2 | 1295.0 | 1079.0 |

**Supplementary Figure 30.** HPLC-MS analysis of acetonylation of **7d.** Elution method (solvent A: 5 mM TEAA, solvent B: acetonitrile): 0/0-14/20-15/90-19/90-20/0 (time [min]/Solvent B%) with flowrate 0.3 mL/min. ^a^RT = retention time.

**before reaction 7e**

**7e**

**[M-4H]^4-^**

**1533.4**


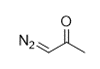

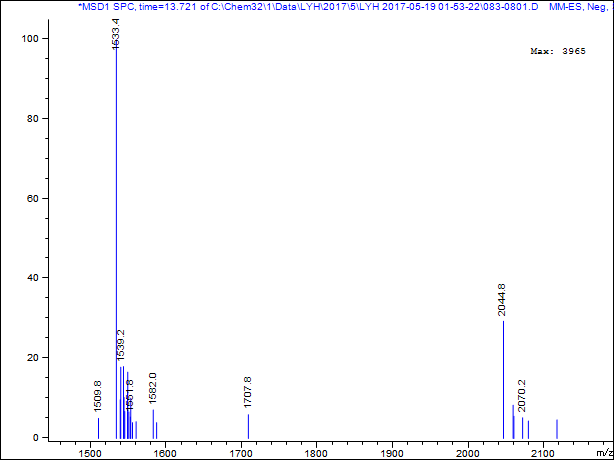


**[M-3H]^3-^**

**2044.8**

**at 30 min**  **S1-act or S1-act’** (13.878 and 14.050 min) **8e**

**[M-4H]^4-^**

**1547.2**

**S1-act**

**or S1-act’**

**[M-4H]^4-^**

**1547.8**


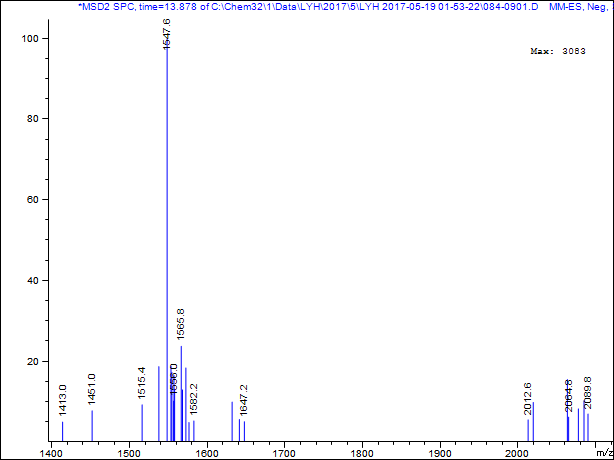

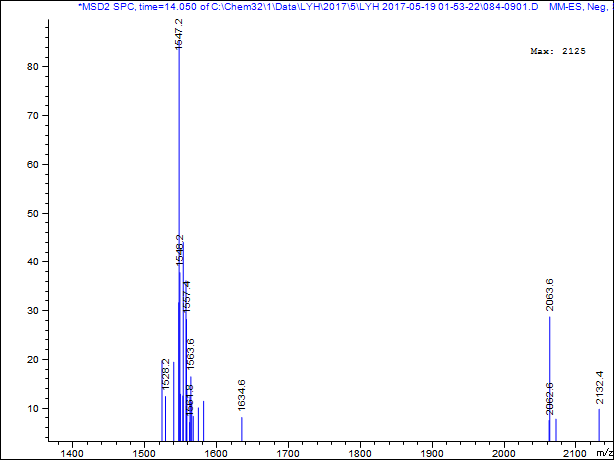

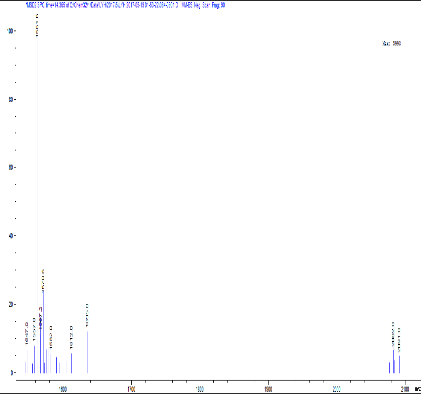


**[M-3H]^3-^**

**2063.6**

**[M-3H]^3-^**

**2064.8**

**[M-3H]^3-^**

**2082.0**

**8e**

**7e**

**[M-4H]^4-^**

**1561.6**

**Calculated and found MS data**

| calculated mass | | calculated m/z | |
| --- | --- | --- | --- |
| sequence | exact mass | [M-3H]^3-^ | [M-4H]^4-^ |
| **7e** | 6136.0 | 2045.0 | 1533.5 |
| S1-act S1-act’ | 6192.1 | 2063.7 | 1547.5 |
| **8e** | 6248.1 | 2082.4 | 1561.5 |
| **before reaction** | | found m/z | |
| sequence | LC RT^a^ | [M-3H]^3-^ | [M-4H]^4-^ |
| **7e** | 13.557 | 2044.8 | 1533.4 |
| **at 30 min** | | found m/z | |
| sequence | LC RT | [M-3H]^3-^ | [M-4H]^4-^ |
| S1-act S1-act’ | 13.782 14.005 | 2064.8, 2063.6 | 1547.6, 1547.2 |
| **8e** | 14.265 | 2082.0 | 1561.6 |

**Supplementary Figure 31.** HPLC-MS analysis of acetonylation of **7e.** Elution method (solvent A: 5 mM TEAA, solvent B: acetonitrile): 0/0-18/15-19/90-25/90-26/0 (time [min]/Solvent B%) with flowrate 0.3 mL/min. ^a^RT = retention time.

**before reaction**

**S2**


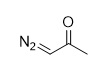


**at 30 min**

**S1**

**S1**

**S1-act**

**S2-act**

**S2**

**at 30 min**

**before reaction**

**S1**  **S1-act**

**[M-4H]^4-^**

**1515.0**

**[M-4H]^4-^**

**1501.0**


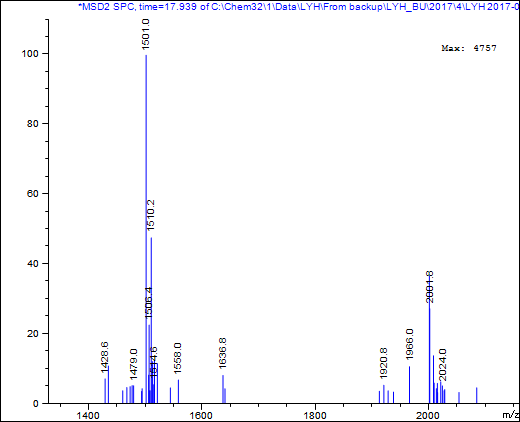

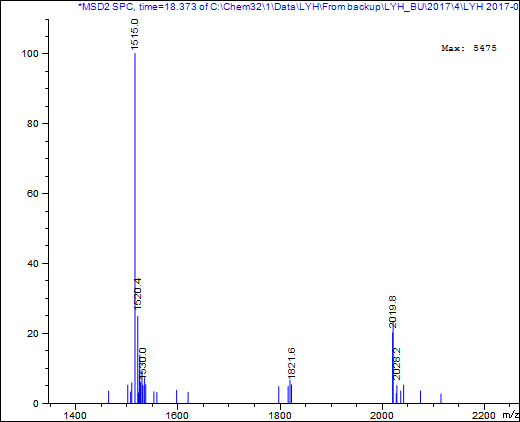


**[M-3H]^3-^**

**2019.8**

**[M-3H]^3-^**

**2001.6**

**S2** **S2**

**[M-4H]^4-^**

**1325.6**

**[M-3H]^3-^**

**1768.0**


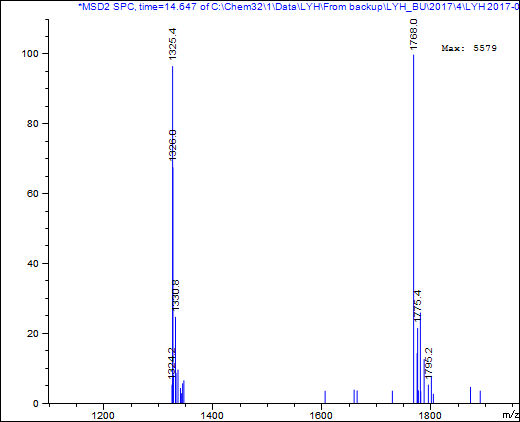

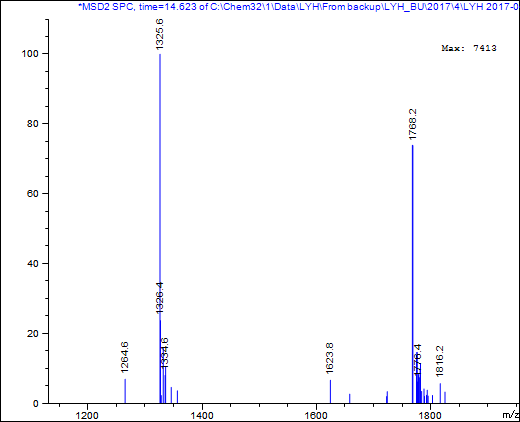


**[M-3H]^3-^**

**1768.2**

**[M-4H]^4-^**

**1325.4**

**Calculated and found MS data**

| calculated mass | | calculated m/z | |
| --- | --- | --- | --- |
| sequence | exact mass | [M-3H]^3-^ | [M-4H]^4-^ |
| **S1** | 6006.0 | 2001.7 | 1501.0 |
| **S2** | 5304.9 | 1767.6 | 1325.5 |
| **S1-act** | 6062.0 | 2020.3 | 1515.0 |
| **before reaction** | | found m/z | |
| sequence | LC RT^a^ | [M-3H]^3-^ | [M-4H]^4-^ |
| **S1** | 17.912 | 2001.6 | 1501.0 |
| **S2** | 14.612 | 1768.0 | 1325.4 |
| **at 30 min** | | found m/z | |
| sequence | LC RT | [M-3H]^3-^ | [M-4H]^4-^ |
| **S1-act** | 18.315 | 2019.8 | 1515.0 |
| **S2** | 14.575 | 1768.2 | 1325.6 |

**Supplementary Figure 32.** HPLC-MS analysis of acetonylation of **7f.** Elution method (solvent A: 5 mM TEAA, solvent B: acetonitrile): 0/0-18/15-19/90-25/90-26/0 (time [min]/Solvent B%) with flowrate 0.3 mL/min. ^a^RT = retention time.

**S1**

**S2**

**S2**

**S1-act**

**S1**

**at 30 min**

**before reaction**

**at 2 h**

**before reaction**

**S1** **S1-act**

**[M-4H]^4-^1244.4**

**[M-4H]^4-^1230.6**


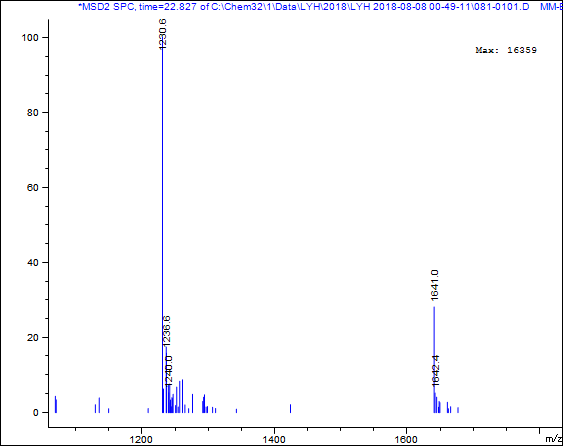

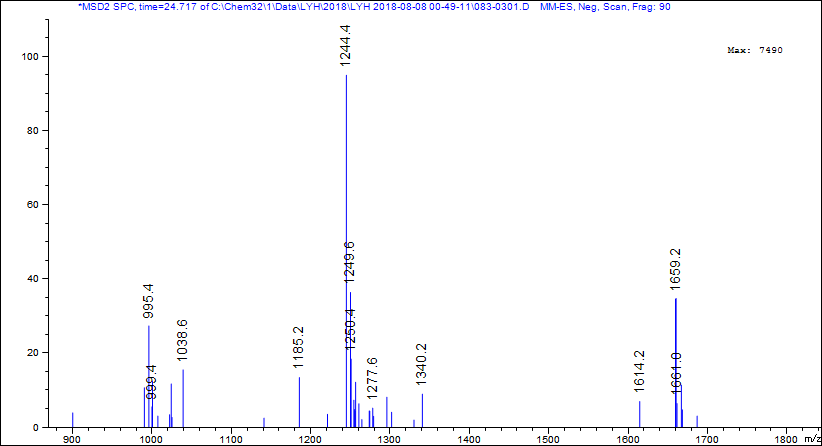


**[M-3H]^3-^1659.2**

**[M-3H]^3-^1641.0**

**S2 S2**

**[M-4H]^4-^1134.8**

**[M-4H]^4-^1134.8**


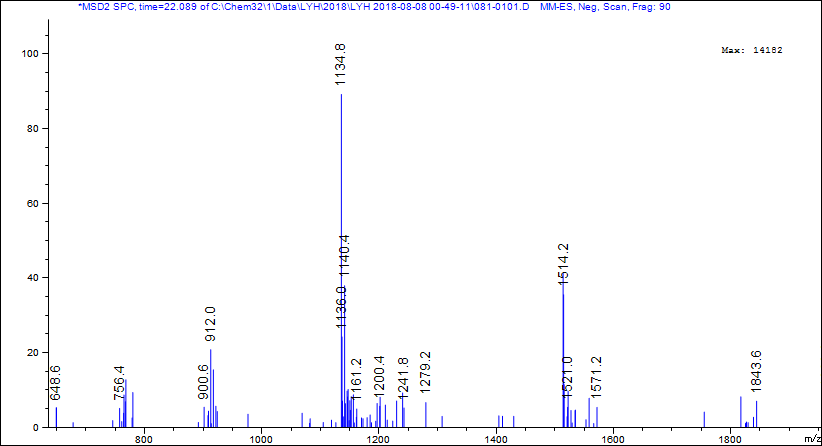

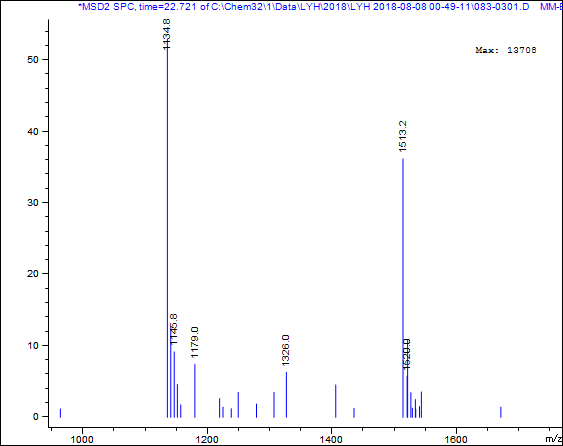


**[M-3H]^3-^1513.2**

**[M-3H]^3-^1514.2**

**Calculated and found MS data**

| calculated mass | | calculated m/z | |
| --- | --- | --- | --- |
| sequence | exact mass | [M-3H]^3-^ | [M-4H]^4-^ |
| **S1** | 4924.8 | 1640.9 | 1230.5 |
| **S2** | 4542.8 | 1513.6 | 1134.9 |
| **S1-act** | 4980.9 | 1659.6 | 1244.5 |
| **before reaction** | | found m/z | |
| sequence | LC RT^a^ | [M-3H]^3-^ | [M-4H]^4-^ |
| **S1** | 22.811 | 1641.0 | 1230.4 |
| **S2** | 22.078 | 1514.2 | 1134.8 |
| **at 2 h** | | found m/z | |
| sequence | LC RT | [M-3H]^3-^ | [M-4H]^4-^ |
| **S1-act** | 24.640 | 1659.2 | 1244.4 |
| **S2** | 22.580 | 1513.2 | 1134.8 |

**Supplementary Figure 33.** HPLC-MS analysis of acetonylation of **7g.** Elution method (solvent A: 5 mM TEAA, solvent B: acetonitrile): 0/0-33/15-34/90-38/90-40/0 (time [min]/Solvent B%) with flowrate 0.3 mL/min. ^a^RT = retention time.

**before reaction**


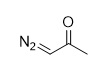

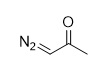


**S1**

**S2**

**at 30 min**

**S2**

**S2-act**

**S1**

**at 30 min**

**before reaction**

**S1** **S1**

**[M-7H]^7-^**

**746.4**


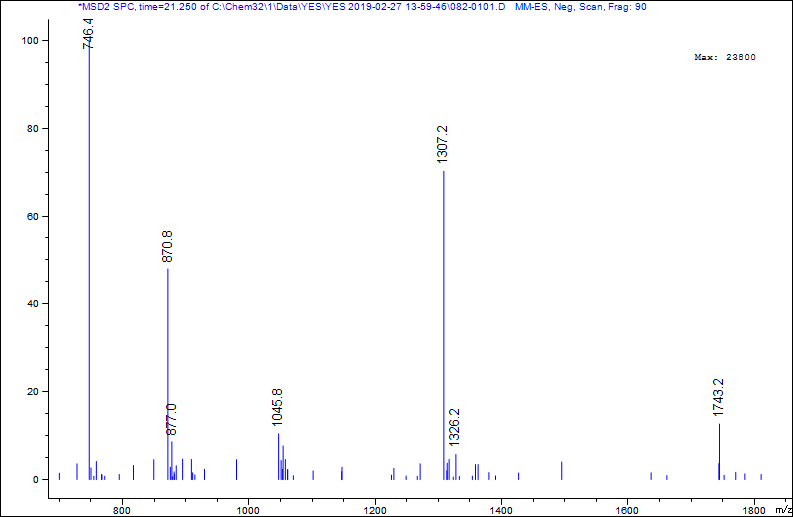


**[M-6H]^6-^**

**871.0**

**[M-4H]^4-^**

**1307.0**

**[M-3H]^3-^**

**1742.4**

**[M-5H]^5-^**

**1045.2**

**[M-7H]^7-^**

**746.4**

**[M-4H]^4-^**

**1307.2**

**[M-6H]^6-^**

**870.8**

**[M-5H]^5-^**

**1045.8**

**[M-3H]^3-^**

**1743.2**

**S2 S2-act**

**[M-11H]^11-^**

**745.2**

**[M-9H]^9-^**

**917.4**

**[M-4H]^4-^**

**2065.2**

**[M-5H]^5-^**

**1651.8**

**[M-11H]^11-^**

**750.2**

**[M-10H]^10-^**

**825.4**

**[M-6H]^6-^**

**1376.4**

**[M-7H]^7-^**

**1179.2**

**[M-8H]^8-^**

**1031.8**

**[M-8H]^8-^**

**1025.4**

**[M-7H]^7-^**

**1171.4**

**[M-6H]^6-^**

**1367.0**

**[M-4H]^4-^**

**2051.6**

**[M-5H]^5-^**

**1641.2**

**[M-10H]^10-^**

**819.8**

**[M-9H]^9-^**

**911.0**

**Calculated and found MS data**

| calculated mass | | calculated m/z | | | | |
| --- | --- | --- | --- | --- | --- | --- |
| sequence | exact mass | [M-3H]^3-^ | [M-4H]^4-^ | [M-5H]^5-^ | [M-6H]^6-^ | [M-7H]^7-^ |
| **S1** | 5231.9 | 1743.3 | 1307.2 | 1045.6 | 871.1 | 746.6 |
| sequence | exact mass |  |  |  |  |  |
| **S2** | 8208.4 |  | [M-4H]^4-^ | [M-5H]^5-^ | [M-6H]^6-^ | [M-7H]^7-^ |
|  |  |  | 2051.6 | 1641.1 | 1367.4 | 1171.9 |
|  |  |  | [M-8H]^8-^ | [M-9H]^9-^ | [M-10H]^10-^ | [M-11H]^11-^ |
|  |  |  | 1025.3 | 911.3 | 820.0 | 745.4 |
| **S2-act** | 8264.4 |  | [M-4H]^4-^ | [M-5H]^5-^ | [M-6H]^6-^ | [M-7H]^7-^ |
|  |  |  | 2065.6 | 1652.3 | 1376.7 | 1179.9 |
|  |  |  | [M-8H]^8-^ | [M-9H]^9-^ | [M-10H]^10-^ | [M-11H]^11-^ |
|  |  |  | 1032.3 | 917.5 | 825.6 | 750.5 |
| **before reaction** | | found m/z | | | | |
| sequence | LC RT^a^ | [M-3H]^3-^ | [M-4H]^4-^ | [M-5H]^5-^ | [M-6H]^6-^ | [M-7H]^7-^ |
| **S1** | 21.172 | 1743.2 | 1307.2 | 1045.8 | 870.8 | 746.4 |
| **S2** | 24.152 |  | [M-4H]^4-^ | [M-5H]^5-^ | [M-6H]^6-^ | [M-7H]^7-^ |
|  |  |  | 2051.6 | 1641.2 | 1367.0 | 1171.4 |
|  |  |  | [M-8H]^8-^ | [M-9H]^9-^ | [M-10H]^10-^ | [M-11H]^11-^ |
|  |  |  | 1025.4 | 911.0 | 819.4 | 745.2 |
| **at 30 min** | | found m/z | | | | |
| sequence | LC RT | [M-3H]^3-^ | [M-4H]^4-^ | [M-5H]^5-^ | [M-6H]^6-^ | [M-7H]^7-^ |
| **S1** | 21.094 | 1742.8 | 1307.0 | 1045.2 | 870.8 | 746.4 |
| **S2-act** | 24.847 |  | [M-4H]^4-^ | [M-5H]^5-^ | [M-6H]^6-^ | [M-7H]^7-^ |
|  |  |  | 2065.2 | 1651.8 | 1376.4 | 1179.2 |
|  |  |  | [M-8H]^8-^ | [M-9H]^9-^ | [M-10H]^10-^ | [M-11H]^11-^ |
|  |  |  | 1031.8 | 917.4 | 825.4 | 750.2 |

**Supplementary Figure 34.** HPLC-MS analysis of acetonylation of **7h.** Elution method (solvent A: 5 mM TEAA, solvent B: acetonitrile): 0/0-33/15-34/90-38/90-40/0 (time [min]/Solvent B%) with flowrate 0.3 mL/min. ^a^RT = retention time.

**[M-4H]^4-^**

**1502.6**

**before reaction 7i**

**[M-5H]^5-^**

**1202.0**

**[M-3H]^3-^**

**2004.6**

**7i**

**[M-4H]^5-^**

**1516.8**

**at 30 min 8i**

**[M-5H]^6-^**

**1212.2**

**[M-3H]^3-^**

**2022.0**

**7i**

**8i**

**Calculated and found MS data**

| calculated mass | | calculated m/z | | |
| --- | --- | --- | --- | --- |
| sequence | exact mass | [M-3H]^3-^ | [M-4H]^4-^ | [M-5H]^5-^ |
| **7i** | 6012.8 | 2003.6 | 1502.4 | 1201.8 |
| **8i** | 6068.8 | 2022.3 | 1516.5 | 1213.0 |
| **before reaction** | | found m/z | | |
| sequence | LC RT^a^ | [M-3H]^3-^ | [M-4H]^4-^ | [M-5H]^5-^ |
| **7i** | 13.069 | 2004.6 | 1502.6 | 1202.0 |
| **at 30 min** | | found m/z | | |
| sequence | LC RT | [M-3H]^3-^ | [M-4H]^4-^ | [M-5H]^5-^ |
| **8i** | 13.546 | 2022.0 | 1516.8 | 1212.2 |

**Supplementary Figure 35.** HPLC-MS analysis of acetonylation of **7i.** Elution method (solvent A: 5 mM TEAA, solvent B: acetonitrile): 0/0-34/20-35/90-38/90-40/0 (time [min]/Solvent B%) with flowrate 0.3 mL/min. ^a^RT = retention time.

**[M-4H]^4-^**

**1502.8**

**before reaction 7j**

**[M-3H]^3-^**

**2003.6**

**7j**

**[M-4H]^5-^**

**1516.6**

**at 30 min 8j**

**[M-5H]^6-^**

**1212.2**

**[M-3H]^3-^**

**2022.6**

**8j**

**7j**

**Calculated and found MS data**

| calculated mass | | calculated m/z | | |
| --- | --- | --- | --- | --- |
| sequence | exact mass | [M-3H]^3-^ | [M-4H]^4-^ | [M-5H]^5-^ |
| **7j** | 6012.8 | 2003.6 | 1502.4 | 1201.8 |
| **8j** | 6068.8 | 2022.3 | 1516.5 | 1213.0 |
| **before reaction** | | found m/z | | |
| sequence | LC RT^a^ | [M-3H]^3-^ | [M-4H]^4-^ | [M-5H]^5-^ |
| **7j** | 12.872 | 2003.6 | 1502.8 |  |
| **at 30 min** | | found m/z | | |
| sequence | LC RT | [M-3H]^3-^ | [M-4H]^4-^ | [M-5H]^5-^ |
| **8j** | 13.586 | 2022.6 | 1516.6 | 1212.2 |

**Supplementary Figure 36.** HPLC-MS analysis of acetonylation of **7j.** Elution method (solvent A: 5 mM TEAA, solvent B: acetonitrile): 0/0-34/20-35/90-38/90-40/0 (time [min]/Solvent B%) with flowrate 0.3 mL/min. ^a^RT = retention time.

**before reaction 7k**

**[M-4H]^4-^**

**1838.0**

**[M-3H]^3-^**

**2452.0**

**[M-5H]^5-^**

**1470.0**

**7k**

**at 30 min 8k**

**[M-4H]^4-^**

**1852.4**

**[M-5H]^5-^**

**1461.0**

**[M-6H]^6-^**

**1233.8**

**8k**

**7k**

**Calculated and found MS data**

| calculated mass | | calculated m/z | | | |
| --- | --- | --- | --- | --- | --- |
| sequence | exact mass | [M-3H]^3-^ | [M-4H]^4-^ | [M-5H]^5-^ | [M-6H]^6-^ |
| **7k** | 7354.3 | 2451.1 | 1838.1 | 1470.2 | 1225.0 |
| **8k** | 7426.3 | 2469.8 | 1852.1 | 1481.4 | 1234.4 |
| **before reaction** | | found m/z | | | |
| sequence | LC RT^a^ | [M-3H]^3-^ | [M-4H]^4-^ | [M-5H]^5-^ | [M-6H]^6-^ |
| **7k** | 10.718 | 2452.0 | 1838.0 | 1470.0 |  |
| **at 30 min** | | found m/z | | | |
| sequence | LC RT | [M-3H]^3-^ | [M-4H]^4-^ | [M-5H]^5-^ | [M-6H]^6-^ |
| **8k** | 10.991 |  | 1852.4 | 1481.0 | 1233.8 |

**Supplementary Figure 37.** HPLC-MS analysis of acetonylation of **7k.** Elution method (solvent A: 5 mM TEAA, solvent B: acetonitrile): 0/0-14/20-15/90-19/90-20/0 (time [min]/Solvent B%) with flowrate 0.3 mL/min. ^a^RT = retention time.

**before reaction**

**S1**

**S2**

**at 30 min**

**S2**

**S1**

**S1-act**

**before reaction**

**at 30 min**

**S1**  **S1-act**

**[M-4H]^4-^**

**1645.4**

**[M-5H]^5-^**

**1316.4**

**[M-3H]^3-^**

**2194.8**

**[M-3H]^3-^**

**2175.8**

**[M-4H]^4-^**

**1631.6**

**[M-5H]^5-^**

**1305.0**

**[M-6H]^6-^**

**1087.4**

**S2 S2**

**[M-4H]^4-^**

**1504.8**

**[M-4H]^4-^**

**1505.2**

**[M-3H]^3-^**

**2007.0**

**[M-6H]^6-^**

**1002.6**

**[M-5H]^5-^**

**1203.6**

**[M-3H]^3-^**

**2006.6**

**[M-5H]^5-^**

**1203.2**

**Calculated and found MS data**

| **calculated mass** | | calculated m/z | | | | |
| --- | --- | --- | --- | --- | --- | --- |
| sequence | exact mass | [M-2H]^2-^ | [M-3H]^3-^ | [M-4H]^4-^ | [M-5H]^5-^ | [M-6H]^6-^ |
| **S1** | 6529.1 | 3264.6 | 2176.0 | 1631.8 | 1305.2 | 1087.5 |
| **S2** | 6022.0 | 3011.0 | 2007.0 | 1505.0 | 1203.8 | 1003.0 |
| **S1-act** | 6585.2 | 3292.6 | 2194.7 | 1645.8 | 1316.4 | 1096.9 |
| **before reaction** | | found m/z | | | | |
| sequence | LC RT^a^ | [M-2H]^2-^ | [M-3H]^3-^ | [M-4H]^4-^ | [M-5H]^5-^ | [M-6H]^6-^ |
| **S1** | 21.920 |  | 2175.8 | 1631.6 | 1305.0 | 1087.4 |
| **S2** | 24.253 |  | 2006.6 | 1504.8 | 1203.2 |  |
| **at 30 min** | | found m/z | | | | |
| sequence | LC RT | [M-2H]^2-^ | [M-3H]^3-^ | [M-4H]^4-^ | [M-5H]^5-^ | [M-6H]^6-^ |
| **S1-act** | 22.344 |  | 2194.8 | 1645.4 | 1316.4 |  |
| **S2** | 24.324 |  | 2007.0 | 1505.2 | 1203.6 | 1002.6 |

**Supplementary Figure 38.** HPLC-MS analysis of acetonylation of **7l.** Elution method (solvent A: 5 mM TEAA, solvent B: acetonitrile): 0/0-33/15-34/90-38/90-40/0 (time [min]/Solvent B%) with flowrate 0.3 mL/min. ^a^RT = retention time.

**before reaction**

**S1**

**S2**

**S2**

**S1-act**

**at 30 min**

**at 30 min**

**before reaction**

**S1 S1-act**

**[M-5H]^5-^**

**1600.0**

**[M-5H]^5-^**

**1589.2**

**[M-4H]^4-^**

**1986.6**

**[M-6H]^6-^**

**1333.0**

**[M-4H]^4-^**

**2000.2**

**[M-6H]^6-^**

**1323.0**

**S2 S2**

**[M-5H]^5-^**

**1538.2**

**[M-4H]^4-^**

**1923.0**

**[M-6H]^6-^**

**1281.6**

**[M-5H]^5-^**

**1538.4**

**[M-4H]^4-^**

**1923.0**

**[M-6H]^6-^**

**1281.8**

**Calculated and found MS data**

| **calculated mass** | | calculated m/z | | | | |
| --- | --- | --- | --- | --- | --- | --- |
| sequence | exact mass | [M-2H]^2-^ | [M-3H]^3-^ | [M-4H]^4-^ | [M-5H]^5-^ | [M-6H]^6-^ |
| **S1** | 7941.3 | 3973.7 | 2648.8 | 1986.3 | 1588.9 | 1323.9 |
| **S2** | 7694.3 | 3847.2 | 2564.4 | 1923.1 | 1538.3 | 1281.7 |
| **S1-act** | 8003.4 | 4001.7 | 2667.5 | 2000.3 | 1600.1 | 1333.2 |
| **before reaction** | | found m/z | | | | |
| sequence | LC RT^a^ | [M-2H]^2-^ | [M-3H]^3-^ | [M-4H]^4-^ | [M-5H]^5-^ | [M-6H]^6-^ |
| **S1** | 22.879 |  |  | 1986.6 | 1589.2 | 1323.0 |
| **S2** | 21.819 |  |  | 1923.0 | 1538.2 | 1281.8 |
| **at 30 min** | | found m/z | | | | |
| sequence | LC RT | [M-2H]^2-^ | [M-3H]^3-^ | [M-4H]^4-^ | [M-5H]^5-^ | [M-6H]^6-^ |
| **S1-act** | 23.293 |  |  | 2000.2 | 1600.0 | 1333.0 |
| **S2** | 21.813 |  |  | 1923.0 | 1538.4 | 1281.6 |

**Supplementary Figure 39.** HPLC-MS analysis of acetonylation of **7m.** Elution method (solvent A: 5 mM TEAA, solvent B: acetonitrile): 0/0-33/15-34/90-38/90-40/0 (time [min]/Solvent B%) with flowrate 0.3 mL/min. ^a^RT = retention time.

**before reaction**

**acetonylation at 30 min**

**oxime ether at 1 h**

**S1-act, S2-act**

**S1, S2**

**S1-OX, S2-OX**

**S1, S2**

*Derivatized to the oxime ether for the separation of overlapping peaks

**oxime ether at 1 h**

**acetonylation at 30 min**

**before reaction**

**S1**  **S1-act**  **S1-OX**

**[M-6H]^6-^**

**1899.4**

**[M-5H]^5-^**

**2290.6**

**[M-5H]^5-^**

**2279.2**

**[M-6H]^6-^**

**1908.4**

**[M-6H]^6-^**

**1925.6**

**S2**  **S2-act**  **S2-OX**

**[M-6H]^6-^**

**1920.6**

**[M-6H]^6-^**

**1893.4**

**[M-5H]^5-^**

**2272.8**

**[M-7H]^7-^**

**1631.0**

**[M-5H]^5-^**

**2284.0**

**Calculated and found MS data**

| calculated mass | | calculated m/z | | |
| --- | --- | --- | --- | --- |
| sequence | exact mass | [M-5H]^5-^ | [M-6H]^6-^ | [M-7H]^7-^ |
| **S1** | 11396.9 | 2279.0 | 1899.0 | 1627.6 |
| **S2** | 11366.0 | 2273.0 | 1894.0 | 1623.3 |
| **S1-act** | 11453.0 | 2290.4 | 1908.3 | 1635.6 |
| **S1-OX** | 11558.0 | 2311.2 | 1925.8 | 1650.6 |
| **S2-act** | 11422.0 | 2284.0 | 1903.2 | 1631.1 |
| **S2-OX** | 11527.0 | 2305.2 | 1920.8 | 1646.3 |
| **before reaction** | | found m/z | | |
| sequence | LC RT^a^ | [M-5H]^5-^ | [M-6H]^6-^ | [M-7H]^7-^ |
| **S1** | 19.239 | 2279.2 | 1899.4 |  |
| **S2** | 19.239 | 2272.8 | 1893.4 |  |
| **acetonylation at 30 min** | | found m/z | | |
| sequence | LC RT | [M-5H]^5-^ | [M-6H]^6-^ | [M-7H]^7-^ |
| **S1-act** | 19.124 | 2290.6 | 1908.4 | 1635.4 |
| **S2-act** | 19.124 | 2284.0 |  | 1631.0 |
| **oxime ether 1 h** | | found m/z | | |
| sequence | LC RT | [M-5H]^5-^ | [M-6H]^6-^ | [M-7H]^7-^ |
| **S1-OX** | 21.029 |  | 1925.6 |  |
| **S2-OX** | 21.029 |  | 1920.6 |  |

**Supplementary Figure 40.** HPLC-MS analysis of acetonylation of **7n.** Elution method (solvent A: 20 mM TEAA, solvent B: acetonitrile): 0/0-33/20-35/90-38/90-40/0 (time [min]/Solvent B%) with flowrate 0.3 mL/min. ^a^RT = retention time.

**before reaction**

**8o**

**8o-OX**

**oxime ether at 1 h**

**acetonylation at 30 min**

**8o**

**7o**

*Derivatized to the oxime ether for the separation of overlapping peaks

**7o** **8o** **8o-OX**

**[M-5H]^5-^**

**2025.4**

**[M-6H]^6-^**

**1687.4**

**[M-6H]^6-^**

**1696.8**

**[M-5H]^5-^**

**2036.4**

**[M-5H]^5-^**

**2057.8**

**[M-6H]^6-^**

**1714.2**

**Calculated and found mass data**

| calculated mass | | calculated m/z | | |
| --- | --- | --- | --- | --- |
| sequence | exact mass | [M-5H]^5-^ | [M-6H]^6-^ | [M-7H]^7-^ |
| **7o** | 10128.7 | 2025.3 | 1687.6 | 1446.4 |
| **8o** | 10184.7 | 2036.5 | 1696.9 | 1454.4 |
| **8o-OX** | 10289.8 | 2057.6 | 1714.5 | 1469.4 |
| **before reaction** | | found m/z | | |
| sequence | LC RT^a^ | [M-5H]^5-^ | [M-6H]^6-^ | [M-7H]^7-^ |
| **7o** | 23.336 | 2025.4 | 1687.4 |  |
| **acetonylation at 30 min** | | found m/z | | |
| sequence | LC RT | [M-5H]^5-^ | [M-6H]^6-^ | [M-7H]^7-^ |
| **8o** | 23.837 | 2036.4 | 1696.8 | 1453.6 |
| **oxime ether at 1 h** | | found m/z | | |
| sequence | LC RT | [M-5H]^5-^ | [M-6H]^6-^ | [M-7H]^7-^ |
| **8o-OX** | 26.126 | 2057.8 | 1714.2 |  |

**Supplementary Figure 41.** HPLC-MS analysis of acetonylation of **7o.** Elution method (solvent A: 5 mM TEAA, solvent B: acetonitrile): 0/0-33/15-34/90-38/90-40/0 (time [min]/Solvent B%) with flowrate 0.3 mL/min. ^a^RT = retention time.

**before reaction**

**S2**

**S1**

**acetonylation at 30 min**

**S2**

**S1**

**acetonylation at 30 min**

**before reaction**

**S1**  **S1**

**[M-5H]^5-^**

**1523.0**

**[M-5H]^5-^**

**1523.0**

**[M-4H]^4-^**

**1904.0**

**[M-6H]^6-^**

**1268.8**

**[M-6H]^6-^**

**1269.0**

**[M-4H]^4-^**

**1903.6**

**S2**  **S2**

**[M-5H]^5-^**

**1538.0**

**[M-5H]^5-^**

**1538.0**

**[M-6H]^6-^**

**1282.0**

**[M-4H]^4-^**

**1923.0**

**[M-6H]^6-^**

**1281.6**

**[M-4H]^4-^**

**1923.0**

**Calculated and found MS data**

| calculated mass | | calculated m/z | | |
| --- | --- | --- | --- | --- |
| sequence | exact mass | [M-4H]^4-^ | [M-5H]^5-^ | [M-6H]^6-^ |
| **S1** | 7618.3 | 1904.1 | 1523.1 | 1269.0 |
| **S2** | 7694.3 | 1923.1 | 1538.3 | 1281.7 |
| **before reaction** | | found m/z | | |
| sequence | LC RT^a^ | [M-4H]^4-^ | [M-5H]^5-^ | [M-6H]^6-^ |
| **S1** | 22.960 | 1903.6 | 1523.0 | 1269.0 |
| **S2** | 21.760 | 1923.0 | 1538.0 | 1281.6 |
| **at 30 min** | | found m/z | | |
| sequence | LC RT | [M-4H]^4-^ | [M-5H]^5-^ | [M-6H]^6-^ |
| **S1** | 23.075 | 1904.0 | 1523.0 | 1268.8 |
| **S2** | 21.755 | 1923.0 | 1538.0 | 1282.0 |

**Supplementary Figure 42.** HPLC-MS analysis of acetonylation of **7p.** Elution method (solvent A: 5 mM TEAA, solvent B: acetonitrile): 0/0-33/15-34/90-38/90-40/0 (time [min]/Solvent B%) with flowrate 0.3 mL/min. ^a^RT = retention time.

**before reaction**

**S1**

**S2**

**S1-OX**

**oxime ether at 30 min**

**acetonylation at 30 min**

**S2**

**S1-act**

**S2**

**S1**

*Derivatized to the oxime ether for the separation of overlapping peaks

**oxime ether at 30 min**

**acetonylation at 30 min**

**before reaction**

**S1** **S1-act**  **S1-OX**

**[M-8H]^8-^
865.4**

**[M-4H]^4-^
1705.0**

**[M-4H]^4-^
1731.4**

**[M-7H]^7-^
989.0**

**[M-5H]^5-^
1385.0**

**[M-6H]^6-^
1153.8**

**[M-5H]^5-^
1363.6**

**[M-6H]^6-^
1136.2**

**[M-7H]^7-^
974.2**

**[M-8H]^8-^
852.0**

**[M-8H]^8-^
845.0**

**[M-4H]^4-^
1691.2**

**[M-5H]^5-^
1353.0**

**[M-6H]^6-^
1127.0**

**[M-7H]^7-^
966.0**

**S2** **S2** **S2**

**[M-4H]^4-^
1505.0**

**[M-4H]^4-^
1504.8**

**[M-7H]^7-^
859.8**

**[M-4H]^4-^
1504.6**

**[M-5H]^5-^
1203.6**

**[M-6H]^6-^
1002.8**

**[M-7H]^7-^
859.4**

**[M-8H]^8-^
752.0**

**[M-5H]^5-^
1203.4**

**[M-6H]^6-^
1002.8**

**[M-8H]^8-^
751.8**

**[M-5H]^5-^
1203.6**

**[M-6H]^6-^
1003.0**

**[M-7H]^7-^
859.6**

**[M-8H]^8-^
751.8**

**Calculated and found MS data**

| calculated mass | | calculated m/z | | | | |
| --- | --- | --- | --- | --- | --- | --- |
| sequence | exact mass | [M-4H]^4-^ | [M-5H]^5-^ | [M-6H]^6-^ | [M-7H]^7-^ | [M-8H]^8-^ |
| S1 | 6766.9 | 1691.2 | 1352.8 | 1127.1 | 966.0 | 845.1 |
| S2 | 6021.0 | 1505.0 | 1203.8 | 1003.0 | 859.6 | 752.0 |
| S1-act | 6823.0 | 1705.2 | 1364.0 | 1136.5 | 974.0 | 852.1 |
| S1-OX | 6928.0 | 1731.5 | 1385.0 | 1154.0 | 989.0 | 865.2 |
| **before reaction** | | found m/z | | | | |
| sequence | LC RT^a^ | [M-4H]^4-^ | [M-5H]^5-^ | [M-6H]^6-^ | [M-7H]^7-^ | [M-8H]^8-^ |
| S1 | 23.347 | 1691.2 | 1353.0 | 1127.0 | 965.8 | 845.0 |
| S2 | 28.347 | 1504.8 | 1203.4 | 1002.8 | 859.8 | 751.8 |
| **acetonylation at 30 min** | | found m/z | | | | |
| sequence | LC RT | [M-4H]^4-^ | [M-5H]^5-^ | [M-6H]^6-^ | [M-7H]^7-^ | [M-8H]^8-^ |
| S1-act | 23.597 | 1705.0 | 1363.6 | 1136.4 | 974.0 | 852.0 |
| S2 | 28.303 | 1504.6 | 1203.6 | 1003.0 | 859.6 | 751.8 |
| **oxime ether at 30 min** | | found m/z | | | | |
| sequence | LC RT | [M-4H]^4-^ | [M-5H]^5-^ | [M-6H]^6-^ | [M-7H]^7-^ | [M-8H]^8-^ |
| S1-OX | 27.122 | 1731.4 | 1385.2 | - | 988.8 | 865.2 |
| S2 | 28.429 | 1505.0 | 1203.8 | 1002.8 | 859.4 | 752.0 |

**Supplementary Figure 43.** HPLC-MS analysis of acetonylation of **7q.** Elution method (solvent A: 5 mM TEAA, solvent B: acetonitrile): 0/0-55/20-56/90-59/90-60/0 (time [min]/Solvent B%) with flowrate 0.3 mL/min. ^a^RT = retention time.

Supplementary Figure 44 – 50. Selected reactions in Supplementary Table 4

**before reaction**

**S1**

**S2**

**S1-act**

**S1**

**acetonylation at 30 min**

**S2**

**before reaction**

**acetonylation at 30 min**

**S1** **S1-act**

**[M-4H]^4-^**

**1646.0**

**[M-5H]^5-^**

**1305.0**

**[M-3H]^3-^**

**2194.6**

**[M-5H]^5-^**

**1316.2**

**[M-3H]^3-^**

**2176.4**

**[M-6H]^6-^**

**1087.8**

**[M-4H]^4-^**

**1631.6**

**S2**  **S2**

**[M-4H]^4-^**

**1504.8**

**[M-4H]^4-^**

**1504.8**

**[M-3H]^3-^**

**2006.6**

**[M-5H]^5-^**

**1203.8**

**[M-3H]^3-^**

**2006.8**

**[M-5H]^5-^**

**1203.4**

**Calculated and found MS data**

| calculated mass | | calculated m/z | | | | |
| --- | --- | --- | --- | --- | --- | --- |
| sequence | exact mass | [M-2H]^2-^ | [M-3H]^3-^ | [M-4H]^4-^ | [M-5H]^5-^ | [M-6H]^6-^ |
| **S1** | 6529.1 | 3264.6 | 2176.0 | 1631.8 | 1305.2 | 1087.5 |
| **S2** | 6022.0 | 3011.0 | 2007.0 | 1505.0 | 1203.8 | 1003.0 |
| **S1-act** | 6585.2 | 3292.6 | 2194.7 | 1645.8 | 1316.4 | 1096.9 |
| **before reaction** | | found m/z | | | | |
| sequence | LC RT^a^ | [M-2H]^2-^ | [M-3H]^3-^ | [M-4H]^4-^ | [M-5H]^5-^ | [M-6H]^6-^ |
| **S1** | 22.136 |  | 2176.4 | 1631.6 | 1305.0 | 1087.8 |
| **S2** | 24.543 |  | 2006.8 | 1504.8 | 1203.4 |  |
| **acetonylation at 30 min** | | found m/z | | | | |
| sequence | LC RT | [M-2H]^2-^ | [M-3H]^3-^ | [M-4H]^4-^ | [M-5H]^5-^ | [M-6H]^6-^ |
| **S1-act** | 23.092 |  | 2194.6 | 1646.0 | 1316.2 |  |
| **S2** | 24.352 |  | 2006.6 | 1504.8 | 1203.8 |  |

**Supplementary Figure 44.** HPLC-MS analysis of acetonylation of **18b.** Elution method (solvent A: 5 mM TEAA, solvent B: acetonitrile): 0/0-33/15-34/90-38/90-40/0 (time [min]/Solvent B%) with flowrate 0.3 mL/min. ^a^RT = retention time.

**before reaction**

**acetonylation at 30 min**

**S1-act**

**S2**

**S2**

**S1**

**before reaction**

**acetonylation at 30 min**

**S1** **S1-act**

**[M-4H]^4-^**

**1986.4**

**[M-5H]^5-^**

**1600.0**

**[M-4H]^4-^**

**2000.2**

**[M-5H]^5-^**

**1588.8**

**S2**  **S2**

**[M-4H]^4-^**

**1922.8**

**[M-3H]^3-^**

**2565.0**

**[M-5H]^5-^**

**1537.8**

**[M-4H]^4-^**

**1922.4**

**[M-5H]^5-^**

**1537.6**

**Calculated and found MS data**

| calculated mass | | calculated m/z | | | | |
| --- | --- | --- | --- | --- | --- | --- |
| sequence | exact mass | [M-2H]^2-^ | [M-3H]^3-^ | [M-4H]^4-^ | [M-5H]^5-^ | [M-6H]^6-^ |
| **S1** | 7947.3 | 3973.7 | 2648.8 | 1986.3 | 1588.9 | 1323.9 |
| **S2** | 7694.3 | 3847.2 | 2564.4 | 1923.1 | 1538.3 | 1281.7 |
| **S1-act** | 8003.4 | 4001.7 | 2667.5 | 2000.3 | 1600.1 | 1333.2 |
| **Before reaction** | | found m/z | | | | |
| sequence | LC RT^a^ | [M-2H]^2-^ | [M-3H]^3-^ | [M-4H]^4-^ | [M-5H]^5-^ | [M-6H]^6-^ |
| **S1** | 17.627 |  |  | 1986.4 | 1588.8 |  |
| **S2** | 17.147 |  | 2565.0 | 1922.4 | 1537.8 |  |
| **at 30 min** | | found m/z | | | | |
| sequence | LC RT | [M-2H]^2-^ | [M-3H]^3-^ | [M-4H]^4-^ | [M-5H]^5-^ | [M-6H]^6-^ |
| **S1-act** | 17.987 |  |  | 2000.2 | 1600.0 |  |
| **S2** | 17.161 |  |  | 1922.8 | 1537.6 |  |

**Supplementary Figure 45.** HPLC-MS analysis of acetonylation of **18c.** Elution method (solvent A: 5 mM TEAA, solvent B: acetonitrile): 0/0-30/20-35/90-38/90-40/0 (time [min]/Solvent B%) with flowrate 0.3 mL/min. ^a^RT = retention time

**before reaction**

**S2**

**S1**

**oxime ether at 1 h**

**S2**

**S1-OX**

**acetonylation at 30 min**

**S1-act, S2**

*Derivatized to the oxime ether for the separation of overlapping peaks

**before reaction**

**acetonylation at 30 min**

**oxime ether at 1 h**

**S1**  **S1-act S1-OX**

**[M-4H]^4-^**

**1640.2**

**[M-5H]^5-^**

**1300.4**

**[M-4H]^4-^**

**1666.0**

**[M-5H]^5-^**

**1332.8**

**[M-6H]^6-^**

**1110.2**

**[M-5H]^5-^**

**1311.6**

**[M-6H]^6-^**

**1083.6**

**[M-4H]^4-^**

**1625.4**

**S2 S2 S2**

**[M-4H]^4-^**

**1511.4**

**[M-4H]^4-^**

**1511.4**

**[M-4H]^4-^**

**1511.4**

**[M-6H]^6-^**

**1007.4**

**[M-6H]^6-^**

**1007.0**

**[M-5H]^5-^**

**1208.8**

**[M-5H]^5-^**

**1208.4**

**[M-6H]^6-^**

**1006.8**

**[M-5H]^5-^**

**1208.6**

**Calculated and found MS data**

| calculated mass | | calculated m/z | | | | |
| --- | --- | --- | --- | --- | --- | --- |
| sequence | exact mass | [M-2H]^2-^ | [M-3H]^3-^ | [M-4H]^4-^ | [M-5H]^5-^ | [M-6H]^6-^ |
| **S1** | 6506.1 | 3253.1 | 2168.4 | 1626.0 | 1300.6 | 1083.7 |
| **S2** | 6048.0 | 3024.0 | 2015.7 | 1511.5 | 1209.0 | 1007.3 |
| **S1-act** | 6562.2 | 3281.1 | 2187.0 | 1640.0 | 1311.8 | 1093.0 |
| **S1-OX** | 6667.2 | 3333.6 | 2222.1 | 1666.3 | 1332.8 | 1110.5 |
| **before reaction** | | found m/z | | | | |
| sequence | LC RT^a^ | [M-2H]^2-^ | [M-3H]^3-^ | [M-4H]^4-^ | [M-5H]^5-^ | [M-6H]^6-^ |
| **S1** | 21.665 |  | 2168.8 | 1625.4 | 1300.4 | 1083.6 |
| **S2** | 22.258 |  |  | 1511.4 | 1208.6 | 1007.4 |
| **at 30 min** | | found m/z | | | | |
| sequence | LC RT | [M-2H]^2-^ | [M-3H]^3-^ | [M-4H]^4-^ | [M-5H]^5-^ | [M-6H]^6-^ |
| **S1-act** | 22.027 |  |  | 1640.2 | 1311.6 |  |
| **S2** | 22.027 |  |  | 1511.4 | 1208.8 | 1007.0 |
| **oxime ether at 1 h** | | found m/z | | | | |
| sequence | LC RT | [M-2H]^2-^ | [M-3H]^3-^ | [M-4H]^4-^ | [M-5H]^5-^ | [M-6H]^6-^ |
| **S1-OX** | 24.634 |  |  | 1666.0 | 1332.8 | 1110.2 |
| **S2** | 22.034 |  |  | 1511.4 | 1208.8 | 1006.8 |

**Supplementary Figure 46.** HPLC-MS analysis of acetonylation of **18d.** Elution method (solvent A: 5 mM TEAA, solvent B: acetonitrile): 0/0-33/20-34/90-38/90-40/0 (time [min]/Solvent B%) with flowrate 0.3 mL/min. ^a^RT = retention time.

**before reaction**

**acetonylation at 30 min**

**S2**

**S1**

**oxime ether at 1 h**

**S2**

**S1-OX**

**S1-act**

**S2**

*Derivatized to the oxime ether for the separation of overlapping peaks

**oxime ether at 1 h**

**acetonylation at 30 min**

**before reaction**

MS of **S1**  **S1-act** **S1-OX**

**[M-4H]^4-^**

**1611.8**

**[M-4H]^4-^**

**1597.8**

**[M-3H]^3-^**

**2130.4**

**[M-3H]^3-^**

**2184.0**

**[M-4H]^4-^**

**1638.4**

**[M-3H]^3-^**

**2149.4**

**[M-5H]^5-^**

**1289.0**

**[M-6H]^6-^**

**1064.6**

**[M-5H]^5-^**

**1278.0**

**[M-5H]^5-^**

**1309.8**

**S2 S2 S2**

**[M-4H]^4-^**

**1538.6**

**[M-4H]^4-^**

**1538.8**

**[M-6H]^6-^**

**1025.4**

**[M-5H]^5-^**

**1230.4**

**[M-3H]^3-^**

**2051.4**

**[M-4H]^4-^**

**1538.8**

**[M-6H]^6-^**

**1025.8**

**[M-5H]^5-^**

**1230.8**

**Calculated and found MS data**

| calculated mass | | calculated m/z | | | | |
| --- | --- | --- | --- | --- | --- | --- |
| sequence | exact mass | [M-2H]^2-^ | [M-3H]^3-^ | [M-4H]^4-^ | [M-5H]^5-^ | [M-6H]^6-^ |
| **S1** | 6494.1 | 3197.1 | 2131.0 | 1598.0 | 1278.2 | 1065.0 |
| **S2** | 6158.1 | 3079.0 | 2052.3 | 1539.0 | 1231.0 | 1025.7 |
| **S1-act** | 6450.2 | 3225.1 | 2149.7 | 1612.0 | 1289.4 | 1074.4 |
| **S1-OX** | 6555.2 | 3277.6 | 2184.7 | 1638.3 | 1310.4 | 1091.9 |
| **before reaction** | | found m/z | | | | |
| sequence | LC RT^a^ | [M-2H]^2-^ | [M-3H]^3-^ | [M-4H]^4-^ | [M-5H]^5-^ | [M-6H]^6-^ |
| **S1** | 22.833 |  | 2130.4 | 1597.8 | 1278.0 | 1064.6 |
| **S2** | 23.046 |  |  | 1538.8 | 1230.8 | 1025.8 |
| **acetonylation at 30min** | | found m/z | | | | |
| sequence | LC RT | [M-2H]^2-^ | [M-3H]^3-^ | [M-4H]^4-^ | [M-5H]^5-^ | [M-6H]^6-^ |
| **S1-act** | 23.952 |  | 2149.4 | 1611.8 | 1289.0 |  |
| **S2** | 23.722 |  | 2051.4 | 1538.8 |  |  |
| **Oxime ether at 1 h** | | found m/z | | | | |
| sequence | LC RT | [M-2H]^2-^ | [M-3H]^3-^ | [M-4H]^4-^ | [M-5H]^5-^ | [M-6H]^6-^ |
| **S1-OX** | 26.243 |  | 2184.0 | 1638.4 | 1309.8 |  |
| **S2** | 23.490 |  |  | 1538.6 | 1230.4 | 1025.4 |

**Supplementary Figure 47.** HPLC-MS analysis of acetonylation of **18e.** Elution method (solvent A: 5 mM TEAA, solvent B: acetonitrile): 0/0-33/20-34/90-38/90-40/0 (time [min]/Solvent B%) with flowrate 0.3 mL/min. ^a^RT = retention time.

**acetonylation at 30 min**

**before reaction**

**S1**

**S2**

**oxime ether at 1 h**

**S1-OX**

**S2-OX**

**S1**

**S2**

**S1-act**

**S2-act**

*Derivatized to the oxime ether for the separation of overlapping peaks

**oxime ether at 1 h**

**acetonylation at 30 min**

**before reaction**

**S1**  **S1-act** **S1-OX**

**[M-5H]^5-^**

**1893.6**

**[M-5H]^5-^**

**1905.0**

**[M-5H]^5-^**

**1925.6**

**[M-6H]^6-^**

**1604.8**

**[M-6H]^6-^**

**1587.6**

**[M-6H]^6-^**

**1577.4**

**S2 S2-act S2-OX**

**[M-5H]^5-^**

**1948.8**

**[M-5H]^5-^**

**1917.0**

**[M-6H]^6-^**

**1623.8**

**[M-5H]^5-^**

**1928.0**

**[M-6H]^6-^**

**1596.8**

**Calculated and found mass data**

| calculated mass | | calculated m/z | | | | | |
| --- | --- | --- | --- | --- | --- | --- | --- |
| sequence | exact mass | [M-2H]^2-^ | [M-3H]^3-^ | [M-4H]^4-^ | [M-5H]^5-^ | [M-6H]^6-^ | [M-7H]^7-^ |
| **S1** | 9470.6 | 4735.8 | 3156.9 | 2367.4 | 1893.7 | 1577.9 | 1352.4 |
| **S2** | 9586.7 | 4793.8 | 3195.5 | 2396.4 | 1916.9 | 1597.3 | 1368.9 |
| **S1-act** | 9526.6 | 4763.8 | 3175.3 | 2381.4 | 1904.9 | 1587.3 | 1360.4 |
| **S1-OX** | 9631.7 | 4816.3 | 3210.6 | 2407.7 | 1925.9 | 1604.8 | 1375.4 |
| **S2-act** | 9642.7 | 4821.8 | 3214.2 | 2140.4 | 1928.1 | 1606.6 | 1376.9 |
| **S2-OX** | 9747.7 | 4874.4 | 3249.2 | 2436.7 | 1949.1 | 1624.1 | 1392.0 |
| **before reaction** | | found m/z | | | | | |
| sequence | LC RT^a^ | [M-2H]^2-^ | [M-3H]^3-^ | [M-4H]^4-^ | [M-5H]^5-^ | [M-6H]^6-^ | [M-7H]^7-^ |
| **S1** | 18.982 |  |  |  | 1893.6 | 1577.4 |  |
| **S2** | 19.422 |  |  |  | 1917.0 | 1596.8 |  |
| **acetonylation at 30 min** | | found m/z | | | | | |
| sequence | LC RT | [M-2H]^2-^ | [M-3H]^3-^ | [M-4H]^4-^ | [M-5H]^5-^ | [M-6H]^6-^ | [M-7H]^7-^ |
| **S1-act** | 19.851 |  |  |  | 1905.0 | 1587.6 |  |
| **S2-act** | 19.458 |  |  |  | 1928.0 |  |  |
| **oxime ether at 1 h** | | found m/z | | | | | |
| sequence | LC RT | [M-2H]^2-^ | [M-3H]^3-^ | [M-4H]^4-^ | [M-5H]^5-^ | [M-6H]^6-^ | [M-7H]^7-^ |
| **S1-OX** | 21.869 |  |  |  | 1925.6 | 1604.8 |  |
| **S2-OX** | 21.689 |  |  |  | 1948.8 | 1623.8 |  |

**Supplementary Figure 48.** HPLC-MS analysis of acetonylation of **18f.** Elution method (solvent A: 20 mM TEAA, solvent B: acetonitrile): 0/0-33/20-34/90-38/90-40/0 (time [min]/Solvent B%) with flowrate 0.3 mL/min. ^a^RT = retention time.

**before reaction**

**S1**

**S1-act**

**S2-act**

**S1, S2**

**S2-OX, S1-OX**

**oxime ether at 1 h**

**acetonylation at 30 min**

**S2**

*Derivatized to the oxime ether for the separation of overlapping peaks

**oxime ether at 1 h**

**acetonylation at 30 min**

**before reaction**

**S1** **S1-act**  **S1-OX**

**[M-5H]^5-^
1918.8**

**[M-5H]^5-^
1907.8**

**[M-7H]^7-^
1385.6**

**[M-6H]^6-^
1599.6**

**[M-5H]^5-^
1940.2**

**[M-6H]^6-^
1589.4**

**S2** **S2-act** **S2-OX**

**[M-5H]^5-^
1935.0**

**[M-7H]^7-^
1367.0**

**[M-5H]^5-^
1913.8**

**[M-6H]^6-^
1595.0**

**[M-5H]^5-^
1902.4**

**[M-6H]^6-^
1585.6**

**[M-7H]^7-^
1358.8**

**Calculated and found MS data**

| calculated mass | | calculated m/z | | |
| --- | --- | --- | --- | --- |
| sequence | exact mass | [M-5H]^5-^ | [M-6H]^6-^ | [M-7H]^7-^ |
| **S1** | 9541.6 | 1907.9 | 1589.8 | 1362.5 |
| **S2** | 9515.7 | 1902.7 | 1585.4 | 1358.8 |
| **S1-act** | 9597.6 | 1919.1 | 1599.1 | 1370.5 |
| **S1-OX** | 9702.7 | 1940.1 | 1616.6 | 1385.5 |
| **S2-act** | 9571.7 | 1913.9 | 1594.8 | 1366.8 |
| **S2-OX** | 9676.7 | 1934.9 | 1612.3 | 1381.8 |
| **before reaction** | | found m/z | | |
| sequence | LC RT^a^ | [M-5H]^5-^ | [M-6H]^6-^ | [M-7H]^7-^ |
| **S1** | 19.060 | 1907.8 | 1589.4 |  |
| **S2** | 18.614 | 1902.4 | 1585.6 | 1358.8 |
| **acetonylation at 30 min** | | found m/z | | |
| sequence | LC RT | [M-5H]^5-^ | [M-6H]^6-^ | [M-7H]^7-^ |
| **S1-act** | 19.210 | 1918.8 | 1599.6 |  |
| **S2-act** | 18.937 | 1913.8 | 1595.0 | 1367.0 |
| **oxime ether at 1 h** | | found m/z | | |
| sequence | LC RT | [M-5H]^5-^ | [M-6H]^6-^ | [M-7H]^7-^ |
| **S1-OX** | 21.239 | 1940.2 |  | 1385.6 |
| **S2-OX** | 21.112 | 1935.0 |  |  |

**Supplementary Figure 49.** HPLC-MS analysis of acetonylation of **18g.** Elution method (solvent A: 20 mM TEAA, solvent B: acetonitrile): 0/0-33/20-34/90-38/90-40/0 (time [min]/Solvent B%) with flowrate 0.3 mL/min. ^a^RT = retention time.

**before reaction**

**S2**

**S2-act**

**oxime ether at 1 h**

**S1-OX, S2-OX**

**S1, S2**

**S1-act**

**acetonylation at 30 min**

**S1**

*Derivatized to the oxime ether for the separation of overlapping peaks

**oxime ether at 1 h**

**acetonylation at 30 min**

**before reaction**

**S1** **S1-act**  **S1-OX**

**[M-5H]^5-^
1898.4**

**[M-5H]^5-^
1887.8**

**[M-6H]^6-^
1582.0**

**[M-7H]^7-^
1370.2**

**[M-5H]^5-^
1919.8**

**[M-7H]^7-^
1347.8**

**[M-6H]^6-^
1572.2**

**S2** **S2-act** **S2-OX**

**[M-5H]^5-^
1923.2**

**[M-6H]^6-^
1628.8**

**[M-5H]^5-^
1955.2**

**[M-7H]^7-^
1381.8**

**[M-6H]^6-^
1602.2**

**[M-6H]^6-^
1611.8**

**[M-5H]^5-^
1934.2**

**Calculated and found MS data**

| calculated mass | | calculated m/z | | |
| --- | --- | --- | --- | --- |
| sequence | exact mass | [M-5H]^5-^ | [M-6H]^6-^ | [M-7H]^7-^ |
| **S1** | 9439.6 | 1887.5 | 1572.8 | 1347.9 |
| **S2** | 9617.7 | 1923.1 | 1602.4 | 1373.4 |
| **S1-act** | 9495.6 | 1898.7 | 1582.1 | 1355.9 |
| **S1-OX** | 9600.7 | 1919.7 | 1599.6 | 1370.9 |
| **S2-act** | 9673.7 | 1934.3 | 1611.8 | 1381.4 |
| **S2-OX** | 9778.7 | 1955.3 | 1629.3 | 1396.4 |
| **before reaction** | | found m/z | | |
| sequence | LC RT^a^ | [M-5H]^5-^ | [M-6H]^6-^ | [M-7H]^7-^ |
| **S1** | 18.760 | 1887.8 | 1572.2 | 1347.8 |
| **S2** | 18.507 | 1923.2 | 1602.2 |  |
| **acetonylation at 30 min** | | found m/z | | |
| sequence | LC RT | [M-5H]^5-^ | [M-6H]^6-^ | [M-7H]^7-^ |
| **S1-act** | 19.106 | 1898.4 | 1582.0 |  |
| **S2-act** | 18.973 | 1934.2 | 1611.8 | 1381.8 |
| **oxime ether at 1 h** | | found m/z | | |
| sequence | LC RT | [M-5H]^5-^ | [M-6H]^6-^ | [M-7H]^7-^ |
| **S1-OX** | 21.174 | 1919.8 |  | 1370.2 |
| **S2-OX** | 21.174 | 1955.2 | 1628.8 |  |

**Supplementary Figure 50.** HPLC-MS analysis of acetonylation of **18h.** Elution method (solvent A: 20 mM TEAA, solvent B: acetonitrile): 0/0-33/20-34/90-38/90-40/0 (time [min]/Solvent B%) with flowrate 0.3 mL/min. ^a^RT = retention time.

Supplementary Figure 51 and 52. Modifications under high dilution conditions

**before reaction 7u**

**[M-3H]^3-^
1000.2**

**7u**

**[M-5H]^5-^**

**599.8**

**[M-2H]^2-^
1500.8**

**[M-4H]^4-^**

**750.0**

**at 3 h 8u**

**[M-3H]^3-^
1019.0**

**8u**

**7u**

**[M-5H]^5-^**

**560.8**

**[M-4H]^4-^**

**764.0**

**[M-2H]^2-^
1528.8**

**Calculated and found MS data**

| calculated mass | | calculated m/z | | | |
| --- | --- | --- | --- | --- | --- |
| sequence | exact mass | [M-2H]^2-^ | [M-3H]^3-^ | [M-4H]^4-^ | [M-5H]^5-^ |
| **7u** | 3003.5 | 1501.2 | 1000.5 | 750.1 | 599.9 |
| **8u** | 3059.5 | 1529.3 | 1019.2 | 764.1 | 611.1 |
| **before reaction** | | found m/z | | | |
| sequence | LC RT^a^ | [M-2H]^2-^ | [M-3H]^3-^ | [M-4H]^4-^ | [M-5H]^5-^ |
| **7u** | 10.974 | 1500.8 | 1000.2 | 750.0 | 599.8 |
| **at 3 h** | | found m/z | | | |
| sequence | LC RT | [M-2H]^2-^ | [M-3H]^3-^ | [M-4H]^4-^ | [M-5H]^5-^ |
| **8u** | 11.490 | 1528.7 | 1019.0 | 764.0 |  |

**Supplementary Figure 51.** HPLC-MS analysis of acetonylation of **7u** under dilute concentration**.** Elution method (solvent A: 5 mM TEAA, solvent B: acetonitrile): 0/0-14/20-15/90-18/90-20/0 (time [min]/Solvent B%) with flowrate 0.3 mL/min. ^a^RT = retention time.

**before reaction**

**S2**

**S1**

**S2**

**S1-act**

**S1**

**acetonylation at 30 min**

**acetonylation at 30 min**

**before reaction**

**S1** **S1-act**

**[M-5H]^5-^
1599.2**

**[M-6H]^6-^
1332.6**

**[M-4H]^4-^
2000.4**

**[M-4H]^4-^
1986.4**

**[M-5H]^5-^
1589.0**

**[M-6H]^6-^
1323.8**

**S2 S2**

**[M-5H]^5-^
1538.2**

**[M-5H]^5-^
1538.2**

**[M-4H]^4-^
1922.6**

**[M-4H]^4-^
1923.2**

**[M-6H]^6-^
1281.4**

**Calculated and found MS data**

| calculated mass | | calculated m/z | | | | |
| --- | --- | --- | --- | --- | --- | --- |
| sequence | exact mass | [M-2H]^2-^ | [M-3H]^3-^ | [M-4H]^4-^ | [M-5H]^5-^ | [M-6H]^6-^ |
| **S1** | 7947.3 | 3973.7 | 2648.8 | 1986.3 | 1588.9 | 1323.9 |
| **S2** | 7694.3 | 3847.2 | 2564.4 | 1923.1 | 1538.3 | 1281.7 |
| **S1-act** | 8003.4 | 4001.7 | 2667.5 | 2000.3 | 1600.1 | 1333.2 |
| **before reaction** | | found m/z | | | | |
| sequence | LC RT^a^ | [M-2H]^2-^ | [M-3H]^3-^ | [M-4H]^4-^ | [M-5H]^5-^ | [M-6H]^6-^ |
| **S1** | 23.487 |  |  | 1986.4 | 1589.0 | 1323.8 |
| **S2** | 22.740 |  |  | 1923.2 | 1538.2 | 1281.4 |
| **acetonylation at 3 h** | | found m/z | | | | |
| sequence | LC RT | [M-2H]^2-^ | [M-3H]^3-^ | [M-4H]^4-^ | [M-5H]^5-^ | [M-6H]^6-^ |
| **S1-act** | 23.593 |  |  | 2000.4 | 1599.2 | 1332.6 |
| **S2** | 22.413 |  |  | 1922.6 | 1538.2 |  |

**Supplementary Figure 52.** HPLC-MS analysis of acetonylation of **18c** under dilute concentration**.** Elution method (solvent A: 5 mM TEAA, solvent B: acetonitrile): 0/0-33/20-34/90-38/90-40/0 (time [min]/Solvent B%) with flowrate 0.3 mL/min. ^a^RT = retention time.

Supplementary Figure 53 and 54. Chemical ligation of ODNs

**before reaction**

**12b**

**12a, 12c**

**13c-OX**

**13b-OX**

**12a**

**oxime ether at 1 h**

**13c**

**12a**

**acetonylation at 30 min**

**13b**

*Derivatized to the oxime ether for the separation of overlapping peaks

**before reaction**

**12b** **12a** and **12c**

**acetonylation at 30 min**

**[M-4H]^-4^**

**1593.4**

**[M-5H]^-5^**

**1274.8**

**[M-6H]^-6^**

**1062.6**

**[M-11H]^-11^**

**977.0**

**[M-12H]^-12^**

**895.4**

**[M-14H]^-14^**

**767.6**

**[M-7H]^-7^**

**910.2**

**[M-13H]^-13^**

**826.4**

**[M-8H]^-8^**

**796.2**

**[M-3H]^-3^**

**1649.8**

**[M-4H]^-4^**

**1237.0**

**[M-5H]^-5^**

**989.4**

**[M-6H]^-6^**

**824.4**

**12a** **13b**  **13c**

**[M-13H]^-13^**

**826.6**

**[M-5H]^-5^**

**1285.8**

**[M-8H]^-8^**

**803.4**

**[M-6H]^-6^**

**1071.2**

**[M-7H]^-7^**

**918.0**

**[M-3H]^-3^**

**1668.6**

**[M-6H]^-6^**

**833.3**

**[M-4H]^-4^**

**1250.8**

**[M-5H]^-5^**

**1000.6**

**[M-12H]^-12^**

**895.4**

**[M-11H]^-11^**

**976.6**

**[M-14H]^-14^**

**767.2**

**[M-4H]^-4^**

**1607.8**

**oxime ether at 1 h**

**12a** **13b-OX** **13c-OX**

**[M-4H]^-4^**

**1634.0**

**[M-5H]^-5^**

**1306.8**

**[M-6H]^-6^**

**1088.8**

**[M-3H]^-3^**

**1704.0**

**[M-4H]^-4^**

**1277.2**

**[M-5H]^-5^**

**1021.6**

**[M-6H]^-6^**

**851.4**

**[M-11H]^-11^**

**976.8**

**[M-12H]^-12^**

**895.8**

**[M-14H]^-14^**

**767.2**

**[M-13H]^-13^**

**826.6**

**[M-7H]^-7^**

**933.2**

**[M-8H]^-8^**

**816.4**

**Calculated and found MS data**

| calculated mass | | calculated m/z | | | | | |
| --- | --- | --- | --- | --- | --- | --- | --- |
| sequence | exact mass | [M-11H]^11-^ | [M-12H]^12-^ | [M-13H]^13-^ | [M-14H]^14-^ |  |  |
| **12a** | 10754.8 | 977.0 | 895.5 | 826.5 | 767.4 |  |  |
| sequence | exact mass | [M-3H]^3-^ | [M-4H]^4-^ | [M-5H]^5-^ | [M-6H]^6-^ | [M-7H]^7-^ | [M-8H]^8-^ |
| **12b** | 4951.9 | 1650.0 | 1237.2 | 989.6 | 824.5 |  |  |
| **12c** | 6377.1 |  | 1593.8 | 1274.8 | 1062.2 | 910.3 | 796.4 |
| **13b** | 5007.9 | 1668.6 | 1251.2 | 1000.8 | 833.8 |  |  |
| **13c** | 6433.1 |  | 1607.8 | 1286.0 | 1071.5 | 918.3 | 803.4 |
| **13b-OX** | 5113.0 | 1703.6 | 1277.5 | 1021.8 | 851.3 |  |  |
| **13c-OX** | 6538.2 |  | 1634.0 | 1307.0 | 1089.0 | 933.3 | 816.5 |
| **before reaction** | | found m/z | | | | | |
| sequence | LC RT^a^ | [M-11H]^11-^ | [M-12H]^12-^ | [M-13H]^13-^ | [M-14H]^14-^ |  |  |
| **12a** | 27.384 | 977.0 | 895.4 | 826.4 | 767.6 |  |  |
| sequence | LC RT | [M-3H]^3-^ | [M-4H]^4-^ | [M-5H]^5-^ | [M-6H]^6-^ | [M-7H]^7-^ | [M-8H]^8-^ |
| **12b** | 24.051 | 1649.8 | 1237.0 | 989.4 | 824.4 |  |  |
| **12c** | 27.384 |  | 1593.4 | 1274.8 | 1062.6 | 910.2 | 796.2 |
| **acetonylation at 30 min** | | found m/z | | | | | |
| sequence | LC RT | [M-11H]^11-^ | [M-12H]^12-^ | [M-13H]^13-^ | [M-14H]^14-^ |  |  |
| **12a** | 27.228 | 976.6 | 895.4 | 826.6 | 767.2 |  |  |
| sequence | LC RT | [M-3H]^3-^ | [M-4H]^4-^ | [M-5H]^5-^ | [M-6H]^6-^ | [M-7H]^7-^ | [M-8H]^8-^ |
| **13b** | 25.275 | 1668.6 | 1250.8 | 1000.6 | 833.8 |  |  |
| **13c** | 27.688 |  | 1607.8 | 1285.8 | 1071.2 | 918.0 | 803.4 |
| **oxime ether at 1 h** | | found m/z | | | | | |
| sequence | LC RT | [M-11H]^11-^ | [M-12H]^12-^ | [M-13H]^13-^ | [M-14H]^14-^ |  |  |
| **12a** | 27.128 | 976.8 | 895.8 | 826.6 | 767.7 |  |  |
| sequence | LC RT | [M-3H]^3-^ | [M-4H]^4-^ | [M-5H]^5-^ | [M-6H]^6-^ | [M-7H]^7-^ | [M-8H]^8-^ |
| **13b-OX** | 29.294 | 1704.0 | 1277.2 | 1021.6 | 851.4 |  |  |
| **13c-OX** | 31.181 |  | 1634.0 | 1306.8 | 1088.8 | 933.2 | 816.4 |

**Supplementary Figure 53.** HPLC-MS analysis of double acetonylation of **12b** and **12c**. Elution method (solvent A: 5 mM TEAA, solvent B: acetonitrile): 0/0-55/20-56/90-59/90-60/0 (time [min]/solvent B). ^a^RT = retention time.

**before reaction**

**13c**

**13b**

**12a**

**12a**

**15**

**ligation at 1 h**

**before reaction**

**12a** **13b** **13c**

**[M-8H]^-8^**

**803.4**

**[M-6H]^-6^**

**1071.2**

**[M-7H]^-7^**

**918.0**

**[M-4H]^-4^**

**1607.8**

**[M-5H]^-5^**

**1285.8**

**[M-6H]^-6^**

**833.8**

**[M-5H]^-5^**

**1000.6**

**[M-3H]^-3^**

**1668.6**

**[M-4H]^-4^**

**1250.8**

**[M-13H]^-13^**

**826.6**

**[M-11H]^-11^**

**976.6**

**[M-12H]^-12^**

**895.4**

**[M-14H]^-14^**

**767.2**

**ligation at 1 h**

**12a** **15**

**[M-13H]^-14^**

**823.6**

**[M-14H]^-14^**

**767.6**

**[M-12H]^-12^**

**895.8**

**[M-11H]^-12^**

**961.0**

**[M-12H]^-13^**

**887.2**

**[M-14H]^-15^**

**768.8**

**[M-11H]^-11^**

**976.8**

**[M-13H]^-13^**

**826.6**

**Calculated and found MS data**

| calculated mass | | calculated m/z | | | | | |
| --- | --- | --- | --- | --- | --- | --- | --- |
| sequence | exact mass | [M-11H]^11-^ | [M-12H]^12-^ | [M-13H]^13-^ | [M-14H]^14-^ | [M-15H]^15-^ |  |
| **12a** | 10754.8 | 977.0 | 895.5 | 826.5 | 767.4 |  |  |
| sequence | exact mass | [M-3H]^3-^ | [M-4H]^4-^ | [M-5H]^5-^ | [M-6H]^6-^ | [M-7H]^7-^ | [M-8H]^8-^ |
| **13b** | 5007.9 | 1668.6 | 1251.2 | 1000.8 | 833.8 |  |  |
| **13c** | 6433.1 |  | 1607.8 | 1286.0 | 1071.5 | 918.3 | 803.4 |
| sequence | exact mass | [M-11H]^11-^ | [M-12H]^12-^ | [M-13H]^13-^ | [M-14H]^14-^ | [M-15H]^15-^ |  |
| **15** | 11539.1 |  | 961.1 | 887.0 | 823.6 | 768.6 |  |
| **before reaction** | | found m/z | | | | | |
| sequence | LC RT^a^ | [M-11H]^11-^ | [M-12H]^12-^ | [M-13H]^13-^ | [M-14H]^14-^ | [M-15H]^15-^ |  |
| **12a** | 27.228 | 976.6 | 895.4 | 826.6 | 767.2 |  |  |
| sequence | LC RT | [M-3H]^3-^ | [M-4H]^4-^ | [M-5H]^5-^ | [M-6H]^6-^ | [M-7H]^7-^ | [M-8H]^8-^ |
| **13b** | 25.275 | 1668.6 | 1250.8 | 1000.6 | 833.8 |  |  |
| **13c** | 27.688 |  | 1607.8 | 1285.8 | 1071.2 | 918.0 | 803.4 |
| **ligation at 1 h** | | found m/z | | | | | |
| sequence | LC RT | [M-11H]^11-^ | [M-12H]^12-^ | [M-13H]^13-^ | [M-14H]^14-^ | [M-15H]^15-^ |  |
| **12a** | 27.968 | 976.8 | 895.8 | 826.6 | 767.6 |  |  |
| **15** | 32.188 |  | 961.0 | 887.2 | 823.6 | 768.8 |  |

**Supplementary Figure 54.** HPLC-MS analysis of chemical ligation of **13b** and **13c**. Elution method (solvent A: 5 mM TEAA, solvent B: acetonitrile): 0/0-55/20-56/90-59/90-60/0 (time [min]/solvent B). ^a^RT = retention time.

Supplementary Figure 55 – 59. Synthesis of photocaged ONs and decaging

**before reaction**

**at 30 min**

**S1**

**S2**

**S2**

**S1-pHP**

**S1**

**at 30 min**

**before reaction**

**S1**  **S1-pHP**

**[M-8H]^8-^**

**861.8**

**[M-4H]^4-^**

**1725.2**

**[M-6H]^6-^**

**1149.4**

**[M-5H]^5-^**

**1379.2**

**[M-7H]^7-^**

**984.8**

**[M-4H]^4-^**

**1691.2**

**[M-5H]^5-^**

**1352.8**

**[M-6H]^6-^**

**1126.8**

**[M-7H]^7-^**

**966.2**

**[M-8H]^8-^**

**845.0**

**S2 S2**

**[M-4H]^4-^**

**1505.0**

**[M-5H]^5-^**

**1203.2**

**[M-6H]^6-^**

**1002.8**

**[M-7H]^7-^**

**859.4**

**[M-8H]^8-^**

**752.0**

**[M-7H]^7-^**

**859.4**

**[M-6H]^6-^**

**1002.6**

**[M-5H]^5-^**

**1203.6**

**[M-4H]^4-^**

**1504.8**

**[M-8H]^8-^**

**752.0**

**Calculated and found MS data**

| calculated mass | | calculated m/z | | | | |
| --- | --- | --- | --- | --- | --- | --- |
| sequence | exact mass | [M-4H]^4-^ | [M-5H]^5-^ | [M-6H]^6-^ | [M-7H]^7-^ | [M-8H]^8-^ |
| **S1** | 6766.9 | 1691.2 | 1352.8 | 1127.1 | 966.0 | 845.1 |
| **S1-pHP** | 6901.0 | 1724.7 | 1380.0 | 1149.5 | 985.1 | 861.9 |
| **S2** | 6021.0 | 1505.0 | 1203.8 | 1003.0 | 859.6 | 752.0 |
| **before reaction** | | found m/z | | | | |
| sequence | LC RT^a^ | [M-4H]^4-^ | [M-5H]^5-^ | [M-6H]^6-^ | [M-7H]^7-^ | [M-8H]^8-^ |
| **S1** | 23.262 | 1691.2 | 1352.8 | 1126.8 | 966.2 | 845.0 |
| **S2** | 28.162 | 1504.8 | 1203.6 | 1002.6 | 859.4 | 752.0 |
| **at 30 min** | | found m/z | | | | |
| sequence | LC RT | [M-4H]^4-^ | [M-5H]^5-^ | [M-6H]^6-^ | [M-7H]^7-^ | [M-8H]^8-^ |
| **S1** | 23.043 | 1691.0 | 1352.4 | 1127.2 | 966.0 | 845.0 |
| **S1-pHP** | 25.063 | 1725.2 | 1379.2 | 1149.4 | 984.8 | 861.8 |
| **S2** | 28.676 | 1505.0 | 1203.2 | 1002.8 | 859.4 | 752.0 |

**Supplementary Figure 55.** HPLC-MS analysis of photocaging of **7q**. Elution method (solvent A: 5 mM TEAA, solvent B: acetonitrile): 0/0-55/20-56/90-59/90-60/0 (time [min]/solvent B). ^a^RT = retention time.

**before reaction 7v**

**[M-3H]^3-^**

**994.0**

**[M-2H]^2-^**

**1492.0**

**7v**

**at 1 h**  **17v**

**[M-3H]^3-^**

**1038.8**

**17v**

**7v**

**Calculated and found MS data**

| calculated mass | | calculated m/z | | | | |
| --- | --- | --- | --- | --- | --- | --- |
| sequence | exact mass | [M-2H]^2-^ | [M-3H]^3-^ | [M-4H]^4-^ | [M-5H]^5-^ | [M-6H]^6-^ |
| **7v** | 2985.5 | 1492.3 | 994.5 | 745.6 | 596.3 | 496.8 |
| **17v** | 3119.6 | 1559.3 | 1039.1 | 779.1 | 623.1 | 519.1 |
| **before reaction** | | found m/z | | | | |
| sequence | LC RT^a^ | [M-2H]^2-^ | [M-3H]^3-^ | [M-4H]^4-^ | [M-5H]^5-^ | [M-6H]^6-^ |
| **7v** | 10.687 | 1492.0 | 994.0 |  |  |  |
| **at 1 h** | | found m/z | | | | |
| sequence | LC RT | [M-2H]^2-^ | [M-3H]^3-^ | [M-4H]^4-^ | [M-5H]^5-^ | [M-6H]^6-^ |
| **17v** | 11.601 |  | 1038.8 |  |  |  |

**Supplementary Figure 56.** HPLC-MS analysis of photocaging of **7v**. Elution method (solvent A: 5 mM TEAA, solvent B: acetonitrile): 0/0-14/20-15/90-19/90-20/0 (time [min]/solvent B). ^a^RT = retention time

**before reaction 7u**

**[M-3H]^3-^**

**1000.2**

**[M-2H]^2-^**

**1500.8**

**7u**

**at 1 h**   **17u**

**[M-3H]^3-^**

**1044.6**

**17u**

**[M-2H]^2-^1567.6**

**[M-4H]^4-^**

**783.0**

**7u**

**Calculated and found MS data**

| calculated mass | | calculated m/z | | | | |
| --- | --- | --- | --- | --- | --- | --- |
| sequence | exact mass | [M-2H]^2-^ | [M-3H]^3-^ | [M-4H]^4-^ | [M-5H]^5-^ | [M-6H]^6-^ |
| **7u** | 3003.5 | 1501.2 | 1000.5 | 750.1 | 599.9 | 499.7 |
| **17u** | 3137.5 | 1568.3 | 1045.2 | 783.6 | 626.7 | 522.1 |
| **before reaction** | | found m/z | | | | |
| sequence | LC RT^a^ | [M-2H]^2-^ | [M-3H]^3-^ | [M-4H]^4-^ | [M-5H]^5-^ | [M-6H]^6-^ |
| **7u** | 11.198 | 1500.8 | 1000.2 |  |  |  |
| **at 1 h** | | found m/z | | | | |
| sequence | LC RT | [M-2H]^2-^ | [M-3H]^3-^ | [M-4H]^4-^ | [M-5H]^5-^ | [M-6H]^6-^ |
| **17u** | 12.173 | 1567.6 | 1045.2 | 783.0 |  |  |

**Supplementary Figure 57.** HPLC-MS analysis of photocaging of **7u**. Elution method (solvent A: 5 mM TEAA, solvent B: acetonitrile): 0/0-14/20-15/90-19/90-20/0 (time [min]/solvent B). ^a^RT = retention time.

**before reaction 7b**

**[M-4H]^4-^**

**1759.6**

**[M-5H]^5-^**

**1407.2**

**7b**

**at 1 h**  **17b**

**[M-4H]^4-^**

**1792.8**

**[M-5H]^5-^**

**1434.2**

**7b**

**17b**

**Calculated and found MS data**

| calculated mass | | calculated m/z | | | | | |
| --- | --- | --- | --- | --- | --- | --- | --- |
| sequence | exact mass | [M-2H]^2-^ | [M-3H]^3-^ | [M-4H]^4-^ | [M-5H]^5-^ | [M-6H]^6-^ | [M-7H]^7-^ |
| **7b** | 7040.2 | 3520.1 | 2346.4 | 1759.5 | 1407.4 | 1172.7 | 1005.0 |
| **17b** | 7174.2 | 3587.1 | 2391.1 | 1793.1 | 1434.2 | 1195.0 | 1024.2 |
| **before reaction** | | found m/z | | | | | |
| sequence | LC RT^a^ | [M-2H]^2-^ | [M-3H]^3-^ | [M-4H]^4-^ | [M-5H]^5-^ | [M-6H]^6-^ | [M-7H]^7-^ |
| **7b** | 23.419 |  |  | 1759.6 | 1407.2 | 1172.8 |  |
| **at 1 h** | | found m/z | | | | | |
| sequence | LC RT | [M-2H]^2-^ | [M-3H]^3-^ | [M-4H]^4-^ | [M-5H]^5-^ | [M-6H]^6-^ | [M-7H]^7-^ |
| **17b** | 23.573 |  |  | 1792.8 | 1434.2 |  |  |

**Supplementary Figure 58.** HPLC-MS analysis of photocaging of **7b**. Elution method (solvent A: 5 mM TEAA, solvent B: acetonitrile): 0/0-33/15-34/90-38/90-40/0 (time [min]/solvent B). ^a^RT = retention time.

**before reaction**

**7b**

**7b**

**17b**

**at 10 min**

**Supplementary Figure 59.** HPLC-MS analysis of decaging of **17b**. Elution method (solvent A: 5 mM TEAA, solvent B: acetonitrile): 0/0-33/15-34/90-38/90-40/0 (time [min]/solvent B).

Supplementary Figure 60. Preparation of Rhodamine B and ketone functionalized oligonucleotides

**before reaction**

**S1-RB-act**

**S2**

**S2**

**S1-RB**

**S1-RB-OX**

**oxime ether at 1 h**

**acetonylation at 30 min**

**S1-RB**

**S2**

*Derivatized to the oxime ether for the separation of overlapping peaks

**acetonylation at 30 min**

**oxime ether at 1 h**

**before reaction**

MS of **S1-RB** **S1-RB-act S1-RB-OX**

**[M-6H]^6-^**

**2138.4**

**[M-5H]^5-^**

**2545.0**

**[M-6H]^6-^**

**2120.6**

**[M-5H]^5-^**

**2565.8**

**[M-7H]^7-^**

**1809.0**

**[M-6H]^6-^**

**2111.4**

**[M-5H]^5-^**

**2533.4**

**S2** **S2** **S2**

**[M-6H]^6-^**

**1943.6**

**[M-5H]^5-^**

**2332.0**

**[M-6H]^6-^**

**1943.6**

**[M-5H]^5-^**

**2332.0**

**[M-6H]^6-^**

**1943.4**

**[M-5H]^5-^**

**2332.2**

**Calculated and found MS data**

| calculated mass | | calculated m/z | | | | | |
| --- | --- | --- | --- | --- | --- | --- | --- |
| sequence | exact mass | [M-3H]^3-^ | [M-4H]^4-^ | [M-5H]^5-^ | [M-6H]^6-^ | [M-7H]^7-^ | [M-8H]^8-^ |
| **S1-RB** | 12669.4 | 4223.5 | 3167.4 | 2533.7 | 2111.2 | 1809.5 | 1583.2 |
| **S2** | 11663.0 | 3888.0 | 2915.7 | 2332.4 | 1943.5 | 1665.7 | 1457.4 |
| **S1-RB-act** | 12725.5 | 4242.2 | 3181.4 | 2544.9 | 2120.6 | 1817.5 | 1590.2 |
| **S1-RB-OX** | 12830.5 | 4277.2 | 3207.6 | 2565.9 | 2138.1 | 1832.5 | 1603.3 |
| **before reaction** | | found m/z | | | | | |
| sequence | LC RT^a^ | [M-3H]^3-^ | [M-4H]^4-^ | [M-5H]^5-^ | [M-6H]^6-^ | [M-7H]^7-^ | [M-8H]^8-^ |
| **S1-RB** | 22.828 |  |  | 2533.4 | 2111.4 | 1809.0 |  |
| **S2** | 18.401 |  |  | 2332.0 | 1943.4 |  |  |
| **acetonylation at 30 min** | | found m/z | | | | | |
| sequence | LC RT | [M-3H]^3-^ | [M-4H]^4-^ | [M-5H]^5-^ | [M-6H]^6-^ | [M-7H]^7-^ | [M-8H]^8-^ |
| **S1-RB-act** | 22.683 |  |  | 2545.0 | 2120.6 |  |  |
| **S2** | 18.463 |  |  | 2332.2 | 1943.6 |  |  |
| **oxime ether 1 h** | | found m/z | | | | | |
| sequence | LC RT | [M-3H]^3-^ | [M-4H]^4-^ | [M-5H]^5-^ | [M-6H]^6-^ | [M-7H]^7-^ | [M-8H]^8-^ |
| **S1-RB-OX** | 23.838 |  |  | 2565.8 | 2138.4 |  |  |
| **S2** | 18.405 |  |  | 2332.0 | 1943.6 |  |  |

**Supplementary Figure 60.** HPLC-MS analysis of preparation of Rhodamine B and ketone functionalized oligonucleotides. Elution method (solvent A: 5 mM TEAA, solvent B: acetonitrile): 0/0-33/20-34/90-38/90-40/0 (time [min]/solvent B). ^a^RT = retention time.

**Supplementary Figure 61.** Fluorescence emission spectrum of **22a**

11. NMR Spectra

^1^H NMR (400 MHz, DMSO-d_6_) spectrum of **6**

^13^C NMR (100 MHz, DMSO-d_6_) spectrum of **6**

^3^J_C4, H8_

^3^J_C5, H8_

^2^J_C, H9_

^3^J_C6, H9_

^1^J_C6, H6_

^1^J_C8, H8_

^3^J_C8, H1’_

Fragment of ^1^H-^13^C HMBC (DMSO-d_6_) spectrum of **6**

^1^H NMR (400 MHz, DMSO-d_6_) spectrum of **6b**

^13^C NMR (100 MHz, DMSO-d_6_) spectrum of **6b**

^3^J_C5, H9‘_

^3^J_C8, H9‘_

^2^J_C, H9_

^3^J_C6, H9_

^1^J_C8, H8_

^3^J_C4, H8_

^3^J_C5, H8_

Fragment of ^1^H-^13^C HMBC (DMSO-d_6_) spectrum of **6b**

^1^H NMR (400 MHz, CDCl_3_) spectrum of **25a**

^1^H NMR (400 MHz, CDCl_3_) spectrum of **5**

^1^H NMR (400 MHz, CDCl_3_) spectrum of **26a**

^1^H NMR (400 MHz, CDCl_3_) spectrum of **27a**

^1^H NMR (400 MHz, CDCl_3_) spectrum of **28a**

^1^H NMR (400 MHz, CDCl_3_) spectrum of **28b**

^1^H NMR (400 MHz, CDCl_3_) spectrum of **16**

^1^H NMR (400 MHz, CDCl_3_) spectrum of **29a**

^1^H NMR (400 MHz, CDCl_3_) spectrum of **29b**

^1^H NMR (400 MHz, MeOH-d_4_) spectrum of **29c**

^1^H NMR (400 MHz, MeOH-d_4_) spectrum of **29d**

^1^H NMR (400 MHz, CDCl_3_) spectrum of **30a**

^1^H NMR (400 MHz, CDCl_3_) spectrum of **30b**

^13^C NMR (100 MHz, CDCl_3_) spectrum of **30b**

^1^H NMR (400 MHz, D_2_O) spectrum of **14**

^13^C NMR (100 MHz, D_2_O) spectrum of **14**

12. DFT Calculations

**12.1 Computational details**

All DFT calculations were performed in the Gaussian 09 software (Rev D.01)^12^ using the M06 functional^13^. Geometry optimization was performed with the 6-31g(d) basis set^14,15^ for H, C, N, O, Cl atoms, and the LANL2DZ basis set^16^ for Rh, which were obtained from the EMSL Basis Set Exchange^17,18^. Frequency calculations were performed for every optimized geometry with the same level of theory to obtain vibrational frequencies and thermochemical data at 298.15 K. The SMD solvation model^19^ with the solvent of water (ε = 78.355) was used for all calculations. The transition states were identified by having one imaginary frequency, and intrinsic reaction coordinate^20,21^ (IRC) calculations were performed to connect transition states with corresponding intermediates. Each intermediate was verified as minima by having no imaginary frequency, and the geometries of intermediates with possibility of multiple conformations were optimized with several different starting geometries to find the lowest energy conformation. Nucleophilicity index was calculated by the previously reported method^22,23^.

**12.2 DFT studies of proposed reaction mechanisms**

DFT calculations on the metal carbene formation between diazoacetone and the rhodium catalyst were performed (Supplementary Fig. 62). The calculations show that the activation free energy of Rh(I)-associated carbene formation is 11.6 kcal/mol (**TS-1a**). Subsequently, the liberation of molecular nitrogen generates Rh(I)-carbene via exergonic pathway (-8.8 kcal/mol, **Int-1c**). Next, we examined the reaction between Rh(I)-carbene **Int-1c** and deoxyguanosine **Int-2a**. The nucleophilic attack of guanine O^6^ of **Int-2a** to Rh(I)-carbene **Int-1c** requires an activation energy of 6.4 kcal/mol (**TS-2a**) and generates zwitterionic intermediate **Int-2b** exergonically by -5.0 kcal/mol. Subsequent intramolecular proton transfer with the low activation barrier (0.5 kcal/mol, **TS-2b**) affords enol intermediate **Int-2c** with -0.2 kcal/mol change in free energy, and the catalyst is regenerated endergonically by 0.1 kcal/mol. Released enol intermediate **Int-2d** is activated by hydronium ion and tautomerizes to **Int-2e** through **TS-2c** with the activation barrier of 3.1 kcal/mol and the free energy change of -15.5 kcal/mol. Subsequent deprotonation of **Int-2e** generates final product **Int-2f** through -2.6 kcal/mol change in free energy.

|  |
| --- |
| **Supplementary Figure 62.** Energy profile of the reaction pathway by DFT calculations |

**12.3 Tables of energies in hartree**

| Structure | E | G_298_ |
| --- | --- | --- |
|  | -301.211407 | -301.170858 |
|  | -881.537805‬ | -881.390299 |
|  | -1,182.770635‬ | -1182.559159 |
|  | -1,182.747288‬ | -1182.540622 |
|  | -1,073.30676‬ | -1073.104755 |
|  | -109.455555 | -109.468376 |
|  | -963.022683 | -962.812067 |
|  | -2,036.345792‬ | -2035.906563 |
|  | -2,036.366903‬ | -2035.924810 |
|  | -2,036.363793‬ | -2035.923943 |
|  | -2,036.36992‬0 | -2035.925117 |
|  | -1154.804995 | -1154.534620 |
|  | -1,231.621169‬ | -1231.321339 |
|  | -1155.250151 | -1154.966902 |
|  | -1154.832989 | -1154.563430 |
|  | -76.807337 | -76.791619 |
|  | -76.387586 | -76.383985 |
|  | -506.307977 | -506.201924 |
|  | -433.983227 | -433.890855 |
|  | -581.530651 | -581.420949 |
|  | -493.150620 | -493.042532 |

13. Abbreviations

acac: acetylacetonate

act-G: O^6^-acetonylated deoxyguanosine

COD: 1,5-cyclooctadiene

DPC: DNA-protein cross-linking

dsDNA: double strand DNA

LC RT: LC retention time

MES: 2-(N-morpholino)ethanesulfonic acid

MOPS: 3-morpholinopropane-1-sulfonic acid

ON: oligonucleotide

ODN: oligodeoxyribonucleotide

ORN: oligoribonucleotide

PAGE: polyacrylamide gel electrophoresis

ssDNA: single strand DNA

ssODN: single strand oligodeoxyribonucleotide

T7 RNAP: T7 RNA polymerase

14. Supplementary References

1 Geigle, S. N., Wyss, L. A., Sturla, S. J. & Gillingham, D. G. Copper carbenes alkylate guanine chemoselectively through a substrate directed reaction. *Chem. Sci.* **8**, 499-506 (2017).

2 Pauly, G. T. & Moschel, R. C. Mutagenesis by O6-Methyl-, O6-Ethyl-, and O6-Benzylguanine and O4-Methylthymine in Human Cells:  Effects of O6-Alkylguanine-DNA Alkyltransferase and Mismatch Repair. *Chem. Res. Toxicol.* **14**, 894-900 (2001).

3 Wang, P. & Wang, Y. Cytotoxic and mutagenic properties of O6-alkyl-2′-deoxyguanosine lesions in Escherichia coli cells. *J. Biol. Chem.* **293**, 15033-15042 (2018).

4 Koskinen, A. M. P. & Muñoz, L. Diazo transfer reactions under mildly basic conditions. *J. Chem. Soc., Chem. Commun.*, 652-653 (1990).

5 Lancou, A. *et al.* α-Triethylsilyl-α-diazoacetone in double cross-aldolisation: convenient acetone equivalent toward 5-hydroxy-1,3-diketones. *Tetrahedron* **68**, 9652-9657 (2012).

6 Wada, H., Williams, H. E. L. & Moody, C. J. Total Synthesis of the Posttranslationally Modified Polyazole Peptide Antibiotic Plantazolicin A. *Angew. Chem. Int. Ed.* **54**, 15147-15151 (2015).

7 Koduri, N. D. *et al.* Ruthenium Catalyzed Synthesis of Enaminones. *Org. Lett.* **14**, 440-443 (2012).

8 Jiang, Q., Sheng, W. & Guo, C. Synthesis of phenacyl bromides via K2S2O8-mediated tandem hydroxybromination and oxidation of styrenes in water. *Green Chem.* **15**, 2175-2179 (2013).

9 Senadheera, S. N., Evans, A. S., Toscano, J. P. & Givens, R. S. 2-Diazo-1-(4-hydroxyphenyl)ethanone: a versatile photochemical and synthetic reagent. *Photochem. Photobiol. Sci.* **13**, 324-341 (2014).

10 Bindman, N. A. & van der Donk, W. A. A General Method for Fluorescent Labeling of the N-Termini of Lanthipeptides and Its Application to Visualize their Cellular Localization. *J. Am. Chem. Soc.* **135**, 10362-10371 (2013).

11 Chiba, K. *et al.* Specific fluorescence labeling of target proteins by using a ligand–4-azidophthalimide conjugate. *Chem. Commun.* **53**, 8751-8754 (2017).

12 Gaussian 09, Revision D.01 (2013).

13 Zhao, Y. & Truhlar, D. G. The M06 suite of density functionals for main group thermochemistry, thermochemical kinetics, noncovalent interactions, excited states, and transition elements: two new functionals and systematic testing of four M06-class functionals and 12 other functionals. *Theor. Chem. Acc.* **120**, 215-241 (2008).

14 Hariharan, P. C. & Pople, J. A. The influence of polarization functions on molecular orbital hydrogenation energies. *Theoret. Chim. Acta* **28**, 213-222 (1973).

15 Hehre, W. J., Ditchfield, R. & Pople, J. A. Self—Consistent Molecular Orbital Methods. XII. Further Extensions of Gaussian—Type Basis Sets for Use in Molecular Orbital Studies of Organic Molecules. *J. Chem. Phys.* **56**, 2257-2261 (1972).

16 Hay, P. J. & Wadt, W. R. Ab initio effective core potentials for molecular calculations. Potentials for K to Au including the outermost core orbitals. *J. Chem. Phys.* **82**, 299-310 (1985).

17 Feller, D. The role of databases in support of computational chemistry calculations. *J. Comput. Chem.* **17**, 1571-1586 (1996).

18 Schuchardt, K. L. *et al.* Basis Set Exchange:  A Community Database for Computational Sciences. *J. Chem. Inform. Model.* **47**, 1045-1052 (2007).

19 Marenich, A. V., Cramer, C. J. & Truhlar, D. G. Universal Solvation Model Based on Solute Electron Density and on a Continuum Model of the Solvent Defined by the Bulk Dielectric Constant and Atomic Surface Tensions. *J. Phys. Chem. B* **113**, 6378-6396 (2009).

20 Fukui, K. Formulation of the reaction coordinate. *J. Phys. Chem.* **74**, 4161-4163 (1970).

21 Fukui, K. The path of chemical reactions - the IRC approach. *Acc. Chem. Res.* **14**, 363-368 (1981).

22 Domingo, L. R., Chamorro, E. & Pérez, P. Understanding the Reactivity of Captodative Ethylenes in Polar Cycloaddition Reactions. A Theoretical Study. *J. Org. Chem.* **73**, 4615-4624 (2008).

23 Domingo, L. R. & Pérez, P. The nucleophilicity N index in organic chemistry. *Org. Biomol. Chem.* **9**, 7168-7175 (2011).
