# Supplementary material for: Programmable site-selective labeling of oligonucleotides based on carbene catalysis
Source: Nat Commun. 2021 Mar 16;12:1681. doi: 10.1038/s41467-021-21839-4 (PMC7966772; doi:10.1038/s41467-021-21839-4)
Supplement: Supplementary file 3 — Description of Additional Supplementary Files [file 41467_2021_21839_MOESM3_ESM.docx]

Description of Additional Supplementary Files

File Name: Supplementary Data 1

Description: Cartesian coordinates of DFT calculations
