# Supplementary material for: Programmable site-selective labeling of oligonucleotides based on carbene catalysis
Source: Nat Commun. 2021 Mar 16;12:1681. doi: 10.1038/s41467-021-21839-4 (PMC7966772; doi:10.1038/s41467-021-21839-4)
Supplement: Supplementary file 4 — Supplementary Data 1 [file 41467_2021_21839_MOESM4_ESM.docx]

**Structures and coordinates of optimized geometries**


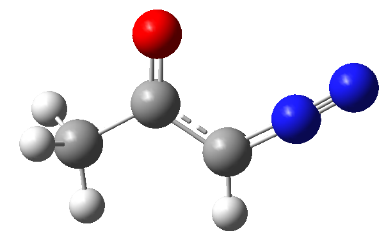


| C | -2.170679 | -0.445886 | 0.000058 |
| --- | --- | --- | --- |
| C | -0.797486 | 0.157067 | 0.000052 |
| C | 0.296883 | -0.789947 | -0.000069 |
| O | -0.622490 | 1.377255 | 0.000145 |
| N | 1.513646 | -0.305763 | -0.000094 |
| N | 2.542513 | 0.169501 | -0.000110 |
| H | -2.158212 | -1.540109 | 0.000011 |
| H | -2.715912 | -0.090581 | 0.883270 |
| H | -2.715950 | -0.090509 | -0.883101 |
| H | 0.204581 | -1.870413 | -0.000155 |


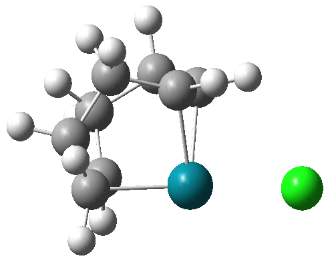


| C | -0.045428 | 1.232044 | 0.880661 |
| --- | --- | --- | --- |
| H | 0.843528 | 1.785698 | 1.196174 |
| C | -0.225540 | -0.052431 | 1.468210 |
| H | 0.552584 | -0.371972 | 2.167035 |
| C | -1.576861 | -0.721604 | 1.641933 |
| H | -1.570524 | -1.312754 | 2.566039 |
| H | -2.349071 | 0.047777 | 1.781941 |
| C | -1.916988 | -1.632795 | 0.458425 |
| H | -3.008773 | -1.749642 | 0.355712 |
| H | -1.520103 | -2.638116 | 0.653823 |
| C | -1.310307 | -1.152822 | -0.836815 |
| H | -1.086467 | -1.938663 | -1.565690 |
| C | -1.358980 | 0.164194 | -1.317570 |
| H | -1.145939 | 0.312828 | -2.380063 |
| C | -2.082186 | 1.294618 | -0.619121 |
| H | -2.519557 | 1.965272 | -1.369364 |
| H | -2.925530 | 0.895895 | -0.039782 |
| C | -1.128345 | 2.087745 | 0.277684 |
| H | -1.678479 | 2.620530 | 1.071858 |
| H | -0.633271 | 2.865386 | -0.320725 |
| Cl | 2.872268 | 0.062384 | 0.274804 |
| Rh | 0.579575 | -0.241255 | -0.455757 |


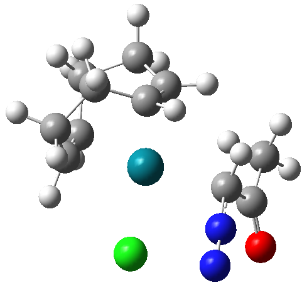


| C | -1.761475 | 0.457748 | -1.001361 |
| --- | --- | --- | --- |
| C | -2.638903 | 1.106694 | 0.020012 |
| O | -3.614590 | 0.545119 | 0.491302 |
| C | -2.203295 | 2.479098 | 0.424558 |
| H | -1.312979 | 2.398274 | 1.065371 |
| H | -1.925886 | 3.084027 | -0.447526 |
| H | -3.005775 | 2.969820 | 0.983293 |
| H | -1.417132 | 1.057712 | -1.847156 |
| N | -2.716612 | -1.721345 | -1.671149 |
| N | -2.268390 | -0.723676 | -1.424349 |
| C | 1.889792 | -1.331605 | 0.528695 |
| H | 1.508981 | -2.265842 | 0.948897 |
| C | 1.873194 | -0.211155 | 1.368971 |
| H | 1.461149 | -0.358548 | 2.370505 |
| C | 2.742886 | 1.011688 | 1.175384 |
| H | 3.026896 | 1.410449 | 2.157346 |
| H | 3.681042 | 0.721649 | 0.684063 |
| C | 2.028150 | 2.105681 | 0.376108 |
| H | 2.758530 | 2.799324 | -0.074206 |
| H | 1.421517 | 2.712742 | 1.064399 |
| C | 1.098359 | 1.564399 | -0.678112 |
| H | 0.318068 | 2.268860 | -0.982393 |
| C | 1.361706 | 0.494850 | -1.549169 |
| H | 0.739385 | 0.428463 | -2.446289 |
| C | 2.683863 | -0.242197 | -1.620829 |
| H | 2.872081 | -0.540157 | -2.660108 |
| H | 3.502230 | 0.439025 | -1.351891 |
| C | 2.697902 | -1.488368 | -0.731459 |
| H | 3.732909 | -1.781724 | -0.486771 |
| H | 2.268263 | -2.331607 | -1.290050 |
| Cl | -1.162586 | -1.636218 | 1.551506 |
| Rh | 0.118074 | -0.180524 | 0.084404 |


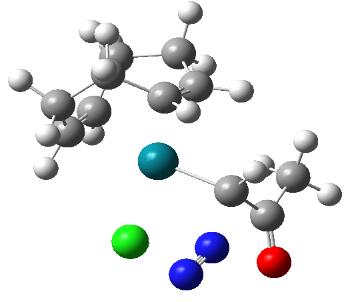


| C | -1.549212 | 0.440945 | -0.903208 |
| --- | --- | --- | --- |
| C | -2.590971 | 1.162778 | -0.144809 |
| O | -3.665022 | 0.690934 | 0.197254 |
| C | -2.159640 | 2.555052 | 0.224927 |
| H | -1.519827 | 2.507187 | 1.116952 |
| H | -1.582775 | 3.037986 | -0.573379 |
| H | -3.048428 | 3.150807 | 0.457701 |
| H | -1.460881 | 0.757350 | -1.953786 |
| N | -2.631609 | -2.206886 | -1.447610 |
| N | -2.445257 | -1.125736 | -1.302802 |
| C | 1.970330 | -1.250504 | 0.778775 |
| H | 1.501309 | -2.076591 | 1.318059 |
| C | 1.997211 | -0.032835 | 1.412647 |
| H | 1.537951 | 0.029698 | 2.401366 |
| C | 2.823693 | 1.145993 | 0.965718 |
| H | 3.170598 | 1.694491 | 1.850797 |
| H | 3.725585 | 0.788749 | 0.452332 |
| C | 2.039677 | 2.107697 | 0.066892 |
| H | 2.732471 | 2.790386 | -0.454168 |
| H | 1.405045 | 2.748194 | 0.698965 |
| C | 1.130049 | 1.435034 | -0.925797 |
| H | 0.397857 | 2.118357 | -1.367487 |
| C | 1.361229 | 0.232845 | -1.618927 |
| H | 0.747372 | 0.068098 | -2.509047 |
| C | 2.657512 | -0.553485 | -1.579163 |
| H | 2.804641 | -1.040972 | -2.551375 |
| H | 3.500099 | 0.141911 | -1.465914 |
| C | 2.689361 | -1.628733 | -0.485948 |
| H | 3.732115 | -1.906938 | -0.258966 |
| H | 2.212599 | -2.540339 | -0.871113 |
| Cl | -1.234708 | -1.262276 | 1.813459 |
| Rh | 0.083936 | -0.148834 | 0.084633 |


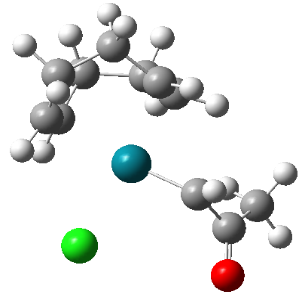


| C | 1.987714 | 1.273224 | -0.116996 |
| --- | --- | --- | --- |
| H | 1.675802 | 2.318351 | -0.068838 |
| C | 1.750038 | 0.615164 | -1.290244 |
| H | 1.245212 | 1.173306 | -2.081008 |
| C | 2.298664 | -0.737451 | -1.659361 |
| H | 2.479968 | -0.761690 | -2.741242 |
| H | 3.277454 | -0.882037 | -1.185795 |
| C | 1.353332 | -1.887833 | -1.300383 |
| H | 1.897389 | -2.846769 | -1.333899 |
| H | 0.570101 | -1.963933 | -2.069487 |
| C | 0.658679 | -1.760153 | 0.025766 |
| H | -0.193001 | -2.434695 | 0.136663 |
| C | 1.161372 | -1.195716 | 1.203467 |
| H | 0.624167 | -1.444739 | 2.120803 |
| C | 2.578125 | -0.698388 | 1.394697 |
| H | 2.864408 | -0.863483 | 2.440825 |
| H | 3.258560 | -1.318445 | 0.796476 |
| C | 2.769123 | 0.785625 | 1.069064 |
| H | 3.840684 | 1.003492 | 0.930646 |
| H | 2.451719 | 1.381082 | 1.936218 |
| Cl | -1.123770 | 2.394362 | -0.549540 |
| Rh | -0.153323 | 0.296017 | 0.184159 |
| C | -1.685190 | -0.346304 | 1.009839 |
| C | -2.867303 | -0.760237 | 0.244698 |
| O | -3.815184 | 0.021952 | 0.301396 |
| C | -2.859365 | -2.000466 | -0.577873 |
| H | -3.849911 | -2.161190 | -1.015909 |
| H | -2.111065 | -1.908105 | -1.377337 |
| H | -2.576222 | -2.867067 | 0.034741 |
| H | -1.801304 | -0.349420 | 2.104940 |


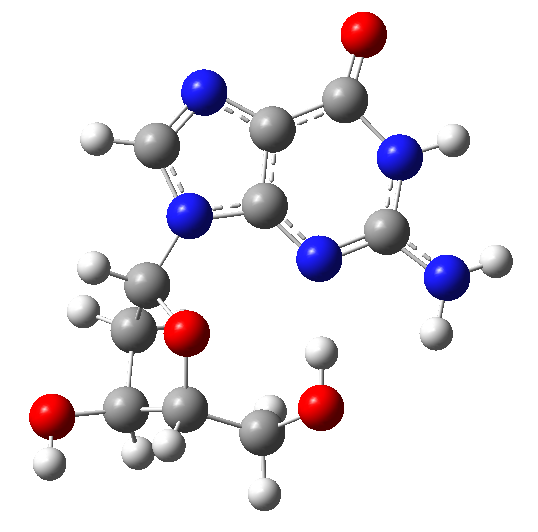


| H | -5.024110 | -0.702507 | -0.071535 |
| --- | --- | --- | --- |
| N | 3.170493 | 0.954650 | 0.231965 |
| C | 1.953158 | 1.574518 | 0.151842 |
| N | 0.820074 | 0.917621 | -0.016895 |
| C | 0.984890 | -0.420111 | -0.107966 |
| C | 2.174472 | -1.135934 | -0.055645 |
| C | 3.398616 | -0.430907 | 0.124155 |
| C | 0.662152 | -2.579944 | -0.349716 |
| N | 1.956049 | -2.490728 | -0.207806 |
| O | 4.544782 | -0.873797 | 0.194248 |
| N | 1.942160 | 2.920309 | 0.208447 |
| N | 0.009436 | -1.365786 | -0.303558 |
| C | -3.317391 | -0.257292 | 0.748502 |
| C | -2.050776 | -1.023816 | 1.081065 |
| C | -1.441458 | -1.219985 | -0.298469 |
| O | -1.811309 | -0.091170 | -1.056573 |
| C | -2.828248 | 0.657763 | -0.367617 |
| C | -2.266855 | 1.963777 | 0.178457 |
| O | -4.271434 | -1.196857 | 0.289084 |
| O | -1.402139 | 2.595802 | -0.738535 |
| H | 4.005735 | 1.523658 | 0.361955 |
| H | 0.100093 | -3.497326 | -0.491439 |
| H | 1.037199 | 3.361875 | 0.325241 |
| H | 2.749883 | 3.416799 | 0.565325 |
| H | -3.702307 | 0.321806 | 1.603046 |
| H | -2.251195 | -1.971607 | 1.590656 |
| H | -1.382664 | -0.418310 | 1.706390 |
| H | -1.824653 | -2.134957 | -0.775087 |
| H | -3.619808 | 0.863500 | -1.102349 |
| H | -3.102036 | 2.645825 | 0.385428 |
| H | -1.752567 | 1.784171 | 1.138289 |
| H | -0.611618 | 2.014373 | -0.754421 |


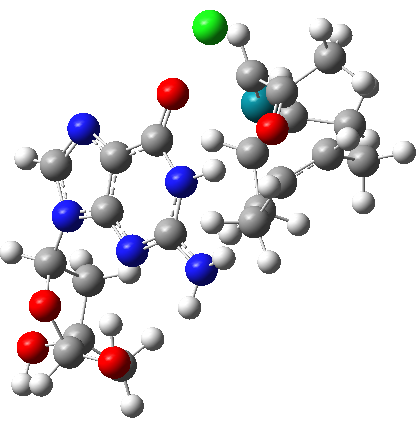


| H | 6.906617 | -2.293260 | -0.186274 |
| --- | --- | --- | --- |
| N | 0.080390 | 2.437991 | -0.073694 |
| C | 1.232426 | 2.229685 | -0.778674 |
| N | 2.264663 | 1.560824 | -0.291948 |
| C | 2.041976 | 1.043899 | 0.929445 |
| C | 0.898086 | 1.185538 | 1.715657 |
| C | -0.194367 | 1.903439 | 1.178778 |
| C | 2.201471 | -0.049094 | 2.827079 |
| N | 1.015818 | 0.489848 | 2.902321 |
| O | -1.324280 | 2.078830 | 1.690619 |
| N | 1.280638 | 2.700344 | -2.038597 |
| N | 2.883971 | 0.252533 | 1.666250 |
| C | 5.060609 | -1.750642 | -0.471568 |
| C | 3.814095 | -1.674595 | 0.388611 |
| C | 4.095687 | -0.446844 | 1.243746 |
| O | 4.900456 | 0.406990 | 0.468034 |
| C | 5.349285 | -0.275038 | -0.718525 |
| C | 4.634360 | 0.253849 | -1.952139 |
| O | 6.072123 | -2.368793 | 0.302756 |
| O | 4.593184 | 1.664142 | -1.967244 |
| H | -0.739674 | 2.874392 | -0.526232 |
| H | 2.659538 | -0.682582 | 3.579941 |
| H | 2.184434 | 2.675705 | -2.498261 |
| H | 0.626397 | 3.415565 | -2.334427 |
| H | 4.892314 | -2.290667 | -1.417068 |
| H | 3.658879 | -2.576920 | 0.988464 |
| H | 2.924320 | -1.505153 | -0.232069 |
| H | 4.617677 | -0.712754 | 2.174922 |
| H | 6.428580 | -0.082829 | -0.801318 |
| H | 5.179792 | -0.080336 | -2.844543 |
| H | 3.613865 | -0.163637 | -2.014699 |
| H | 3.955623 | 1.883216 | -1.255930 |
| C | -3.009152 | 1.259050 | 0.663999 |
| C | -3.327262 | 2.315121 | -0.298123 |
| O | -2.544563 | 3.068174 | -0.873621 |
| C | -4.805485 | 2.368667 | -0.576020 |
| H | -5.217503 | 3.220867 | -0.018057 |
| H | -4.968942 | 2.543047 | -1.645294 |
| H | -5.336254 | 1.463824 | -0.257800 |
| H | -3.516567 | 1.400453 | 1.628194 |
| C | -2.685583 | -2.880513 | -0.255287 |
| H | -3.156787 | -3.210828 | 0.671941 |
| C | -1.345382 | -2.633420 | -0.219031 |
| H | -0.846861 | -2.734149 | 0.749331 |
| C | -0.458870 | -2.394615 | -1.409721 |
| H | 0.522904 | -2.844999 | -1.212907 |
| H | -0.860346 | -2.913474 | -2.288093 |
| C | -0.262382 | -0.903794 | -1.701632 |
| H | 0.190211 | -0.770145 | -2.699735 |
| H | 0.472995 | -0.499111 | -0.986230 |
| C | -1.507843 | -0.065011 | -1.585935 |
| H | -1.303499 | 1.008471 | -1.592391 |
| C | -2.826362 | -0.443619 | -1.894669 |
| H | -3.523286 | 0.374694 | -2.094999 |
| C | -3.247866 | -1.774054 | -2.496171 |
| H | -4.143566 | -1.609184 | -3.108191 |
| H | -2.471595 | -2.117379 | -3.193418 |
| C | -3.570674 | -2.868110 | -1.468935 |
| H | -3.552046 | -3.856094 | -1.958068 |
| H | -4.602070 | -2.729654 | -1.116404 |
| Cl | -2.961184 | -1.109334 | 2.603976 |
| Rh | -2.511764 | -0.569185 | 0.252934 |


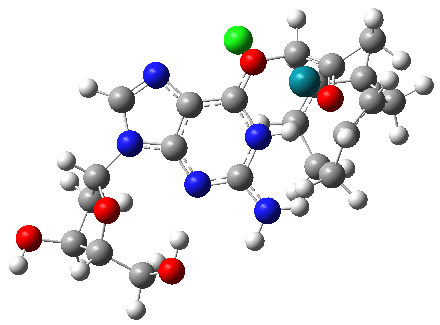


| H | 7.912090 | 0.974648 | -1.208471 |
| --- | --- | --- | --- |
| N | 0.101252 | -0.570224 | 1.445178 |
| C | 1.182750 | 0.239336 | 1.688660 |
| N | 2.336431 | 0.109357 | 1.052606 |
| C | 2.363387 | -0.905977 | 0.179534 |
| C | 1.317323 | -1.790123 | -0.125587 |
| C | 0.116710 | -1.589227 | 0.545076 |
| C | 2.935568 | -2.402506 | -1.320777 |
| N | 1.697378 | -2.719054 | -1.071538 |
| O | -0.924334 | -2.339101 | 0.368488 |
| N | 1.035963 | 1.163112 | 2.648597 |
| N | 3.401716 | -1.313535 | -0.608919 |
| C | 5.988471 | 1.233481 | -1.084572 |
| C | 4.767942 | 0.522780 | -1.637777 |
| C | 4.730777 | -0.727683 | -0.773186 |
| O | 5.231964 | -0.353959 | 0.487445 |
| C | 5.862284 | 0.938359 | 0.405284 |
| C | 5.028083 | 1.984010 | 1.128805 |
| O | 7.128255 | 0.616888 | -1.654319 |
| O | 4.551293 | 1.510497 | 2.369446 |
| H | -0.693975 | -0.607951 | 2.120912 |
| H | 3.590491 | -2.912772 | -2.019506 |
| H | 1.786481 | 1.834781 | 2.766047 |
| H | 0.107641 | 1.425035 | 2.963966 |
| H | 5.976208 | 2.316888 | -1.282858 |
| H | 4.858390 | 0.293822 | -2.704075 |
| H | 3.862494 | 1.121174 | -1.477033 |
| H | 5.346724 | -1.530611 | -1.204418 |
| H | 6.847310 | 0.840530 | 0.883358 |
| H | 5.653926 | 2.864912 | 1.321220 |
| H | 4.190456 | 2.312194 | 0.490339 |
| H | 3.902622 | 0.818691 | 2.129624 |
| C | -2.310825 | -1.901817 | 0.550428 |
| C | -2.741081 | -1.814912 | 1.922351 |
| O | -1.998046 | -1.479284 | 2.870515 |
| C | -4.185833 | -2.111816 | 2.193218 |
| H | -4.238156 | -3.094396 | 2.683321 |
| H | -4.600433 | -1.376230 | 2.893307 |
| H | -4.796323 | -2.151920 | 1.284633 |
| H | -2.887730 | -2.617713 | -0.039551 |
| C | -3.608585 | 1.336637 | -2.029765 |
| H | -3.582886 | 0.769606 | -2.962705 |
| C | -2.406489 | 1.891430 | -1.597037 |
| H | -1.527121 | 1.687128 | -2.213089 |
| C | -2.282652 | 3.039981 | -0.621587 |
| H | -1.428565 | 3.666407 | -0.910267 |
| H | -3.167775 | 3.686120 | -0.690951 |
| C | -2.083341 | 2.548602 | 0.815865 |
| H | -2.359657 | 3.337768 | 1.536613 |
| H | -1.012294 | 2.353255 | 0.981750 |
| C | -2.825320 | 1.273330 | 1.123169 |
| H | -2.451020 | 0.751729 | 2.009719 |
| C | -4.136132 | 0.960060 | 0.719234 |
| H | -4.653934 | 0.191223 | 1.294717 |
| C | -5.054058 | 1.907174 | -0.030631 |
| H | -6.086434 | 1.743227 | 0.305016 |
| H | -4.813588 | 2.944943 | 0.237149 |
| C | -4.983531 | 1.708508 | -1.546344 |
| H | -5.350224 | 2.605807 | -2.073684 |
| H | -5.664155 | 0.893336 | -1.831665 |
| Cl | -1.798466 | -1.280868 | -2.493048 |
| Rh | -2.713813 | -0.038502 | -0.571597 |


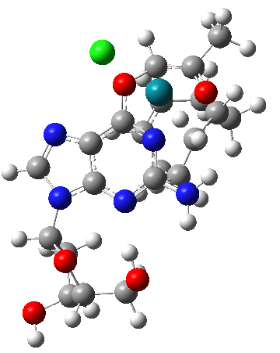


| H | 7.063712 | -2.195423 | -0.249582 |
| --- | --- | --- | --- |
| N | 0.022636 | 2.264565 | -0.005041 |
| C | 1.193558 | 2.141345 | -0.689860 |
| N | 2.248398 | 1.467980 | -0.235033 |
| C | 2.041537 | 0.877701 | 0.942567 |
| C | 0.872275 | 0.931407 | 1.721364 |
| C | -0.185019 | 1.639519 | 1.168334 |
| C | 2.201967 | -0.314182 | 2.778774 |
| N | 0.996062 | 0.171055 | 2.867772 |
| O | -1.360907 | 1.744766 | 1.752092 |
| N | 1.257623 | 2.713737 | -1.901216 |
| N | 2.896043 | 0.077197 | 1.648929 |
| C | 5.205045 | -1.706801 | -0.547979 |
| C | 3.935340 | -1.732283 | 0.281193 |
| C | 4.146494 | -0.543810 | 1.208613 |
| O | 4.917168 | 0.389855 | 0.494101 |
| C | 5.431772 | -0.208785 | -0.711299 |
| C | 4.724883 | 0.348542 | -1.936934 |
| O | 6.224292 | -2.318483 | 0.221066 |
| O | 4.608290 | 1.753470 | -1.882915 |
| H | -0.953730 | 2.800811 | -0.424086 |
| H | 2.670965 | -0.973028 | 3.502666 |
| H | 2.152487 | 2.725880 | -2.377063 |
| H | 0.528669 | 3.346923 | -2.208315 |
| H | 5.085833 | -2.203291 | -1.524111 |
| H | 3.804873 | -2.672757 | 0.825738 |
| H | 3.054252 | -1.563192 | -0.352245 |
| H | 4.665423 | -0.836377 | 2.133231 |
| H | 6.502453 | 0.036620 | -0.750923 |
| H | 5.311912 | 0.090730 | -2.828195 |
| H | 3.731854 | -0.120109 | -2.052331 |
| H | 3.955331 | 1.906693 | -1.170038 |
| C | -2.602370 | 1.557140 | 1.025441 |
| C | -2.972886 | 2.439571 | -0.018663 |
| O | -2.175856 | 3.128846 | -0.737389 |
| C | -4.430308 | 2.528581 | -0.335414 |
| H | -4.796807 | 3.502411 | 0.018642 |
| H | -4.587690 | 2.496638 | -1.420148 |
| H | -5.015302 | 1.739193 | 0.147646 |
| H | -3.367428 | 1.407401 | 1.785869 |
| C | -3.030619 | -2.601321 | -0.327164 |
| H | -3.536658 | -2.983320 | 0.561742 |
| C | -1.643585 | -2.469455 | -0.254246 |
| H | -1.179108 | -2.732817 | 0.699826 |
| C | -0.714875 | -2.404106 | -1.446702 |
| H | 0.225554 | -2.911541 | -1.195001 |
| H | -1.146151 | -2.967205 | -2.285524 |
| C | -0.413587 | -0.960449 | -1.863757 |
| H | -0.079322 | -0.923084 | -2.915183 |
| H | 0.434159 | -0.583637 | -1.266967 |
| C | -1.571017 | -0.020013 | -1.644138 |
| H | -1.280687 | 1.031408 | -1.619622 |
| C | -2.922712 | -0.283149 | -1.926152 |
| H | -3.578619 | 0.585453 | -2.033815 |
| C | -3.426978 | -1.547226 | -2.595241 |
| H | -4.281700 | -1.294223 | -3.235870 |
| H | -2.652325 | -1.937975 | -3.269391 |
| C | -3.854542 | -2.614412 | -1.585234 |
| H | -3.834141 | -3.616015 | -2.047700 |
| H | -4.900178 | -2.436608 | -1.296500 |
| Cl | -2.972501 | -1.159391 | 2.545668 |
| Rh | -2.556013 | -0.529026 | 0.193475 |


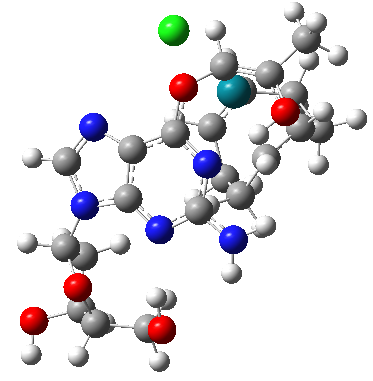


| H | 6.967010 | -2.136879 | 0.101026 |
| --- | --- | --- | --- |
| N | -0.086897 | 2.377593 | -0.337902 |
| C | 1.085206 | 2.166730 | -0.982158 |
| N | 2.169258 | 1.578686 | -0.460598 |
| C | 1.991659 | 1.160877 | 0.792625 |
| C | 0.824113 | 1.311751 | 1.557791 |
| C | -0.237643 | 1.921539 | 0.897378 |
| C | 2.180055 | 0.270921 | 2.789709 |
| N | 0.964966 | 0.743268 | 2.809899 |
| O | -1.411944 | 2.091898 | 1.527216 |
| N | 1.166524 | 2.622702 | -2.255560 |
| N | 2.866733 | 0.495846 | 1.613529 |
| C | 5.082309 | -1.760876 | -0.206623 |
| C | 3.861818 | -1.587861 | 0.675836 |
| C | 4.105037 | -0.210420 | 1.277948 |
| O | 4.836761 | 0.520948 | 0.326586 |
| C | 5.267993 | -0.346595 | -0.740534 |
| C | 4.460302 | -0.092853 | -2.002751 |
| O | 6.153832 | -2.157850 | 0.629631 |
| O | 4.356054 | 1.284984 | -2.284722 |
| H | -1.632045 | 2.857559 | -0.964632 |
| H | 2.663523 | -0.258721 | 3.605093 |
| H | 1.926897 | 2.261400 | -2.820324 |
| H | 0.295189 | 2.819175 | -2.734665 |
| H | 4.920466 | -2.482765 | -1.022744 |
| H | 3.772262 | -2.369097 | 1.437564 |
| H | 2.944569 | -1.576434 | 0.071495 |
| H | 4.669922 | -0.274091 | 2.219742 |
| H | 6.326981 | -0.115911 | -0.924101 |
| H | 4.966503 | -0.578697 | -2.847519 |
| H | 3.458083 | -0.549578 | -1.916082 |
| H | 3.758295 | 1.620874 | -1.582709 |
| C | -2.647937 | 1.675931 | 0.980076 |
| C | -3.226830 | 2.155113 | -0.176834 |
| O | -2.615281 | 2.800298 | -1.158905 |
| C | -4.691339 | 1.997467 | -0.382512 |
| H | -5.162421 | 2.986007 | -0.285296 |
| H | -4.911756 | 1.630849 | -1.392142 |
| H | -5.135749 | 1.322317 | 0.355186 |
| H | -3.318717 | 1.429537 | 1.798220 |
| C | -2.762501 | -2.670828 | -0.011962 |
| H | -3.236895 | -2.981267 | 0.921048 |
| C | -1.382585 | -2.438193 | 0.032139 |
| H | -0.904112 | -2.559318 | 1.007688 |
| C | -0.460570 | -2.477589 | -1.166580 |
| H | 0.507018 | -2.894978 | -0.859518 |
| H | -0.862716 | -3.166244 | -1.921607 |
| C | -0.247018 | -1.083106 | -1.760262 |
| H | 0.090231 | -1.149202 | -2.809158 |
| H | 0.570187 | -0.582801 | -1.215029 |
| C | -1.466998 | -0.206457 | -1.656145 |
| H | -1.252543 | 0.860795 | -1.734844 |
| C | -2.794735 | -0.589783 | -1.900501 |
| H | -3.499207 | 0.218939 | -2.109127 |
| C | -3.222947 | -1.955291 | -2.396291 |
| H | -4.090059 | -1.839252 | -3.058650 |
| H | -2.427357 | -2.390574 | -3.015856 |
| C | -3.591563 | -2.893500 | -1.246456 |
| H | -3.529848 | -3.948079 | -1.564394 |
| H | -4.641655 | -2.723590 | -0.969253 |
| Cl | -2.765791 | -0.951256 | 2.653094 |
| Rh | -2.424178 | -0.541986 | 0.244014 |


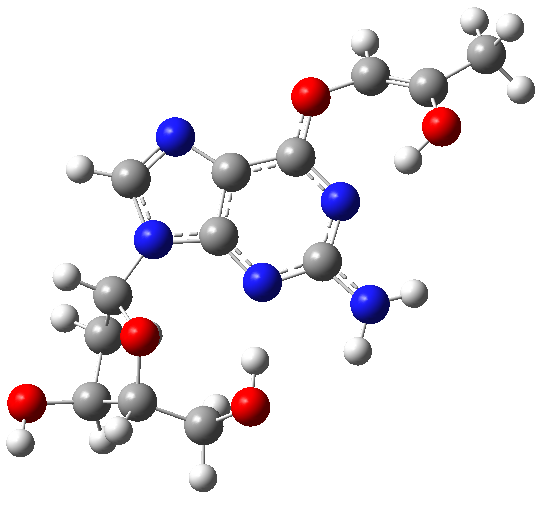


| H | -6.229024 | -0.252722 | 0.064153 |
| --- | --- | --- | --- |
| N | 2.151080 | 0.628190 | 0.153094 |
| C | 0.982885 | 1.317087 | 0.096418 |
| N | -0.239160 | 0.795282 | -0.058954 |
| C | -0.225782 | -0.535555 | -0.182175 |
| C | 0.903609 | -1.356512 | -0.167641 |
| C | 2.119770 | -0.689853 | 0.015423 |
| C | -0.731986 | -2.656257 | -0.448643 |
| N | 0.566974 | -2.686759 | -0.333165 |
| O | 3.235127 | -1.415799 | 0.042265 |
| N | 1.078517 | 2.665587 | 0.165624 |
| N | -1.281889 | -1.392698 | -0.371387 |
| C | -4.454037 | -0.024856 | 0.826096 |
| C | -3.266099 | -0.932830 | 1.082991 |
| C | -2.713989 | -1.122141 | -0.321525 |
| O | -2.994283 | 0.067688 | -1.021451 |
| C | -3.904175 | 0.886140 | -0.264331 |
| C | -3.187371 | 2.098297 | 0.314643 |
| O | -5.513503 | -0.838705 | 0.356849 |
| O | -2.316676 | 2.694229 | -0.620773 |
| H | -1.374152 | -3.518889 | -0.595236 |
| H | 0.222574 | 3.184646 | 0.321551 |
| H | 1.941075 | 3.066635 | 0.511290 |
| H | -4.752866 | 0.549900 | 1.717427 |
| H | -3.551993 | -1.879315 | 1.552545 |
| H | -2.523210 | -0.432186 | 1.716416 |
| H | -3.188840 | -1.976704 | -0.826413 |
| H | -4.691883 | 1.208629 | -0.959976 |
| H | -3.938194 | 2.843763 | 0.608004 |
| H | -2.636104 | 1.813821 | 1.227417 |
| H | -1.575762 | 2.053346 | -0.687750 |
| C | 4.492176 | -0.954973 | 0.459191 |
| C | 5.150492 | 0.145933 | 0.069089 |
| O | 4.670769 | 1.131461 | -0.730644 |
| C | 6.552142 | 0.383119 | 0.510405 |
| H | 7.205464 | 0.498529 | -0.365053 |
| H | 6.618872 | 1.313971 | 1.089154 |
| H | 6.932288 | -0.441837 | 1.121152 |
| H | 4.968506 | -1.695168 | 1.093252 |
| H | 3.687305 | 1.139779 | -0.624024 |


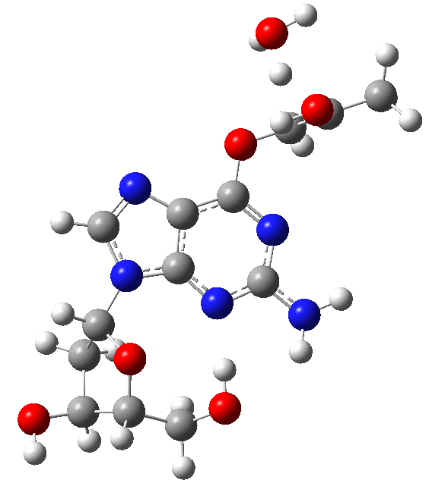


| H | 6.565874 | -0.725653 | 0.167806 |
| --- | --- | --- | --- |
| N | -1.663750 | 1.023316 | -0.389300 |
| C | -0.422952 | 1.566742 | -0.381844 |
| N | 0.729479 | 0.926826 | -0.138934 |
| C | 0.562541 | -0.376585 | 0.093887 |
| C | -0.655516 | -1.061286 | 0.116627 |
| C | -1.774184 | -0.263961 | -0.137382 |
| C | 0.811400 | -2.507546 | 0.564627 |
| N | -0.480238 | -2.400637 | 0.412943 |
| O | -2.991797 | -0.850763 | -0.111830 |
| N | -0.342060 | 2.886765 | -0.686765 |
| N | 1.506854 | -1.329042 | 0.390130 |
| C | 4.863212 | -0.367638 | -0.701715 |
| C | 3.603181 | -1.167090 | -0.976235 |
| C | 2.960866 | -1.216063 | 0.402644 |
| O | 3.332652 | -0.023721 | 1.055332 |
| C | 4.358074 | 0.654483 | 0.308506 |
| C | 3.804474 | 1.897600 | -0.373643 |
| O | 5.810827 | -1.254286 | -0.134988 |
| O | 2.953175 | 2.633969 | 0.475889 |
| H | 1.344946 | -3.420509 | 0.810520 |
| H | 0.529864 | 3.354622 | -0.467051 |
| H | -1.189448 | 3.432897 | -0.597306 |
| H | 5.261683 | 0.121053 | -1.604959 |
| H | 3.815948 | -2.163437 | -1.376147 |
| H | 2.949317 | -0.635765 | -1.679278 |
| H | 3.313275 | -2.085587 | 0.977524 |
| H | 5.139547 | 0.935132 | 1.028642 |
| H | 4.644936 | 2.543044 | -0.661555 |
| H | 3.276320 | 1.617385 | -1.300680 |
| H | 2.147454 | 2.076073 | 0.531970 |
| C | -4.099760 | -0.074554 | -0.498033 |
| C | -4.794001 | 0.570049 | 0.503095 |
| O | -4.538457 | 0.382624 | 1.780300 |
| C | -5.970504 | 1.411845 | 0.216341 |
| H | -5.808454 | 2.417019 | 0.625327 |
| H | -6.153539 | 1.482702 | -0.859821 |
| H | -6.854933 | 0.994190 | 0.714190 |
| H | -4.054123 | 0.371780 | -1.492171 |
| O | -5.957877 | -1.856589 | -1.141573 |
| H | -3.741168 | -0.175345 | 1.890332 |
| H | -5.165063 | -1.064194 | -0.827662 |
| H | -5.797881 | -2.069767 | -2.084045 |
| H | -6.825715 | -1.402074 | -1.118451 |


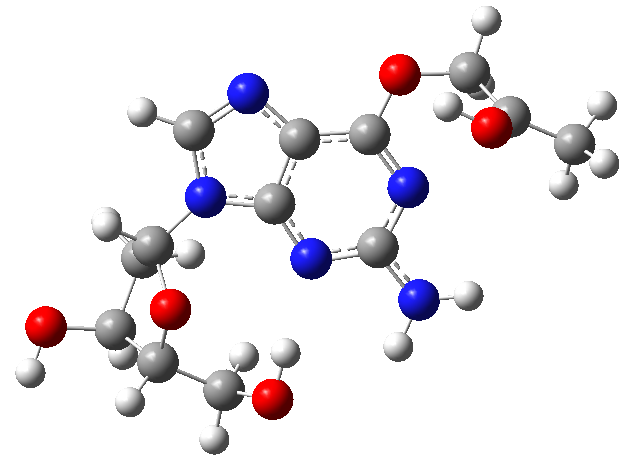


| H | 6.180348 | -0.117684 | 0.179487 |
| --- | --- | --- | --- |
| N | -2.207486 | 0.518596 | -0.357260 |
| C | -1.063239 | 1.241311 | -0.269019 |
| N | 0.164119 | 0.745776 | -0.061778 |
| C | 0.186441 | -0.582845 | 0.050794 |
| C | -0.919113 | -1.440116 | -0.016659 |
| C | -2.130335 | -0.790917 | -0.231167 |
| C | 0.738049 | -2.691025 | 0.329747 |
| N | -0.553014 | -2.760308 | 0.158866 |
| O | -3.275227 | -1.514899 | -0.290072 |
| N | -1.176460 | 2.578323 | -0.438932 |
| N | 1.255458 | -1.412263 | 0.279959 |
| C | 4.448872 | 0.086679 | -0.684672 |
| C | 3.306283 | -0.846232 | -1.038714 |
| C | 2.679442 | -1.096682 | 0.324219 |
| O | 2.879750 | 0.078650 | 1.074562 |
| C | 3.810969 | 0.945140 | 0.400428 |
| C | 3.095458 | 2.157713 | -0.179907 |
| O | 5.504415 | -0.712220 | -0.181834 |
| O | 2.147556 | 2.690885 | 0.717913 |
| H | 1.398042 | -3.535069 | 0.503283 |
| H | -0.380327 | 3.148562 | -0.177949 |
| H | -2.096894 | 2.991149 | -0.353730 |
| H | 4.779468 | 0.698806 | -1.539076 |
| H | 3.648069 | -1.768230 | -1.519241 |
| H | 2.586119 | -0.347063 | -1.699038 |
| H | 3.149441 | -1.952487 | 0.831247 |
| H | 4.547434 | 1.263010 | 1.151821 |
| H | 3.839808 | 2.936035 | -0.393704 |
| H | 2.616946 | 1.893403 | -1.138417 |
| H | 1.430928 | 2.021300 | 0.725247 |
| C | -4.392297 | -0.796713 | -0.771763 |
| C | -4.839411 | 0.257503 | 0.173855 |
| O | -4.693051 | 0.113008 | 1.429079 |
| C | -5.555309 | 1.430734 | -0.305680 |
| H | -4.817102 | 2.083928 | -0.796577 |
| H | -6.264480 | 1.129768 | -1.088232 |
| H | -6.050192 | 1.966565 | 0.506655 |
| H | -4.213429 | -0.360844 | -1.761169 |
| H | -4.214318 | -0.715954 | 1.671174 |
| H | -5.220595 | -1.516762 | -0.853713 |


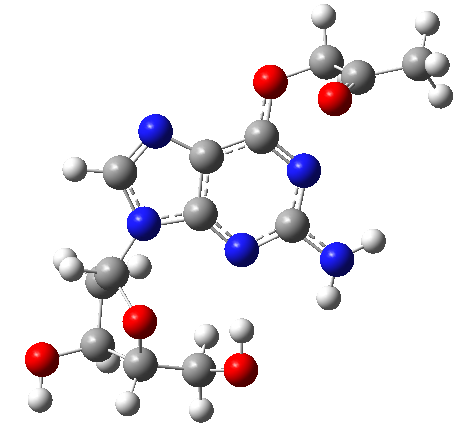


| H | -6.188302 | -0.332824 | -0.263836 |
| --- | --- | --- | --- |
| N | 2.146960 | 0.730021 | 0.518741 |
| C | 0.981832 | 1.391758 | 0.328699 |
| N | -0.211142 | 0.851695 | 0.041593 |
| C | -0.169359 | -0.480283 | -0.052474 |
| C | 0.965500 | -1.279958 | 0.096751 |
| C | 2.142027 | -0.585097 | 0.397044 |
| C | -0.609714 | -2.615090 | -0.328380 |
| N | 0.670813 | -2.619498 | -0.078282 |
| O | 3.261412 | -1.296267 | 0.583941 |
| N | 1.038768 | 2.746380 | 0.403089 |
| N | -1.185600 | -1.361153 | -0.337767 |
| C | -4.474045 | -0.086103 | 0.625293 |
| C | -3.293218 | -0.978886 | 0.955382 |
| C | -2.625197 | -1.130144 | -0.404165 |
| O | -2.887534 | 0.058797 | -1.111386 |
| C | -3.863918 | 0.849790 | -0.410434 |
| C | -3.211906 | 2.065693 | 0.232200 |
| O | -5.484767 | -0.910015 | 0.072369 |
| O | -2.298498 | 2.695276 | -0.638018 |
| H | -1.217918 | -3.492538 | -0.525067 |
| H | 0.157835 | 3.236436 | 0.504078 |
| H | 1.845260 | 3.153529 | 0.858787 |
| H | -4.844280 | 0.470359 | 1.501064 |
| H | -3.599222 | -1.940486 | 1.379604 |
| H | -2.615344 | -0.481682 | 1.660210 |
| H | -3.032590 | -1.991738 | -0.954315 |
| H | -4.605781 | 1.168710 | -1.156213 |
| H | -3.995713 | 2.790171 | 0.490262 |
| H | -2.711920 | 1.777092 | 1.172858 |
| H | -1.537440 | 2.074371 | -0.663122 |
| C | 4.459938 | -0.586338 | 0.842020 |
| C | 5.025364 | 0.059934 | -0.399334 |
| O | 4.546774 | -0.149827 | -1.500351 |
| C | 6.191015 | 0.960417 | -0.160430 |
| H | 6.718330 | 1.164484 | -1.096798 |
| H | 5.816739 | 1.908399 | 0.252035 |
| H | 6.878844 | 0.537390 | 0.582577 |
| H | 4.325636 | 0.160374 | 1.634876 |
| H | 5.178263 | -1.332024 | 1.205908 |


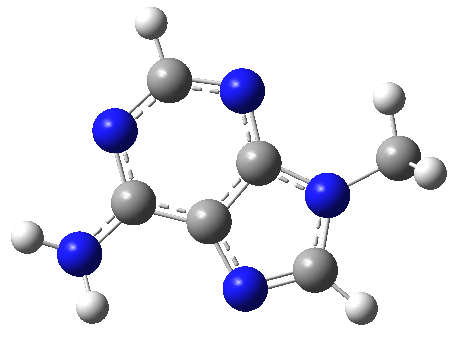


| N | -2.060779 | 1.025817 | 0.001541 |
| --- | --- | --- | --- |
| C | -1.076380 | 1.932542 | 0.002063 |
| N | 0.241042 | 1.746319 | 0.000266 |
| C | 0.541066 | 0.440484 | -0.001821 |
| C | -0.361169 | -0.619997 | -0.004497 |
| C | -1.723813 | -0.277094 | -0.004554 |
| N | 1.783219 | -0.142974 | 0.001351 |
| C | 1.560614 | -1.495690 | 0.000782 |
| N | 0.294250 | -1.835094 | -0.002840 |
| N | -2.702666 | -1.205194 | -0.045376 |
| C | 3.070196 | 0.527469 | 0.003261 |
| H | -1.407988 | 2.971836 | 0.006401 |
| H | 2.395588 | -2.189437 | 0.003030 |
| H | -3.647484 | -0.903464 | 0.160410 |
| H | -2.469154 | -2.167983 | 0.162869 |
| H | 2.896615 | 1.606345 | 0.006534 |
| H | 3.641944 | 0.254327 | 0.895544 |
| H | 3.641924 | 0.259972 | -0.890779 |


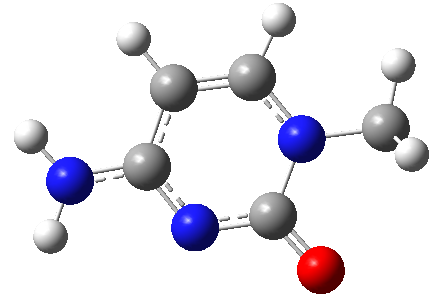


| C | -1.084463 | 1.311541 | 0.000063 |
| --- | --- | --- | --- |
| C | 0.258227 | 1.493005 | 0.000076 |
| C | 0.619918 | -0.884665 | -0.000045 |
| N | -0.720009 | -1.082171 | -0.000049 |
| C | -1.544404 | -0.037401 | 0.000001 |
| N | -2.865445 | -0.279653 | 0.000003 |
| O | 1.439897 | -1.812066 | -0.000108 |
| N | 1.111264 | 0.436124 | 0.000035 |
| C | 2.554579 | 0.627926 | 0.000029 |
| H | -1.773117 | 2.150456 | 0.000100 |
| H | 0.716945 | 2.479142 | 0.000125 |
| H | -3.199551 | -1.234596 | -0.000094 |
| H | -3.535763 | 0.477407 | -0.000017 |
| H | 2.768287 | 1.699298 | 0.000137 |
| H | 3.000125 | 0.171045 | 0.889515 |
| H | 3.000094 | 0.171238 | -0.889573 |


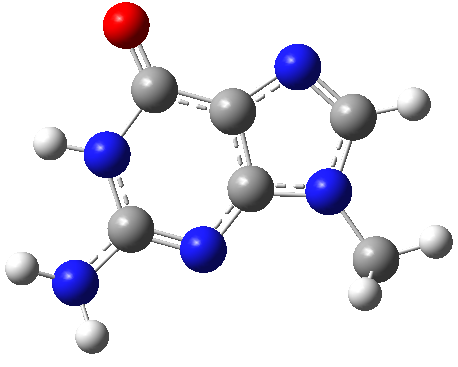


| N | -1.959336 | 0.240292 | -0.000926 |
| --- | --- | --- | --- |
| C | -1.542623 | -1.066116 | -0.002840 |
| N | -0.273961 | -1.420368 | 0.001166 |
| C | 0.557649 | -0.356935 | -0.000102 |
| C | 0.241982 | 0.997032 | -0.000171 |
| C | -1.128187 | 1.377151 | 0.000341 |
| C | 2.352558 | 0.899316 | 0.001605 |
| N | 1.382188 | 1.776083 | 0.000957 |
| O | -1.626796 | 2.504449 | 0.001940 |
| N | -2.512866 | -2.007894 | -0.061621 |
| N | 1.923427 | -0.407159 | 0.001300 |
| C | 2.751767 | -1.596312 | 0.000639 |
| H | -2.958525 | 0.437244 | -0.007559 |
| H | 3.413793 | 1.126420 | 0.002102 |
| H | -2.222037 | -2.949924 | 0.173340 |
| H | -3.449468 | -1.753016 | 0.232307 |
| H | 2.557131 | -2.200759 | -0.891171 |
| H | 3.799826 | -1.286201 | -0.000205 |
| H | 2.558614 | -2.200857 | 0.892707 |


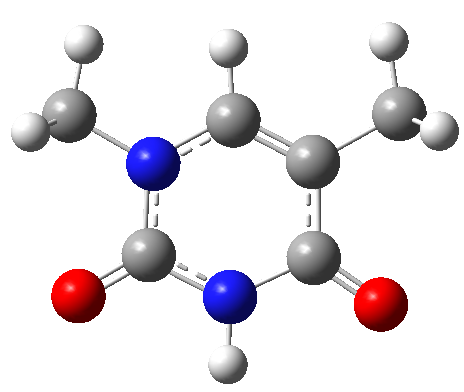


| C | 1.234237 | 0.655612 | -0.000083 |
| --- | --- | --- | --- |
| N | -0.013923 | -1.380040 | 0.000192 |
| C | 1.247622 | -0.790162 | 0.000055 |
| O | 2.258666 | -1.492078 | 0.000067 |
| O | -2.297927 | -1.365342 | 0.000195 |
| C | 2.536843 | 1.380913 | -0.000163 |
| C | 0.036214 | 1.283158 | -0.000130 |
| N | -1.170815 | 0.626612 | -0.000024 |
| C | -1.238392 | -0.749600 | 0.000130 |
| C | -2.435220 | 1.356342 | -0.000169 |
| H | -0.045490 | -2.397601 | 0.000277 |
| H | 2.379751 | 2.465346 | -0.000217 |
| H | 3.138236 | 1.121153 | 0.881523 |
| H | 3.138193 | 1.121057 | -0.881848 |
| H | -0.036445 | 2.368861 | -0.000242 |
| H | -2.217590 | 2.426208 | -0.000192 |
| H | -3.018532 | 1.105336 | -0.891545 |
| H | -3.018688 | 1.105415 | 0.891125 |
